# Chemiluminescent probes for imaging H_2_S in living animals†
†Electronic supplementary information (ESI) available: Detailed experimental procedures, characterization of compounds, computational methods, optimized geometries, atomic charges, supplementary figures, and replicate images. See DOI: 10.1039/c4sc03516j



**DOI:** 10.1039/c4sc03516j

**Published:** 2015-01-02

**Authors:** J. Cao, R. Lopez, J. M. Thacker, J. Y. Moon, C. Jiang, S. N. S. Morris, J. H. Bauer, P. Tao, R. P. Mason, A. R. Lippert

**Affiliations:** a Department of Chemistry , Southern Methodist University , Dallas , TX 75275-0314 , USA . Email: alippert@smu.edu; b Center for Drug Discovery , Design, and Delivery (CD4) , Southern Methodist University , Dallas , TX 75275-0314 , USA; c Laboratory of Prognostic Radiology , Pre-clinical Imaging Section , Department of Radiology , UT Southwestern Medical Center , Dallas , TX 75390-9058 , USA; d Hockaday School , Dallas , TX 75229 , USA; e Department of Biological Sciences , Southern Methodist University , Dallas , TX 75275-0314 , USA

## Abstract

Hydrogen sulphide (H_2_S) is an endogenous mediator of human health and disease, but precise measurement in living cells and animals remains a considerable challenge. We report the total chemical synthesis and characterization of three 1,2-dioxetane chemiluminescent reaction-based H_2_S probes, **CHS-1**, **CHS-2**, and **CHS-3**. Upon treatment with H_2_S at physiological pH, these probes display instantaneous light emission that is sustained for over an hour with high selectivity against other reactive sulphur, oxygen, and nitrogen species. Analysis of the phenol/phenolate equilibrium and atomic charges has provided a generally applicable predictive model to design improved chemiluminescent probes. The utility of these chemiluminescent reagents was demonstrated by applying **CHS-3** to detect cellularly generated H_2_S using a multi-well plate reader and to image H_2_S in living mice using CCD camera technology.

## Abstract 2

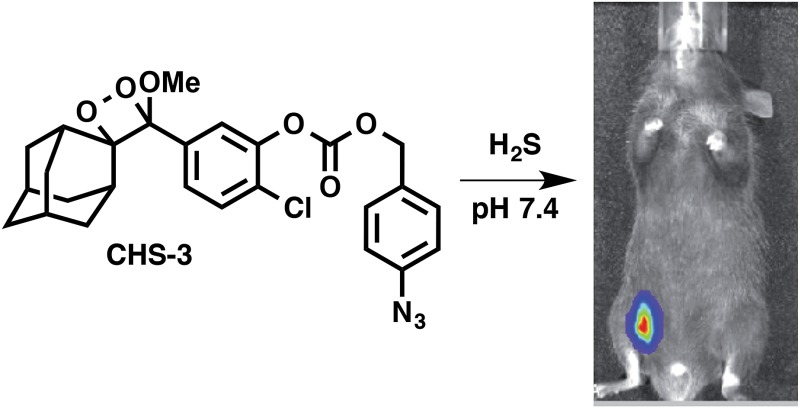
Responsive 1,2-dioxetane chemiluminescent probes have been developed that display instantaneous, sensitive, and selective responses to H_2_S and are capable of imaging H_2_S in living mice.

## Introduction

Hydrogen sulphide (H_2_S) is increasingly recognized as an important mediator of mammalian physiology and pathology, playing roles in vasorelaxation,^1^ angiogenesis,^2^ redox regulation,^3^ neuromodulation,^4^ lifespan,^5^ Huntington's disease,^6^ Down syndrome,^7^ diabetes,^8^ and cancer.^9^ In mammals, endogenous H_2_S is generated from cystathionine β-synthase (CBS),^10^ cystathionine γ-lyase (CSE),^11^ and 3-mercaptopyruvate sulphur transferase (3MST).^12^ Similar to its reactive cousins, nitric oxide (NO) and hydrogen peroxide (H_2_O_2_),^13^ H_2_S mediates cellular function *via* direct chemical interaction with biological molecules and can have widely disparate effects that depend on concentration, tissue localization, and the molecular environment.^14–16^ For example, H_2_S in colon cancer promotes tumour growth by stimulating angiogenesis and supporting cellular energetics,^17^ whilst H_2_S in prostate cancer slows cell growth, disrupts androgen receptor transactivation, and reduces angiogenesis by inhibiting the function of hypoxia-inducible factor 1.^18^ Given the delicate site- and concentration-dependent actions of H_2_S, easy methods for accurate spatiotemporal detection in living cells and animals are in urgent demand and promise to significantly contribute to an increased understanding of this reactive signalling molecule.

Common methods of H_2_S detection, including the methylene blue assay, ion-selective electrodes, amperometric sensors, and gas chromatography, have varying strengths and weaknesses, but all generally fall short of being able to detect H_2_S inside of intact living organisms.^19^ Fluorescent probes offer the ability to target specific analytes^20,21^ and there has been an explosion of recent activity in the development of dyes responsive to H_2_S and its derivatives.^22–24^ Unfortunately, imaging endogenous H_2_S remains rare, and often requires advanced confocal microscopy setups to allow precise same-cell tracking or two-photon excitation coupled with ratiometric imaging.^25,26^ This instrumentation is unavailable to many researchers, reducing the potential impact of these newly developed tools.^27^ Furthermore, there are few examples of *in vivo* imaging of H_2_S,^28^ and imaging deep mammalian tissue remains a frontier field for H_2_S detection. At the heart of the matter is a lack of sensitivity and depth penetration due to background autofluorescence, light scattering, photoactivation/photobleaching, and probe kinetics that requires long incubation times to accumulate signal. In order to address these technological challenges and develop tools to better elucidate the precise mechanisms of H_2_S production and function, we have herein developed a series of selective and sensitive chemiluminescent probes for rapid imaging of H_2_S in living animals.

Triggered chemiluminescence emission can provide a highly sensitive readout of biological analytes.^29^ Chemiluminescence doesn't require light excitation, thereby drastically reducing background from autofluorescence and photoactivation of azide functional groups. Whereas bioluminescence (chemiluminescence derived from living systems that express bioluminescent enzymes such as luciferase) has found wide application for preclinical analysis of biological parameters using genetically modified organisms,^30^ small molecule chemiluminescence can be used with wild-type animals and opens up exciting opportunities for clinical imaging. Recently, there have appeared select examples of chemiluminescent agents for the detection of H_2_S *in vitro*.^31,32^ Although quite promising, the need for enzymatic additives and alkaline conditions introduces cytotoxicity and can potentially release sulphide from proteins and other base-labile sulphur pools,^33,34^ ultimately hindering their potential for whole animal imaging.

**Scheme 1:**
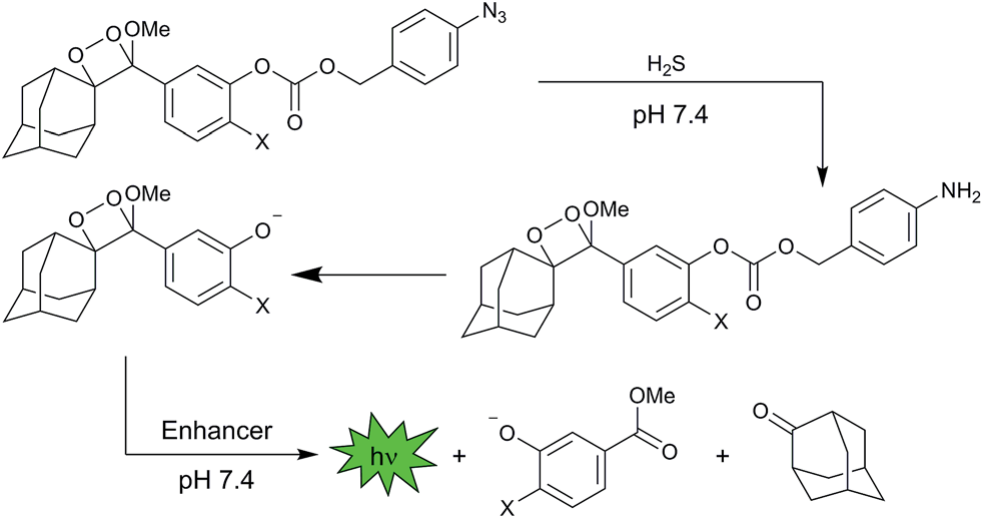
Spiroadamantane 1,2-dioxetanes for chemiluminescent H_2_S detection at neutral pH.

To overcome these issues and expand the scope of chemiluminescent detection technology for small molecule biological analytes, we focused on using sterically stabilized 1,2-dioxetane systems^35^ to develop a series of new chemiluminescent H_2_S probes that display instantaneous light production under biologically compatible conditions. Sterically stabilized dioxetanes have been used for femtogram detection of enzymatic analytes^36^ and have demonstrated potential for *in vivo* imaging.^37^ We introduce three first generation chemiluminescent H_2_S probes, **CHS-1**, **CHS-2**, and **CHS-3**, derived from sterically stabilized spiroadamantane 1,2-dioxetane scaffolds modified with self-immolative 4-azidobenzyl carbonates as the H_2_S response site (Scheme 1).^38^ This article reports their synthetic preparation, optical response and selectivity for H_2_S, experimental and computational mechanistic investigations of their chemiluminescent response, detection of cellular H_2_S using a multi-well plate reader, and a noteworthy demonstration of whole animal chemiluminescent imaging of H_2_S.

## Results and discussion

### Design and synthesis of **CHS-1**, **CHS-2**, and **CHS-3**

The **CHS** probe series was designed such that chemiluminescent emission would be initiated by the H_2_S-mediated reduction of the azide group, followed by self-immolative carbonate cleavage yielding the free phenolate bearing the 1,2-dioxetane (Scheme 1). The negatively charged phenolate will emit light spontaneously upon decomposition *via* an intramolecular chemically initiated electron exchange luminescence (CIEEL) mechanism.^39–41^ This newly produced light can be observed directly or by transfer of its energy to acceptor molecules like quantum dots, rhodamine, or fluorescein.^42^ In our studies, we employ a commercially available Emerald II Enhancer solution that consists of a cationic polymer and a dye with similar photophysical properties to fluorescein. The polymer reduces water-induced quenching by providing a hydrophobic environment for the chemiluminescent reaction and the dye effectively red shifts the luminescence emission making it more amenable for biological imaging applications.

**Scheme 2:**
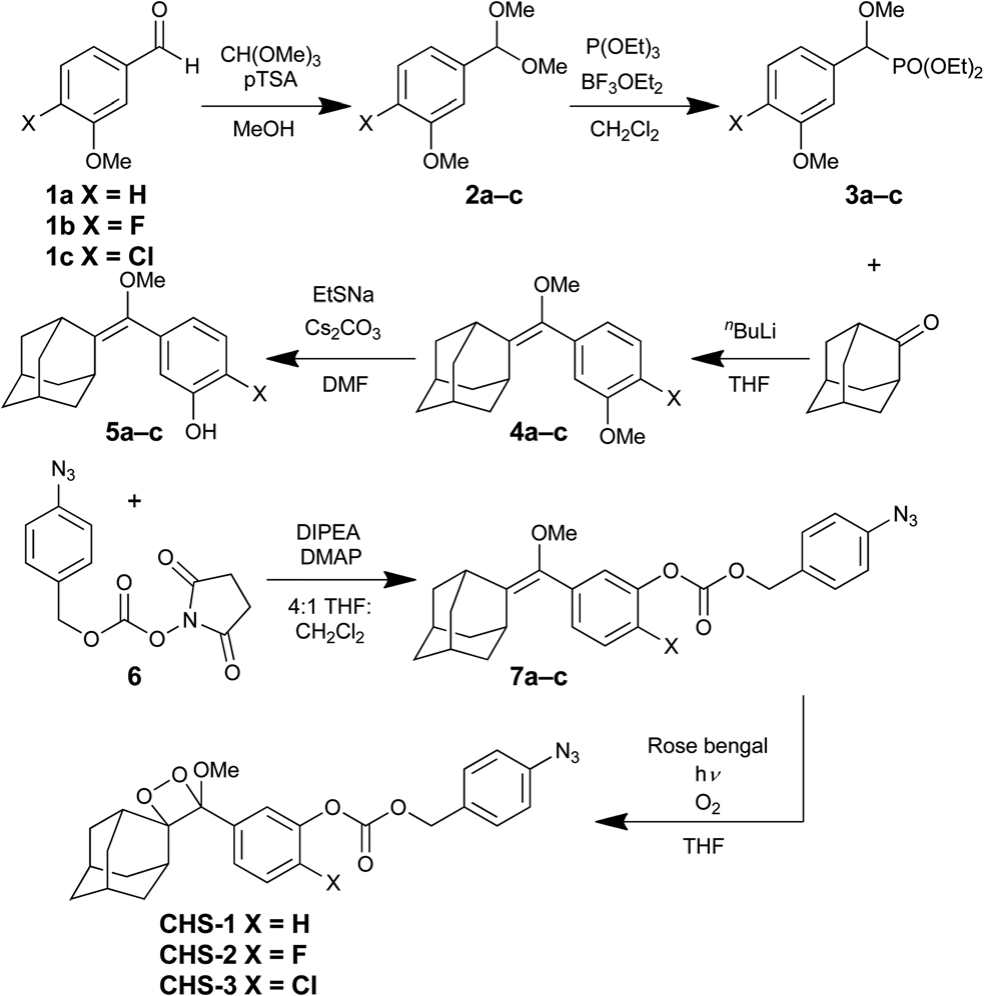
Syntheses of **CHS-1**, **CHS-2**, and **CHS-3**.

By adapting a literature procedure,^43^ we optimized an efficient and modular synthetic route to access the probes **CHS-1**, **CHS-2**, and **CHS-3** (Scheme 2). First, unsubstituted, fluorinated, and chlorinated 3-methoxybenzaldehyde derivatives **1a–c** were reacted with trimethyl orthoformate in the presence of *p*-toluenesulfonic acid to give acetals **2a–c**. These acetals were subjected to triethyl phosphite and boron trifluoride diethyl etherate at 0 °C, which afforded the diethyl methoxy (3-methoxyphenyl) methyl phosphonates **3a–c**. Next, enol ethers **4a–c** were obtained through Horner–Wadsworth–Emmons reaction by treating phosphonates **3a–c** with ^*n*^BuLi and 2-adamantanone.^44^ Nucleophilic demethylation using sodium ethanethiolate provided the phenols **5a–c**. The activated ester **6**, prepared according to analogous literature procedures, was coupled to the phenols to provide **7a–c**, azide-bearing precursors to the final 1,2-dioxetanes.^45,46^ Finally, a [2 + 2] cycloaddition with singlet oxygen was accomplished by bubbling oxygen through a solution of **7a–c** and the sensitizer Rose bengal with visible light irradiation, delivering the chemiluminescent probes **CHS-1**, **CHS-2**, and **CHS-3**.

### Response and selectivity

**Fig. 1:**
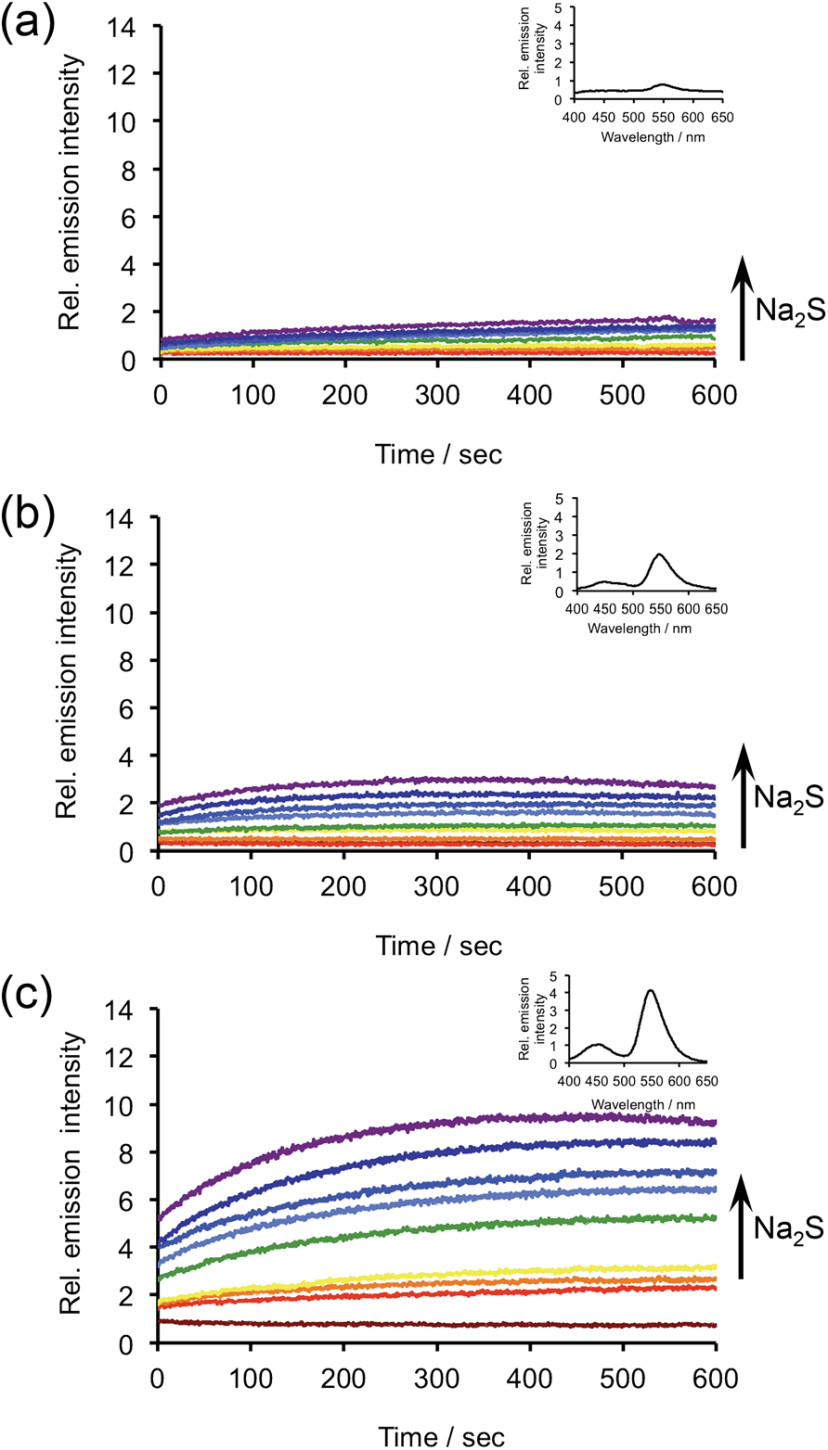
Time scans of the chemiluminescent emission at 545 nm from (a) 40 μM **CHS-1**, (b) 40 μM **CHS-2**, or (c) 40 μM **CHS-3** and 0, 5, 10, 20, 40, 80, 100, 150, 200 μM Na_2_S in 20 mM HEPES buffer (pH 7.4) containing 20% Emerald II Enhancer. Insets are chemiluminescence spectra of (a) 40 μM **CHS-1**, (b) 40 μM **CHS-2**, and (c) 40 μM **CHS-3** and 200 μM Na_2_S, acquired immediately after adding probes.

With the first three **CHS** probes in hand, we proceeded to measure their luminescent responses to H_2_S using an F-7000 Hitachi spectrophotometer. At pH 7.4, treatment of the **CHS** probes with H_2_S resulted in instantaneous luminescent emission that increased over a course of 10 minutes in a dose-dependent manner (Fig. 1). Addition of the fluorescein-based Emerald II Enhancer provided a red-shifted peak centred at 545 nm compared to the 470 nm emission of the phenolate (Fig. 1, insets). Under these physiologically relevant conditions, we observed an increase in the integrated luminescent response to H_2_S from **CHS-1** to **CHS-2** to **CHS-3**, giving 5-fold, 4-fold, and 12-fold turn-on responses respectively. It should be noted that no background corrections were performed and the values in Fig. 1 are direct instrumental values to provide an accurate comparison between probes. At pH 10, **CHS-1** provided the highest chemiluminescence intensity and response, giving a 7-fold increase in photon emission in the first 10 minutes after adding 200 μM H_2_S (Fig. S1†). Under these alkaline conditions, the H_2_S-triggered chemiluminescent emissions of the fluorinated **CHS-2** and chlorinated **CHS-3** were lower than **CHS-1**. The background signal also increases with **CHS-2** and **CHS-3**, reducing the relative increases over control to 2-fold and 3-fold respectively. A key advantage of these probes is that they display an immediate concentration-dependent emission of light that persists for over an hour (Fig. S2†), an ideal property for the high-throughput detection and imaging of H_2_S generated in biological environments.

**Fig. 2:**
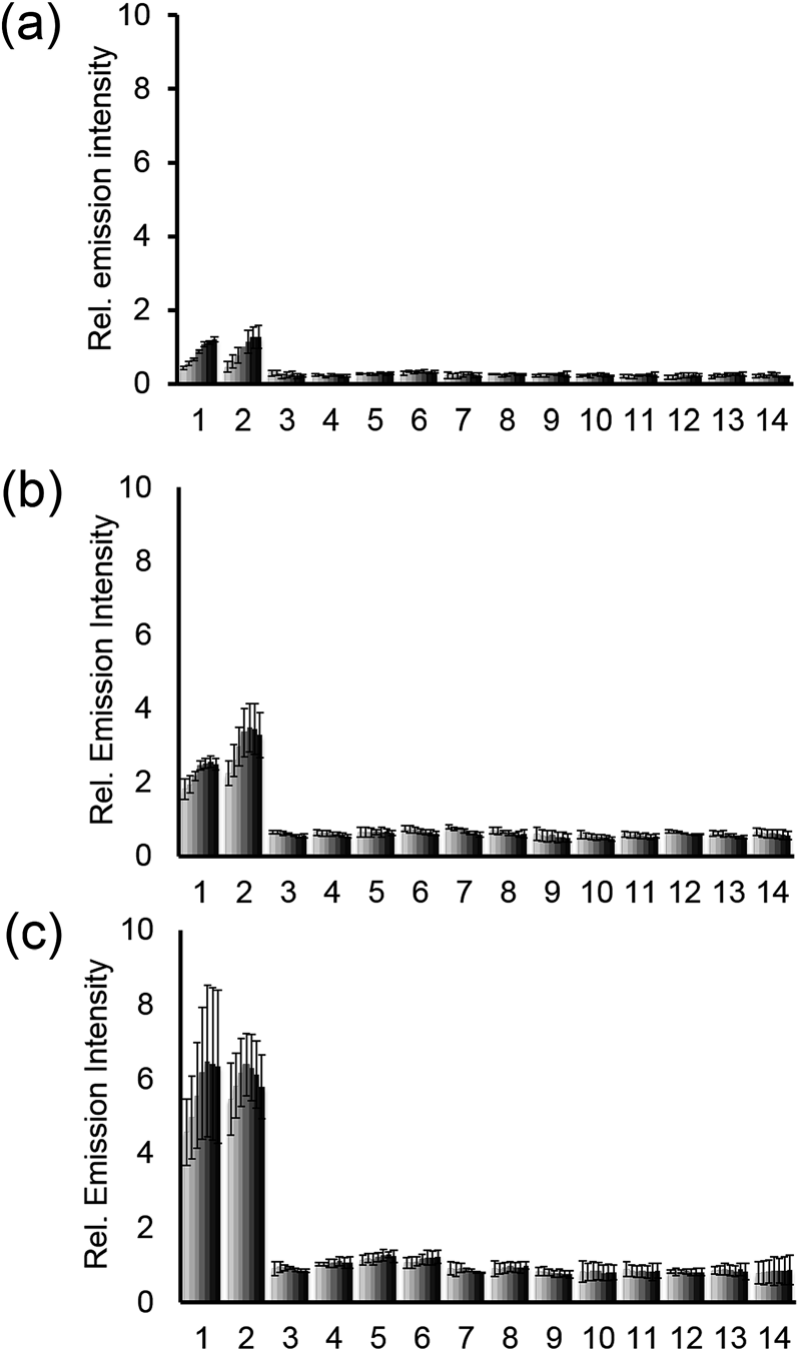
Chemiluminescent responses of (a) 40 μM **CHS-1**, (b) 40 μM **CHS-2**, or (c) 40 μM **CHS-3** to biologically relevant RSON species in 20 mM HEPES buffer (pH 7.4) containing 20% Emerald II Enhancer. Bars represent chemiluminescent emission at 545 nm and 30, 60, 120, 240, 360, 480 and 600 s after addition of RSON species. Data shown are for 5 mM glutathione, 1 mM cysteine and homocysteine, and 200 μM for other RSON species. Legend: (1) Na_2_S; (2) Na_2_S, glutathione, l-cysteine, and homocysteine; (3) glutathione; (4) *S*-nitrosoglutathione; (5) l-cysteine; (6) homocysteine; (7) HNO; (8) NO; (9) NaNO_2_; (10) Na_2_SO_3_; (11) H_2_O_2_; (12) NaClO; (13) ^*t*^BuOOH; (14) blank. Error bars are ± S.D.

We next tested the selectivity of **CHS-1**, **CHS-2**, and **CHS-3** for H_2_S against other biologically relevant reactive sulphur, oxygen, and nitrogen (RSON) species. The response of **CHS-1** to 200 μM Na_2_S in 20 mM HEPES buffered to pH 7.4 was tested against other RSON species by adding 5 mM reduced glutathione (GSH), 1 mM l-cysteine and homocysteine, and 200 μM of *S*-nitrosoglutathione, sulfite (SO_3_^2–^), hydrogen peroxide (H_2_O_2_), hypochlorite (OCl^–^), *tert*-butyl hydroperoxide (^*t*^BuOOH), nitroxyl (HNO), nitric oxide (NO), and nitrite (NO_2_^–^). None of the species tested displayed significant increases in luminescence intensity over the blank control (Fig. 2a). Additionally, the response to Na_2_S was minimally perturbed by the presence of physiological levels of GSH, l-cysteine, and homocysteine. We observed similar results when **CHS-2** and **CHS-3** were evaluated for their selectivity in 20 mM HEPES at pH 7.4 (Fig. 2b and c). These response and selectivity data demonstrate that **CHS-1**, **CHS-2**, and **CHS-3** are able to detect H_2_S at physiologically relevant pH with minimal interference from competing analytes.

### Mechanistic studies of chemiluminescent H_2_S detection

**Fig. 3:**
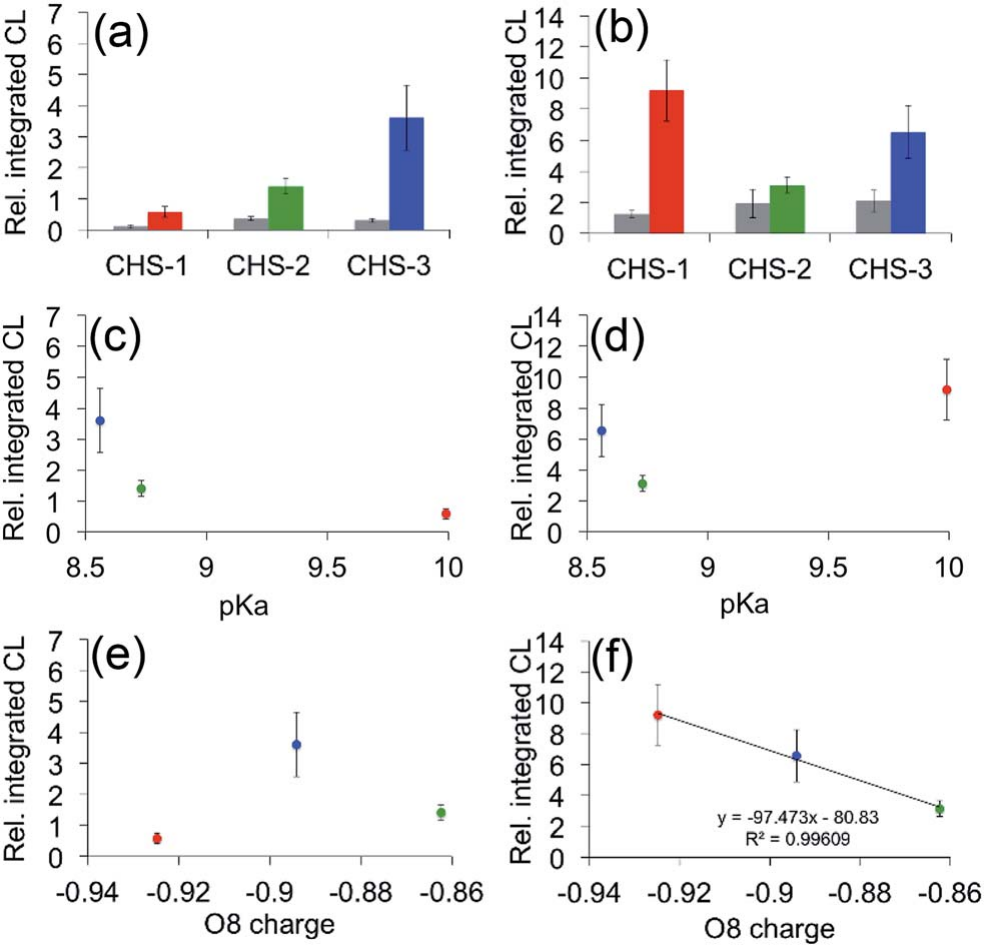
Analysis of chemiluminescent responses. (a and b) Bar graphs for the integrated chemiluminescent emission over 10 min at (a) pH 7.4 or (b) pH 10 of **CHS-1**, **CHS-2**, and **CHS-3** to 0 μM Na_2_S (grey bars) and 200 μM Na_2_S (colored bars). (c and d) Plot of the integrated chemiluminescent emission over 10 min at (c) pH 7.4 or (d) pH 10 of 200 μM Na_2_S and 40 μM **CHS-1** (red), **CHS-2** (green) and **CHS-3** (blue) *versus* experimental p*K*_a_ values of model compounds phenol, 2-fluorophenol, and 2-chlorophenol. (e and f) Plot of the integrated chemiluminescent emission over 10 min at (e) pH 7.4 or (f) pH 10 of 200 μM Na_2_S and 40 μM **CHS-1** (red), **CHS-2** (green) and **CHS-3** (blue) *versus* the calculated atomic charge on the phenolate oxygen (O8). All luminescent measurements were acquired in 20 mM HEPES buffer (pH 7.4) or 100 mM glycine buffer (pH 10) containing 20% Emerald II Enhancer. The reported values are averages of the integrated emission intensities over 10 min (*n* = 4–7). Error bars represent ± S.D. The ESP atomic charges were calculated at the M06/6-311+G(d,p) level of theory using geometries optimized at the B3LYP/6-311+G(d,p) level of theory. Calculations were carried out with the IEF-PCM water solvation model using the Gaussian 09 program package.

We next sought to understand the factors that lead to increased light production and optimized response for 1,2-dioxetane chemiluminescent probes. First, we investigated the role of the phenol/phenolate equilibrium. Since the measurement of the p*K*_a_ of the phenolate dioxetane products of the **CHS** probes is complicated by their rapid chemiluminescent decomposition, we used the reported experimental values of phenol, 2-fluorophenol, and 2-chlorophenol as an approximation.^47^ We plotted the integrated chemiluminescent emission of **CHS-1**, **CHS-2**, and **CHS-3** in response to 200 μM Na_2_S at pH 7.4 and pH 10 (Fig. 3a and b) against the experimental p*K*_a_ values for phenol, 2-fluorophenol, and 2-chlorophenol (Fig. 3c and d). The H_2_S-stimulated emission intensity at pH 7.4 displays an increasing trend with increased phenol acidity, indicating that ionization of the phenol is critical to attaining good luminescent response under these conditions (Fig. 3c). On the other hand, there is no clear correlation with phenol p*K*_a_ and the chemiluminescent response at pH 10 (Fig. 3d). We further investigated the nature of the chemiluminescent emission by performing quantum chemistry calculations on the phenolate structures released from **CHS-1**, **CHS-2**, and **CHS-3** after reaction with Na_2_S (Tables S1–S3†). Geometries were optimized using density functional theory (DFT) at the B3LYP/6-311+G(d,p) level of theory, and the charge on each atom was calculated using an electrostatic potential (ESP) model at the M06/6-311+G(d,p) level of theory. All calculations were carried out with the integral equation formalism polarizable continuum model (IEF-PCM) in water as solvent using the Gaussian 09 program package. While we found no correlation between the charge on the phenolic oxygen (O8) and the chemiluminescent response at pH 7.4 (Fig. 3e), an excellent correlation (*R* ^2^ > 0.99) was observed with the response at pH 10 (Fig. 3f). ESP charge calculations performed with B3LYP and ωB97XD functionals also provided good correlations (*R* ^2^ > 0.97) of the O8 charge and the chemiluminescent response at pH 10 (Fig. S3†). These data indicate that at lower pH, the chemiluminescent emission is governed by the equilibrium between the protonated phenol and unprotonated phenolate oxygen, and are in agreement with previous observations.^48^ **CHS-3** is the most readily ionized and therefore shows the highest chemiluminescent emission. On the other hand, at pH 10 the phenolate species dominates for all three deprotected dioxetanes and the efficiency of chemiluminescent emission is predicted by the more negative O8 charge density, probably due to an increased propensity towards initiation of the first intramolecular electron transfer step of CIEEL.^49,50^ These data reveal trends that, when taken together, provide a powerful predictive model for the design of improved chemiluminescent reagents.

### Using **CHS-3** to detect cellular H_2_S

**Fig. 4:**
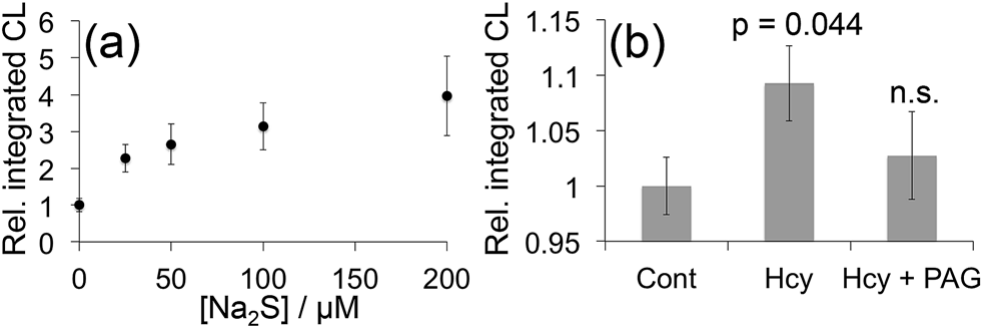
Detection of cellular H_2_S using a multi-well plate reader. (a) Luminescent responses of 40 μM **CHS-3** and 0, 25, 50, 100, and 200 μM Na_2_S in 20 mM HEPES buffer (pH 7.4) containing 20% Emerald II Enhancer. (b) A549 cells were treated with a vehicle control (Cont), 200 μM Hcy (Hcy), or 200 μM Hcy after being pre-treated with 200 μM PAG (Hcy + PAG) for 20 min. 20 min after incubating with Hcy or vehicle, the cells were washed and treated with 40 μM **CHS-3** and 125 μL Emerald II Enhancer. The reported values represent the average luminescent intensity of replicate experiments (*n* = 12, *p* = 0.044). Error bars represent ± S.E.M.

Our results show that **CHS-3** provides the most robust light emission at physiological pH when compared to **CHS-1** and **CHS-2**. We therefore examined the ability of **CHS-3** to detect cellular H_2_S production in human lung adenocarcinoma epithelial cells (A549). These cells express the enzyme cystathionine γ-lyase (CSE),^51^ which can utilize homocysteine (Hcy) as a substrate for H_2_S production. We first demonstrated the ability of **CHS-3** to detect exogenous Na_2_S in a multi-well plate reader format (Fig. 4a), and determined an estimated detection limit (blank control + 3 S.D.) of 5.4 μM. We then incubated A549 cells with Hcy, a substrate for the enzyme CSE. This resulted in ∼10% increase (*n* = 12, *p* = 0.044) in luminescent emission from **CHS-3** compared with vehicle treated cells (Fig. 4b). Pre-incubation with the CSE inhibitor d,l-propargylglycine (PAG) before adding Hcy attenuates signal observed from **CHS-3**. Although the observed increase is small, it is statistically significant and the efficacy of **CHS-3** for endpoint detection of H_2_S generated by whole cells at physiological pH is an important advance that sets the stage for imaging H_2_S in living animals.

### *In vivo* imaging of H_2_S using **CHS-3**

**Fig. 5:**
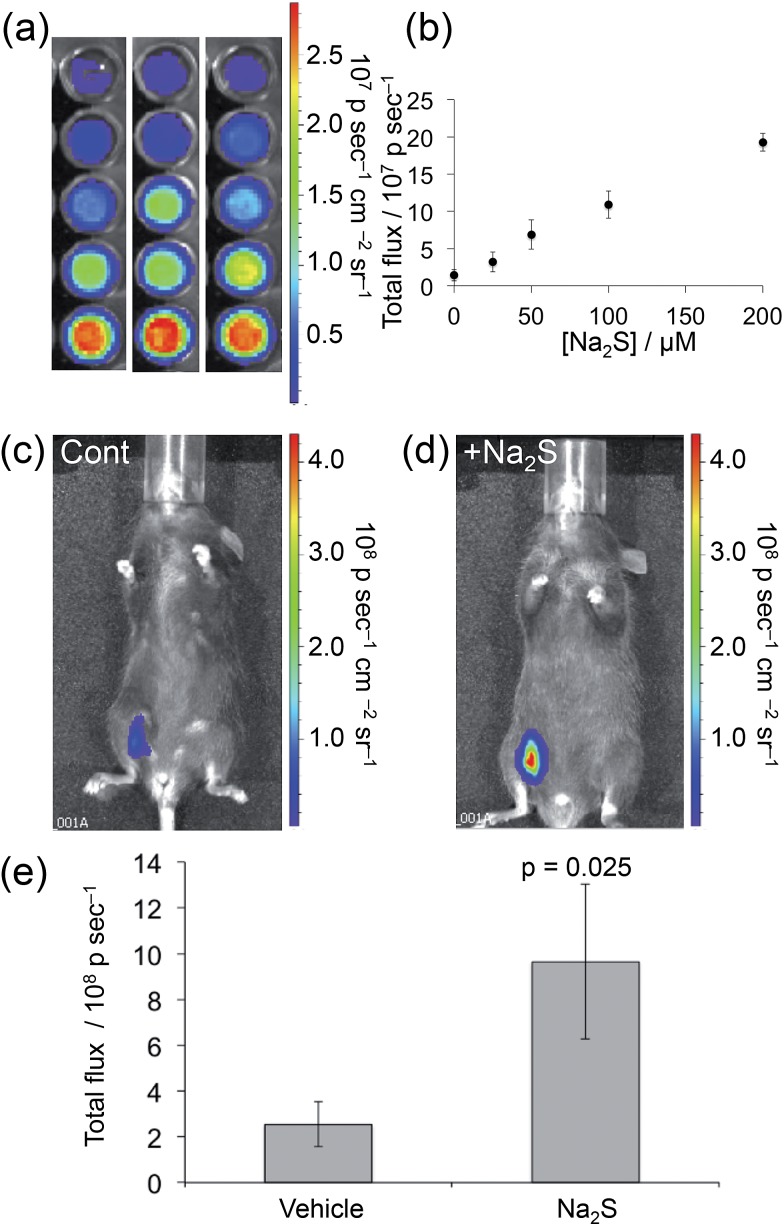
Imaging H_2_S using **CHS-3**. (a) Images 30 s after adding 40 μM **CHS-3** to 0, 25, 50, 100, and 200 μM Na_2_S in 20 mM HEPES buffer (pH 7.4) containing 20% Emerald II Enhancer (*n* = 3). (b) Plot of total photon flux *versus* H_2_S concentration for the experiments described in (a). (c and d) Images of living C6 brown mice 30 s after administering i.p. injections of 0.08 μmol **CHS-3** and (c) vehicle control or (d) 0.4 μmol Na_2_S in 100 μL 20 mM HEPES buffer (pH 7.4) containing 20% Emerald II Enhancer. (e) Quantification of the total photon flux from three replicates of the experiments described in (c) and (d). Statistical analyses were performed with a two-tailed Student's *t*-test (*n* = 3, *p* = 0.025). Error bars are ± S.D.

We next investigated the ability of **CHS-3** to image H_2_S at physiological pH using an IVIS Spectrum. An opaque 96-well plate was loaded with 0, 25, 50, 100, and 200 μM Na_2_S in 20 mM HEPES buffer (pH 7.4) containing 20% Emerald II Enhancer. **CHS-3** was added at 40 μM and imaged to reveal a clear increase in luminescence intensity with increasing H_2_S concentrations (Fig. 5a). Averages of repeated experiments provide a good linear response in the range of 0–200 μM when imaged 30 s after exposure to H_2_S (Fig. 5b). We next applied a mouse carcass model to determine if **CHS-3** would have sufficient light output to be observable through mammalian tissue. The carcasses of sacrificed SCID/BALB-C mice were injected with **CHS-3** and either a vehicle control (Fig. S4a and b†) or 0.4 μmol H_2_S (Fig. S4c and d†) into the peritoneal cavity. An increase in luminescence could be clearly observed in the peritoneum of the carcass injected with H_2_S *versus* the carcass injected with the vehicle control.

Confident that light emission from **CHS-3** was capable of significant tissue penetration, we finally sought to establish the ability of **CHS-3** to image H_2_S in living animals. C6 brown mice were administered i.p. injections on one side of their peritoneal cavity. The skin was raised during injections to avoid puncturing internal organs. Images were acquired 30 s after injecting 0.08 μmol **CHS-3** and either 0.4 μmol Na_2_S or a vehicle control (H_2_O) in 100 μL HEPES buffered at pH 7.4 containing 20% Emerald II Enhancer. The final concentration of Na_2_S in the injection was 4 mM. While vehicle control experiments produced modest signal (Fig. 5c), the mice that received Na_2_S treatments displayed robust emission of light that was easily detected through their tissue (Fig. 5d). Quantification of the total photon flux from replicate experiments (Fig. S5†) revealed ∼4-fold increase (*n* = 3, *p* = 0.025) in the luminescence response in the Na_2_S treated mice *versus* vehicle controls (Fig. 5e). The agents were well tolerated and the mice showed no immediate outward signs of malaise. Taken together, these data provide a key milestone towards the development of a new class of *in vivo* imaging tools for investigating biological hydrogen sulphide.

## Conclusions

We have designed and synthesized three 1,2-dioxetane chemiluminescent reaction-based H_2_S probes, **CHS-1**, **CHS-2**, and **CHS-3** that display immediate light emission upon reacting with H_2_S at physiological pH, a significant advance for H_2_S detection technology. These reagents provide a sensitive and selective detection platform for H_2_S using spectrophotometers, multi-well plate readers, and IVIS Spectrum instruments. We have provided a computational and experimentally supported mechanistic framework that serves as a predictive model for designing sterically hindered 1,2-dioxetanes with improved light emission based on the p*K*_a_ of the phenol released after reacting with H_2_S and the relative atomic charge densities of these structures. Finally, we demonstrated that **CHS-3** was not only capable of detecting cellular H_2_S produced by A549 cells treated with homocysteine at neutral pH, but also has the rare ability to image H_2_S in living animals. While **CHS-3** provides an exciting proof of principle for *in vivo* chemiluminescence imaging, we are currently engaged in improving the sensitivity and biocompatibility of chemiluminescent 1,2-dioxetane reagents for diverse applications, and anticipate that these new optimized systems will provide powerful whole animal imaging tools across a range of biological analytes and parameters.
