# Supplementary material for: Chemiluminescent probes for imaging H2S in living animals
Source: Chem Sci. 2015 Jan 2;6(3):1979–85. doi: 10.1039/c4sc03516j (PMC4335805; doi:10.1039/c4sc03516j)
Supplement: Supplementary file 1 [file SC-006-C4SC03516J-s001.pdf]

Electronic Supplementary Material (ESI) for Chemical Science.
This journal is © The Royal Society of Chemistry 2015

Supporting Information:

Chemiluminescent Probes for Imaging H_2S in Living Animals

J. Cao,[a,b] R. Lopez,[c] J. M. Thacker, [a] J. Y. Moon, [a] C. Jiang,[d] S. N. S. Morris, [e] J. H. Bauer, [b,e] P. Tao, R. P. Mason, and A. R. Lippert*[a,b]

[a] Department of Chemistry, Southern Methodist University, Dallas, TX 75275-0314, USA.

[b] Center for Drug Discovery, Design, and Delivery (CD4), Southern Methodist University, Dallas, TX 75275-0314, USA

[c] Laboratory of Prognostic Radiology, Pre-clinical Imaging Section, Department of Radiology, UT Southwestern Medical Center, Dallas, TX 75390-9058, USA

[d] Hockaday School, Dallas, TX 75229, USA

[e] Department of Biological Sciences, Southern Methodist University, Dallas, TX 75275-0314, USA

Table of contents **Pages**

1. Synthetic procedures S2

2. Chemiluminescent response S10

3. Selectivity tests S11

4. Computational results S13

5. Cellular experiments S17

6. Imaging experiments S18

7. ^{1}H and ^{13}C NMR spectra S20

8. References S51

1. Synthetic procedures

General materials and methods. All reactions were performed in dried glassware under an atmosphere of dry N_2. Silica gel P60 (SiliCycle) was used for column chromatography and SiliCycle 60 F254 silica gel (precoated sheets, 0.25 mm thick) was used for analytical thin layer chromatography. Plates were visualized by fluorescence quenching under UV light or by staining with iodine. Other reagents were purchased from Sigma-Aldrich (St. Louis, MO), Alfa Aesar (Ward Hill, MA), EMD Millipore (Billerica, MA), Oakwood Chemical (West Columbia, SC), and Cayman Chemical (Ann Arbor, MI) and used without further purification. ^{1}H NMR and ^{13}C NMR spectra for characterization of new compounds and monitoring reactions were collected in $CDCl_3$ (Cambridge Isotope Laboratories, Cambridge, MA) on a JEOL 500 MHz spectrometer in the Department of Chemistry at Southern Methodist University. All chemical shifts are reported in the standard notation of parts per million using the peak of residual proton signals of the deuterated solvent as an internal reference. Coupling constant units are in Hertz (Hz) Splitting patterns are indicated as follows: br, broad; s, singlet; d, doublet; t, triplet; q, quartet; m, multiplet; dd, doublet of doublets; dt, doublet of triplets. High resolution mass spectroscopy was performed on a Shimadzu IT-TOF (ESI source) and low resolution mass spectroscopy was performed on a Shimadzu LCMS-8050 Triple Quadrupole LCMS (ESI source) or a Shimadzu Matrix Assisted Laser Desorption/Ionization MS (MALDI) at the Shimadzu Center for Advanced Analytical Chemistry at the University of Texas, Arlington.

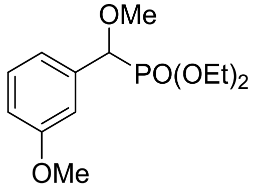

3a

Diethyl (methoxy (3-methoxyphenyl) methyl) phosphonate (3a). 3-Methoxybenzaldehyde (1.83 mL, 15.0 mmol, 1.0 equiv), trimethyl orthoformate (1.65 mL, 15.0 mmol, 1.0 equiv) and *p*-toluenesulfonic acid (258 mg, 1.50 mmol, 0.10 equiv) were added to a dry round-bottom flask and flushed with N_2. The reaction contents were dissolved in 6.0 mL of MeOH. The reaction proceeded for 24 h at rt, and was then neutralized with NEt_3. After neutralization, the crude mixture was poured into 30 mL saturated aq $NaHCO_3$. The layers were separated and the aqueous layer was washed with an additional 2 x 40 mL EtOAc. The combined organic layers were collected and dried over Na_2SO_4, filtered, and concentrated to yield the crude acetal **2a** (2.52 g). Compound **2a** (1.94 g, 10.7 mmol, 1.0 equiv) was dissolved in 10.0 mL CH_2Cl_2, and triethyl phosphite (1.89 mL, 11.0 mmol, 1.03 equiv) and boron trifluoride etherate (1.38 mL, 11.0 mmol, 1.03 equiv) were added dropwise at 0 °C. Once all the reagents were added, the reaction was heated to 30 °C and allowed to react for 1 h under N_2 atmosphere. The reaction was quenched with 20 mL saturated aq $NaHCO_3$, extracted with 2 x 30 mL EtOAc, and evaporated under reduced pressure. Purification by column chromatography (40%–100% EtOAc/hexanes)

afforded **3a** as a pale yellow oil (2.332 g, 89%). ^{1}H NMR (500 MHz, $CDCl_3$) δ 7.26 (t, 1H, *J* = 8.0 Hz), 6.97–7.02 (m, 2H), 6.85 (d, 1H, *J* = 8.0 Hz), 4.47 (d, 1H, *J* = 15.5 Hz), 3.90–4.13 (m, 4H), 3.81 (s, 3H), 3.38 (s, 3H), 1.20–1.29 (m, 6H); ^{13}C NMR (125 MHz, $CDCl_3$) δ 159.63, 135.94, 129.31, 120.43, 114.31, 112.96, 80.52, 79.71, 63.10 (d, *J* = 7.2 Hz), 62.93 (d, *J* = 7.2 Hz), 58.76 (d, *J* = 14.3 Hz), 55.22, 16.40 (d, *J* = 5.9 Hz), 16.33 (d, *J* = 5.9 Hz); HRMS calcd for $C_{13}H_{21}O_5P$ ($M+Na^+$) 311.1019, found 311.1013.

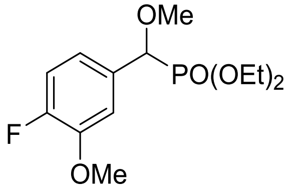

3b

Diethyl ((4-fluoro-3-methoxyphenyl) (methoxy) methyl) phosphonate (3b). 4-Fluoro-3-methoxybenzaldehyde (1.0 g, 6.5 mmol, 1.0 equiv), trimethyl orthoformate (0.71 mL, 6.5 mmol, 1.0 equiv) and *p*-toluenesulfonic acid (111.9 mg 0.6498 mmol, 0.10 equiv) were dissolved in 4.0 mL of MeOH. The reaction proceeded for 24 h at rt, and was then neutralized with NEt_3. After neutralization, the crude mixture was poured into 30 mL saturated aq $NaHCO_3$. The layers were separated and the aqueous layer was washed with an additional 2 x 40 mL EtOAc, the combined organic layers were collected and dried over Na_2SO_4, filtered, and concentrated to yield crude acetal **2b** (1.25 g). Compound **2b** (1.25 g, 6.25 mmol, 1.0 equiv) was dissolved in 6.0 mL CH_2Cl_2, and triethyl phosphite (1.10 mL, 6.44 mmol, 1.0 equiv) and boron trifluoride etherate (0.80 mL, 6.4 mmol, 1.0 equiv) were added dropwise at 0 °C. Once all the reagents were added, the reaction was heated to 30 °C and allowed to react for 1 h under N_2 atmosphere. The reaction was quenched with 20 mL saturated aq $NaHCO_3$, extracted with 2 x 30 mL EtOAc, and evaporated under reduced pressure. Purification by column chromatography (40%–100% EtOAc/hexanes) afforded **3b** as a yellow oil (1.247 g, 66%). ^{1}H NMR (500 MHz, $CDCl_3$) δ 6.92 (dt, 1H, *J* = 8.6, 2.3 Hz), 6.83 (dd, 1H, *J* = 10.9, 2.3 Hz), 6.71–6.75 (m, 1H), 4.26 (d, 1H, *J* = 15.5 Hz), 3.80–3.91 (m, 4H), 3.68 (s, 3H), 3.17 (s, 3H), 0.95–1.10 (m, 6H); ^{13}C NMR (125 MHz, $CDCl_3$) 152.58, 150.65, 147.02 (d, *J* = 10.7 Hz), 130.20, 119.90 (d, *J* = 7.2 Hz), 114.95 (d, *J* = 19.1 Hz), 112.11, 79.84, 78.49, 62.41 (d, *J* = 7.2 Hz), 62.18 (d, *J* = 7.2 Hz), 58.32 (d, *J* = 14.3 Hz), 55.36, 15.70 (d, *J* = 5.9 Hz), 15.64 (d, *J* = 5.9 Hz); HRMS calcd for $C_{13}H_{20}FO_5P$ ($M+Na^+$) 329.0925, found 329.0931.

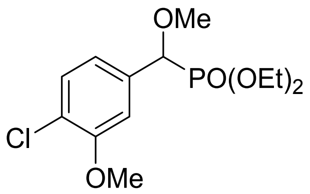

3c

Diethyl ((4-chloro-3-methoxyphenyl) (methoxy) methyl) phosphonate (3c). 4-Chloro-3-methoxybenzaldehyde (400 mg, 2.34 mmol, 1.0 equiv), trimethyl orthoformate (256 µL, 2.34 mmol, 1.0 equiv) and *p*-toluenesulfonic acid (40.4 mg 0.234 mmol, 0.10 equiv) were added to a dry flask and flushed with N_2. The reaction contents were dissolved in 1.5 mL of MeOH. The reaction proceeded for 24 h at rt, and was then neutralized with NEt_3. After neutralization, the crude mixture was poured into 20 mL saturated aq $NaHCO_3$. The layers were separated and the aqueous layer was washed with an additional 2 x 30 mL EtOAc, the combined organic layers were collected and dried over Na_2SO_4, filtered, and concentrated to yield crude acetal **2c** (474 mg). Compound **2c** (474 mg, 2.19 mmol, 1.0 equiv) was dissolved in 2.0 mL CH_2Cl_2, and triethyl phosphite (0.39 mg, 2.3 mmol, 1.0 equiv) and boron trifluoride etherate (0.28 mL, 2.3 mmol, 1.0 equiv) were added dropwise at 0 °C. Once reagents were added, the reaction was heated to 30 °C and allowed to react for 1 h under N_2 atmosphere. The reaction was quenched with 20 mL saturated aq $NaHCO_3$, extracted with 2 x 30 mL EtOAc, and evaporated under reduced pressure. Purification by column chromatography (40%–100% EtOAc/hexanes) afforded **3c** as a pale yellow oil (598 mg, 83%). ^{1}H NMR (500 MHz, $CDCl_3$) δ 7.23 (d, 1H, *J* = 8.1 Hz), 6.98 (t, 1H, *J* = 2.0 Hz), 6.83 (dt, 1H, *J* = 6.3, 2.0 Hz), 4.37 (d, 1H, *J* = 16.1 Hz), 3.86–4.00 (m, 4H), 3.81 (s, 3H), 3.29 (s, 3H), 1.11–1.26 (m, 6H); ^{13}C NMR (125 MHz, $CDCl_3$) δ 154.82, 134.40, 129.72, 122.29, 120.74, 111.12, 80.44, 79.43, 63.05 (d, *J* = 10.7 Hz), 62.81 (d, *J* = 10.7 Hz), 58.65 (d, *J* = 14.3 Hz), 55.97, 16.26 (d, *J* = 5.9 Hz), 16.18 (d, *J* = 5.9 Hz); HRMS calcd for $C_{13}H_{20}ClO_5P$ (M+Na$^+$) 345.0629, found 345.0624.

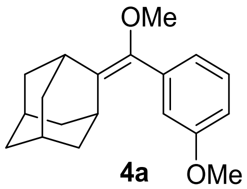

4a

(1*r*, 3*r*, 5*R*, 7*S*) - 2 - (methoxy (3-methoxyphenyl) methylene) adamantane (4a). Compound **3a** (1.27 g, 4.47 mmol, 1.0 equiv) and 2-adamantanone (939 mg, 6.25 mmol, 1.4 equiv) were dissolved separately in 15 mL and 8 mL anhydrous THF under N_2 atmosphere and cooled to –78 °C by mixing dry ice with acetone. n-Butyl lithium (3.91 mL, 6.25 mmol, 1.4 equiv) was then added dropwise to the solution of compound **3a** at –78 °C to form the phosphonate carbanion. After 5 min, the 2-adamantanone solution was slowly added. The reaction was slowly warmed to rt and heated to 35 °C for 2 h and then refluxed for 1 h. After the reaction mixture was cooled to rt, it was quenched with 20 mL saturated aq NH_4Cl. The mixture was then extracted with 2 x 30 mL EtOAc and evaporated under reduced pressure. Purification by column chromatography (1:20 EtOAc/hexanes) delivered **4a** as a colorless oil (1.2482 g, 85%). ^{1}H NMR (500 MHz, $CDCl_3$) δ 7.25 (t, 1H, *J* = 8.0 Hz), 6.89–6.92 (m, 2H), 6.83 (dd, 1H, *J* = 8.0, 3.0 Hz), 3.81 (s, 3H), 3.31 (s, 3H), 3.27 (s, 1H), 2.66 (s, 1H), 1.71–1.98 (m, 12H); ^{13}C NMR (125 MHz, $CDCl_3$) δ 159.45, 143.45, 136.95, 131.74, 128.96, 122.05, 114.74, 113.07, 57.82, 55.27, 39.30, 39.16, 37.29, 32.34, 30.29, 28.42; HRMS calcd for $C_{19}H_{24}O_2$ (M+H$^+$) 285.1849, found 285.1853.

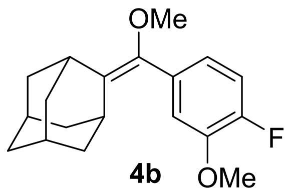

4b

(1*r*, 3*r*, 5*R*, 7*S*) - 2 - ((4-fluoro-3-methoxyphenyl) (methoxy) methylene) adamantane (4b). Compound **3b** (1.17 g, 3.87 mmol, 1.0 equiv) and 2-adamantanone (730 mg, 4.86 mmol, 1.3 equiv) were dissolved separately in 13 mL and 6 mL anhydrous THF under N_2 atmosphere and cooled to –78 °C by mixing dry ice and acetone. n-Butyl lithium (3.0 mL, 4.86 mmol, 1.3 equiv) was then added dropwise to the solution of compound **3b** at –78 °C to form the phosphonate carbanion. After 5 min, the 2-adamantanone solution was slowly added. The reaction was slowly warmed to rt and heated to 35 °C for 2 h and then refluxed for 1 h. After the reaction mixture was cooled to rt, the reaction mixture was quenched with 20 mL saturated aq NH_4Cl. The mixture was then extracted with 2 x 30 mL EtOAc and evaporated under reduced pressure. Purification by column chromatography (1:20 EtOAc/hexanes) delivered **4b** as a colorless oil (1.12 g, 85%). ^{1}H NMR (500 MHz, $CDCl_3$) δ 6.99–7.03 (m, 1H), 6.94 (dd, 1H, *J* = 8.0, 2.0 Hz), 6.79–6.82 (m, 1H), 3.87 (s, 3H), 3.29 (s, 3H), 3.23 (s, 1H), 2.60 (s, 1H), 1.58–1.97 (m, 12H); ^{13}C NMR (125 MHz, $CDCl_3$) δ 152.58, 150.62, 147.18 (d, *J* = 10.7 Hz), 142.81, 131.93, 122.12 (d, *J* = 7.2 Hz), 115.40 (d, *J* = 19.1 Hz), 114.22, 57.79, 56.26, 39.24, 39.11, 37.23, 32.38, 30.30, 28.36; HRMS calcd for $C_{19}H_{23}ClO_2$ (M+H$^+$) 303.1755, found 303.1762.

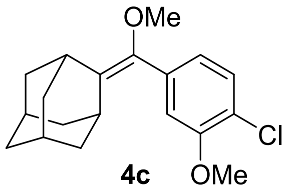

4c

(1*r*, 3*r*, 5*R*, 7*S*) - 2 - ((4-chloro-3-methoxyphenyl) (methoxy) methylene) adamantane (4c). Compound **3c** (365 mg, 1.13 mmol, 1.0 equiv) and 2-adamantanone (215 mg, 1.43 mmol, 1.3 equiv) were dissolved separately in 4 mL and 2 mL anhydrous THF under N_2 atmosphere and cooled to –78 °C by mixing dry ice and acetone. n-Butyl lithium (0.89 mL, 1.4 mmol, 1.3 equiv) was then added dropwise to the solution of compound **3c** at –78 °C to form the phosphonate carbanion. After 5 min reaction, the 2-adamantanone solution was slowly added. The reaction was slowly warmed to rt and heated to 35 °C for 2 h and then refluxed for 1 h. After the reaction mixture was cooled to rt, it was quenched with 10 mL saturated aq NH_4Cl. The mixture was then extracted with 2 x 20 mL EtOAc and evaporated under reduced pressure. Purification by column chromatography (1:20 EtOAc/hexanes) delivered **4c** as a colorless oil (294 mg, 78%). ^{1}H NMR (500 MHz, $CDCl_3$) δ 7.30 (d, 1H, *J* = 8.0 Hz), 6.91 (d, 1H, *J* = 1.8 Hz), 6.83 (dd, 1H, *J* = 8.0, 1.8 Hz), 3.89 (s, 3H), 3.30 (s, 3H), 3.24 (s, 1H), 2.62 (s, 1H), 1.58–1.97 (m, 12H); ^{13}C NMR (125 MHz, $CDCl_3$) δ 154.84, 142.77, 135.44, 132.56, 129.62, 122.54, 121.41, 112.82, 57.90, 56.16,

39.24, 39.11, 37.21, 32.43, 30.35, 28.34; HRMS calcd for $C_{19}H_{23}ClO_2$ (M+H$^+$) 319.1459, found 319.1461.

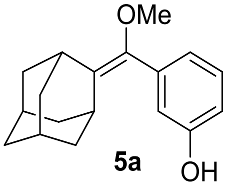

5a

3-(((1*r*, 3*r*, 5*R*, 7*S*)-adamantan-2-ylidene) (methoxy) methyl) phenol (5a). Sodium ethane thiolate (769 g, 9.14 mmol, 2.5 equiv) and cesium carbonate (2.98 g, 9.14 mmol, 2.5 equiv) were added to a dry round bottle flask containing compound **4a** (1.04 g, 3.66 mmol, 1.0 equiv) dissolved in 30 mL anhydrous DMF under N_2 atmosphere. After refluxing overnight, the reaction mixture was partitioned between EtOAc and NH_4Cl, dried over Na_2SO_4 and evaporated under high vacuum to remove residual DMF. Purification by column chromatography (1:15–1:10 EtOAc/hexanes) afforded **5a** as an white solid (620.9 mg, 63%). ^{1}H NMR (500 MHz, $CDCl_3$) δ 7.17–7.21 (m, 1H), 6.99 (s, 1H), 6.80–6.94 (m, 3H), 3.35 (s, 3H), 3.23 (s, 1H), 2.67 (s, 1H), 1.63–1.98 (m, 12H); ^{13}C NMR (125 MHz, $CDCl_3$) δ 155.92, 142.62, 136.48, 132.63, 129.11, 121.70, 115.86, 114.71, 57.73, 39.06, 38.93, 37.03, 32.20, 30.26, 28.16. LRMS (MALDI) calcd for $C_{18}H_{22}O_4$ (M$^+$) 270.1620, found 270.1815.

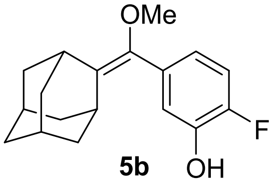

5b

5-(((1*r*, 3*r*, 5*R*, 7*S*)-adamantan-2-ylidene) (methoxy) methyl)-2-fluorophenol (5b). Sodium ethane thiolate (215 mg, 2.56 mmol, 1.5 equiv) and cesium carbonate (834 g, 2.56 mmol, 1.5 equiv) were added to a dry round bottle flask filled with N_2 containing compound **4b** (516 mg, 1.71 mmol, 1.0 equiv) dissolved in 10 mL of anhydrous DMF. After refluxing overnight, the reaction mixture was partitioned between EtOAc and NH_4Cl, dried over Na_2SO_4 and evaporated under high vacuum to remove residual DMF. Purification by column chromatography (1:15 EtOAc/hexanes) afforded **5b** as an white solid (323 mg, 62%). ^{1}H NMR (500 MHz, $CDCl_3$) δ 7.00–7.05 (m, 1H), 6.95–6.98 (m, 1H), 6.78–6.82 (m, 1H), 6.74 (m, 1H), 5.47 (s, 1H), 3.30 (s, 3H), 3.23 (s, 1H), 2.59 (s, 1H), 1.66–1.96 (m, 12H); ^{13}C NMR (125 MHz, $CDCl_3$) δ 156.56, 142.77, 138.58, 135.43, 132.64, 121.73, 117.53, 115.30, 57.89, 39.16, 39.02, 37.10, 32.18, 30.78, 28.23; HRMS calcd for $C_{18}H_{21}FO_2$ (M–H$^+$) 287.1453, found 287.1456.

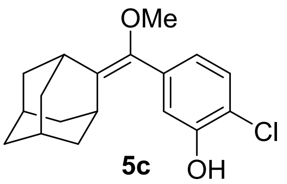

5c

5-(((1*r*, 3*r*, 5*R*, 7*S*)-adamantan-2-ylidene) (methoxy) methyl)-2-chlorophenol (5c). Sodium ethane thiolate (204 mg, 2.43 mmol, 2.5 equiv) and cesium carbonate (791 mg, 2.43 mmol, 2.5 equiv) were added to a dry round bottle flask containing compound **4c** (309 mg, 0.971 mmol, 1.0 equiv) dissolved in 9.0 mL anhydrous DMF under N_2 atmosphere. After refluxing overnight, the reaction mixture was partitioned between EtOAc and NH_4Cl, dried over Na_2SO_4 and evaporated under high vacuum to remove residual DMF. Purification by column chromatography (1:12 EtOAc/hexanes) afforded **5c** as an white solid (232 mg, 79%). ^{1}H NMR (500 MHz, $CDCl_3$) δ 7.28 (d, 1H, J = 8.0 Hz), 6.98 (d, 1H, J = 3.5 Hz), 6.83 (dd, 1H, J = 8.0, 3.5 Hz) 5.95 (s, 1H), 3.30 (s, 3H), 3.22 (s, 1H), 2.62 (s, 1H), 1.55–1.96 (m, 12H); ^{13}C NMR (125 MHz, $CDCl_3$) δ 151.12, 142.18, 135.88, 132.79, 128.57, 122.38, 118.84, 116.97, 57.80, 39.08, 38.94, 37.04, 32.22, 30.21, 28.17; HRMS calcd for $C_{18}H_{21}ClO_2$ (M–H$^+$) 303.1157, found 303.1150.

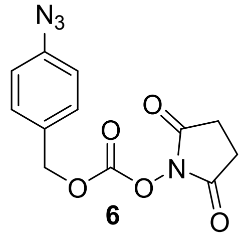

6

4-azidobenzyl (2,5-dioxopyrrolidin-1-yl) carbonate (6). *N,N*'-Disuccinimidyl carbonate (830 mg, 3.25 mmol, 1.5 equiv) was added to a solution of 4-azidobenzyl alcohol (323 mg, 2.16 mmol, 1.0 equiv) in 5.0 mL CH_2Cl_2, followed directly by the addition of NEt_3 (0.91 mL, 6.5 mmol, 3.0 equiv). The reaction was stirred for 4 h at rt. The reaction was quenched with 20 mL 1 M $NaHCO_3$, extracted with 2 x 30 mL EtOAc, washed with 10 mL brine, dried over Na_2SO_4, filtered, and concentrated to yield **6** (628.7 mg) as an orange oil and used without further purification. ^{1}H NMR (500 MHz, $CDCl_3$) δ 7.38 (d, 2H, J = 8.6 Hz), 7.03 (d, 2H, J = 8.6 Hz), 5.29 (s, 2H), 2.82 (s, 4H).

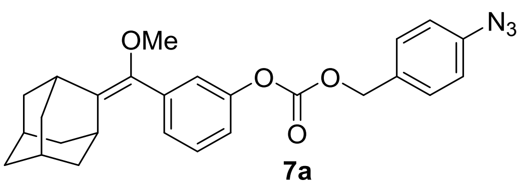

7a

3-(((1*r*, 3*r*, 5*R*, 7*S*)-adamantan-2-ylidene) (methoxy) methyl)phenyl (4-azidobenzyl) carbonate (7a). Compound **5a** (77 mg, 0.29 mmol, 1.0 equiv) was dissolved in 1.5 mL 4:1 THF:CH_2Cl_2 in a dry flask under N_2 atmosphere. Compound **6** (126 mg, 0.44 mmol, 1.5 equiv)

was added as a solution in 1.5 mL CH_2Cl_2. DMAP (53 mg, 0.44 mmol, 1.5 equiv) and NEt_3 (124 µL, 0.899 mmol, 3.1 equiv) were then added in succession. After 14 h of stirring at rt, the mixture was poured into a separatory funnel containing 20 mL CH_2Cl_2 and 15 mL DI-H_2O and extracted with 3 x 20 mL CH_2Cl_2. The organic layer was washed with 10 mL brine, dried over Na_2SO_4, filtered, and concentrated. Purification by silica column chromatography (1:15 EtOAc/hexanes) afforded **7a** as a clear oil (84 mg, 62%). ^{1}H NMR (500 MHz, $CDCl_3$) δ 7.43 (d, 2H, J = 8.6 Hz), 7.34 (t, 1H, J = 7.5 Hz), 7.18–7.20 (m, 1H), 7.06–7.12 (m, 2H), 7.04 (d, 2H, J = 8.6 Hz), 5.23 (s, 2H), 3.29 (s, 3H), 3.24 (s, 1H), 2.64 (s, 1H), 1.65–2.10 (m, 12H); ^{13}C NMR (125 MHz, $CDCl_3$) δ 153.51, 150.84, 142.52, 140.61, 137.13, 132.78, 131.36, 130.30, 128.94, 126.97, 121.68, 119.82, 119.23, 69.66, 57.90, 39.11, 38.99, 37.07, 32.09, 30.22, 28.19; HRMS calcd for $C_{26}H_{27}N_3O_4$ (M+H$^+$) 446.2074, found 446.2073.

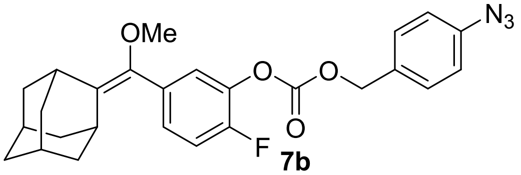

7b

5-(((1*r*, 3*r*, 5*R*, 7*S*)-adamantan-2-ylidene) (methoxy) methyl)-2-fluorophenyl (4-azidobenzyl) carbonate (7b). Compound **5b** (123 mg, 0.427 mmol, 1.0 equiv) was dissolved in 3.5 mL 4:1 THF:CH_2Cl_2 in a dry flask under N_2 atmosphere. Compound **6** (186 mg, 0.641 mmol, 1.5 equiv) was added as a solution in 3.3 mL CH_2Cl_2, followed directly by the addition of DMAP (78.3 mg, 0.641 mmol, 1.5 equiv) and NEt_3 (185 µL, 1.32 mmol, 3.1 equiv). After 14 h of stirring at rt, the mixture was poured into a separatory funnel containing 30 mL CH_2Cl_2 and 20 mL DI-H_2O and extracted with 3 x 30 mL CH_2Cl_2. The organic layer was washed with 10 mL brine, dried over Na_2SO_4, filtered, and concentrated. Purification by silica column chromatography (1:15 EtOAc/hexanes) afforded **7b** as a clear oil (125 mg, 63%). ^{1}H NMR (500 MHz, $CDCl_3$) δ 7.43 (d, 2H, J = 8.0 Hz), 7.10–7.20 (m, 3H), 7.05 (d, 2H, J = 8.0 Hz), 5.25 (s, 2H), 3.28 (s, 3H), 3.22 (s, 1H), 2.60 (s, 1H), 1.53–2.10 (m, 12H); ^{13}C NMR (125 MHz, $CDCl_3$) δ 153.98, 152.66, 141.64, 140.69, 132.98, 132.29, 131.12, 130.25, 128.16, 123.89, 119.25, 116.38, 116.24, 70.18, 57.90, 39.08, 38.96, 37.02, 32.11, 30.24, 28.14. LRMS (MALDI) calcd for $C_{26}H_{26}ClN_3O_4$ (M$^+$) 463.1907, found 463.3052.

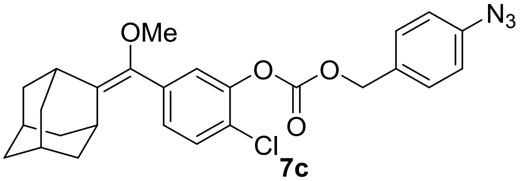

7c

5-(((1*r*, 3*r*, 5*R*, 7*S*)-adamantan-2-ylidene) (methoxy) methyl)-2-chlorophenyl (4-azidobenzyl) carbonate (7c). Compound **5c** (66.6 mg, 0.218 mmol, 1.0 equiv) was dissolved in 1.5 mL 4:1 THF: CH_2Cl_2 in a dry flask under N_2 atmosphere. Compound **6** (76.6 mg, 0.262 mmol, 1.2

equiv) was added as a solution in 1.2 mL CH_2Cl_2. DMAP (40.0 mg, 0.327 mmol, 1.5 equiv) and NEt_3 (95 μL, 0.68 mmol, 3.1 equiv) were then added in succession. After 14 h of stirring at rt, the mixture was poured into a separatory funnel containing 20 mL CH_2Cl_2 and 10 mL DI-H_2O and extracted with 3 x 20 mL CH_2Cl_2. The organic layer was washed with 10 mL brine, dried over Na_2SO_4, filtered, and concentrated. Purification by silica column chromatography (1:15 EtOAc/hexanes) afforded **7c** as a clear oil (53 mg, 51%). ^{1}H NMR (500 MHz, $CDCl_3$) δ 7.43 (d, 2H, *J* = 8.6 Hz), 7.39 (d, 1H, *J* = 8.1 Hz), 7.15–7.17 (m, 2H), 7.05 (d, 2H, *J* = 8.6 Hz), 5.26 (s, 2H), 3.30 (s, 3H), 3.22 (s, 1H), 2.63 (s, 1H), 1.56–2.00 (m, 12H); ^{13}C NMR (125 MHz, $CDCl_3$) δ 152.53, 146.75, 141.60, 140.66, 135.76, 133.75, 131.22, 130.24, 129.86, 128.13, 125.37, 123.76, 119.23, 70.12, 58.04, 39.09, 38.07, 37.00, 32.15, 30.31, 28.14. LRMS (MALDI) calcd for $C_{26}H_{26}ClN_3O_4$ (M^+) 479.1612, found 479.2970.

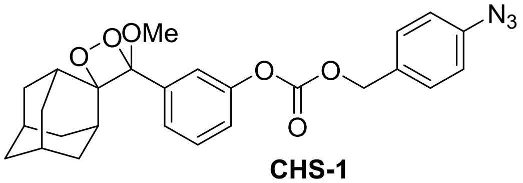

CHS-1

4-azidobenzyl (3-((1*r*, 3*r*, 5*r*, 7*r*) - 4' - methoxyspiro [adamantane - 2, 3' - [1,2] dioxetan] - 4' -yl) phenyl) carbonate (CHS-1). Compound **7a** (75 mg, 0.17 mmol, 1.0 equiv) and Rose bengal (8.5 mg, 0.0087 mmol, 0.051 equiv) were added into a dry flask and dissolved in 5 mL THF. Oxygen was bubbled through the reaction mixture, while irradiating with a 120 W light bulb (Home Depot, Dallas, TX) at 0 °C. After 3.5 h of reaction, TLC showed no starting material left and the mixture was then concentrated under vacuum at 0 ºC and the residue was purified by the silica column chromatography (1:15 EtOAc/hexanes) to deliver **CHS-1** as a white solid (56.4 mg, 70 %). ^{1}H NMR (500 MHz, $CDCl_3$) δ 7.42–7.92 (br m, 3H), 7.42–7.44 (m, 2H), 7.23 (dd, 1H, *J* = 9.2, 2.3 Hz), 7.05 (d, 2H, *J* = 8.6 Hz), 5.23 (s, 2H), 3.21 (s, 3H), 3.02 (s, 1H), 2.12 (s, 1H), 1.20–1.90 (m, 12H); ^{13}C NMR (125 MHz, $CDCl_3$) δ 153.43, 151.08, 140.82, 136.73, 131.34, 130.42, 129.33, 122.10, 119.39, 111.58, 95.52, 69.94, 50.13, 36.44, 34.74, 32.94, 32.82, 32.35, 31.77, 31.50 25.93, 25.81.

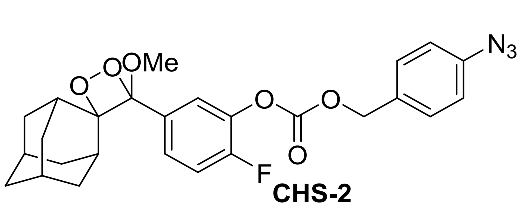

CHS-2

4-azidobenzyl (2-fluoro-5-((1*r*, 3*r*, 5*r*, 7*r*) - 4' -methoxyspiro [adamantane - 2, 3' - [1,2] dioxetan] - 4' -yl)phenyl) carbonate (CHS-2). Compound **7b** (47.6 mg, 0.103 mmol, 1 equiv) and Rose bengal (7 mg, 0.007 mmol, 0.07 equiv) were added into a dry flask and dissolved in 5.0 mL THF. Oxygen was bubbled through the reaction mixture, while irradiating with a 120 W light bulb (Home Depot, Dallas, TX) at 0–5 °C. After 6 h of reaction, the mixture was

concentrated under vacuum at 0 ºC. Purification by silica column chromatography (1:15 EtOAc/hexanes) provided **CHS-2** as a white solid (32.5 mg, 66%). ^{1}H NMR (500 MHz, $CDCl_3$) δ 7.40–7.91 (br m, 2H), 7.41–7.44 (m, 2H), 7.20–7.24 (m, 1H), 7.04–7.06 (d, 2H, J = 8.6 Hz), 5.25 (s, 2H), 3.21 (s, 3H), 3.01 (s, 1H), 2.07 (s, 1H), 0.90–2.00 (m, 12H); ^{13}C NMR (125 MHz, $CDCl_3$) δ 155.47, 153.46, 152.48, 140.79, 138.33, 131.61, 130.98, 130.26, 119.29, 116.82, 116.67, 111.06, 95.37, 70.34, 50.00, 39.25, 36.26, 34.78, 33.13, 32.87, 32.19, 31.67, 31.50, 25.93, 25.78.

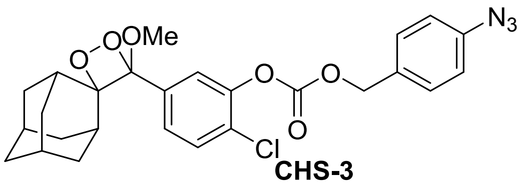

CHS-3

4-azidobenzyl (2-chloro-5-((1*r*, 3*r*, 5*r*, 7*r*) - 4' -methoxyspiro [adamantane - 2, 3' - [1,2] dioxetan] - 4' -yl)phenyl) carbonate (CHS-3). Compound **7c** (37.3 mg, 0.0777 mmol, 1 equiv) and Rose bengal (6 mg, 0.006 mmol, 0.08 equiv) were added into a dry flask and dissolved in 4.0 mL THF. Oxygen was bubbled through the reaction mixture, while irradiating with a 120 W light bulb (Home Depot, Dallas, TX) at 0–5 °C. The reaction was monitored by TLC. When TLC showed no starting material, the mixture was concentrated under vacuum at 0 ºC. The residue was purified by silica column chromatography (1:15 EtOAc/hexanes). Compound **CHS-3** was obtained as white solid (36.1 mg, 91%). ^{1}H NMR (500 MHz, $CDCl_3$) δ 7.10–7.89 (br m, 2H), 7.49–7.56 (m, 1H), 7.42–7.44 (m, 2H), 7.03–7.07 (m, 2H), 5.26 (s, 2H), 3.21 (s, 1H), 3.01 (s, 1H), 2.07 (s, 1H), 0.96–1.90 (m, 12H); ^{13}C NMR (125 MHz, $CDCl_3$) δ 152.41, 147.07, 140.76, 135.31, 131.07, 130.23, 130.10, 128.12, 119.26, 111.00, 95.38, 70.28, 50.04, 36.25, 34.78, 33.11, 32.93, 32.17, 31.69, 31.50, 25.93, 25.78; LRMS (ESI) calcd for $C_{26}H_{26}ClN_3O_6Na$ ($M+Na^+$) 534.1408, found 534.200.

2. Chemiluminescent response

Chemiluminescent responses and time scans were acquired using a Hitachi F-7000 Fluorescence Spectrophotometer (Hitachi, Tokyo, Japan) using the luminescence detection module and setting emission wavelength to 545 nm. 393 μL of a 20 mM HEPES buffered to pH 7.4 (Figure 1) or 100 mM glycine buffered to pH 10.02, 100 μL Emerald II Enhancer (Life Technologies, Carlsbad, CA), 5 μL of a 20 mM Na_2S stock solution in DI-H_2O and 2 μL of a 10 mM stock solution of **CHS** probes in CH_3CN were added to a quartz cuvette (Starna, Atascadero, CA). Samples were shaken gently to assure mixing. Then chemiluminescence spectra were acquired immediately after adding the probes. Time scans were acquired using the time scan module. 40 μM **CHS-1**, **CHS-2**, and **CHS-3** were treated with 0, 5, 10, 20, 40, 80, 100, 150, and 200 μM Na_2S,[1] and 10 min (Figure 1, Figure S1) or 120 min (Figure S2) time scans were measured 1 min after adding probes.

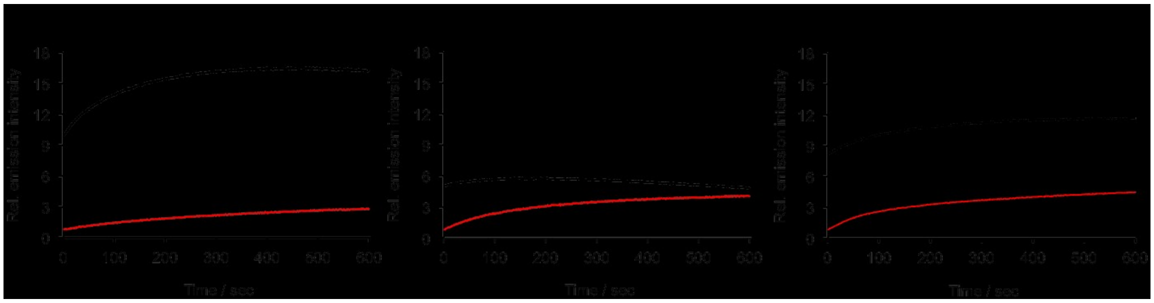

Figure S1. Time scans of the chemiluminescence emission at 545 nm from (a) 40 µM **CHS-1**, (b) 40 µM **CHS-2**, or (c) 40 µM **CHS-3** and 0 µM (red) or 200 µM (black) Na_2S in 100 mM glycine buffer (pH 10.02) containing 20% Emerald II Enhancer.

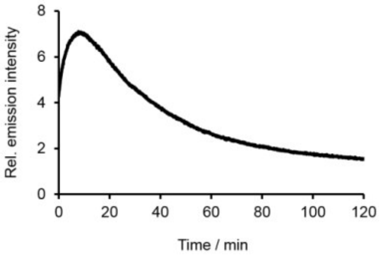

Figure S2. Full time scan of the chemiluminescence emission at 545 nm of 40 µM **CHS-3** to 200 µM Na_2S in 20 mM HEPES buffer (pH 7.4) and 20% Emerald II Enhancer.

3. Selectivity tests

Selectivity for **CHS-1**, **CHS-2**, and **CHS-3** was measured by monitoring the time-dependent chemiluminescent emission at 545 nm. All assays were performed in 20 mM HEPES buffered to pH 7.4 with 20% Emerald II Enhancer.

<u>H_2S:</u> 5 µL of a 20 mM stock solution of Na_2S in DI-H_2O was added to a solution of 393 µL HEPES and 100 µL Emerald II Enhancer and then 2 µL of 10 mM **CHS-1**, **CHS-2**, or **CHS-3** in CH_3CN was added into this mixture.

<u>H_2S, glutathione, cysteine and homocysteine:</u> 5 µL of a 20 mM stock solution of Na_2S in DI-H_2O, 25 µL of a 100 mM stock solution of glutathione in 20 mM HEPES buffer, 5 µL of a 100 mM stock solution of L-cysteine in DI-H_2O and 5 µL of a 100 mM stock solution of homocysteine in DI-H_2O were added to a solution of 358 µL HEPES and 100 µL Emerald II Enhancer, mixed them well and then 2 µL of 10 mM **CHS-1**, **CHS-2**, or **CHS-3** in CH_3CN was added into this mixture.

Glutathione: 25 μL of a 100 mM stock solution of glutathione in 20 mM HEPES buffer was added to a solution of 373 μL HEPES and 100 μL Emerald II Enhancer and then 2 μL of 10 mM **CHS-1**, **CHS-2**, or **CHS-3** in CH_3CN was added into this mixture.

S-nitrosoglutathione: 2 μL of a 50 mM stock solution of *S*-nitrosoglutathione in DI-H_2O was added to a solution of 396 μL HEPES and 100 μL Emerald II Enhancer and then 2 μL of 10 mM **CHS-1**, **CHS-2**, or **CHS-3** in CH_3CN was added into this mixture.

Cysteine: 5 μL of a 100 mM stock solution of L-cysteine in DI-H_2O was added to a solution of 393 μL HEPES and 100 μL Emerald II Enhancer and then 2 μL of 10 mM **CHS-1**, **CHS-2**, or **CHS-3** in CH_3CN was added into this mixture.

Homocysteine: 5 μL of a 100 mM stock solution of homocysteine in DI-H_2O was added to a solution of 393 μL HEPES and 100 μL Emerald II Enhancer and then 2 μL of 10 mM **CHS-1**, **CHS-2**, or **CHS-3** in CH_3CN was added into this mixture.

HNO: Angeli's salt ($Na_2N_2O_3$) was used to generate HNO. The stock solution was made by dissolving Angeli's salt in 0.01 M NaOH solution immediately prior to use. The concentration of this alkaline stock solution of Angeli's salt was measured by UV/Vis using $\varepsilon = 6100\ M^{-1}\ cm^{-1}$ at 237 nm. 4 μL of a 25 mM stock solution of Angeli's salt was added to a solution of 394 μL HEPES and 100 μL Emerald II Enhancer and then 2 μL of 10 mM **CHS-1**, **CHS-2**, or **CHS-3** in CH_3CN was added into this mixture.

NO: PROLI NONOate was used to generate NO. It was stored at –80 °C and dissolved in 0.01 M NaOH solution immediately prior to use. The concentration of this alkaline stock solution of PROLI NONOate was measured by UV/Vis using $\varepsilon = 8400\ M^{-1}\ cm^{-1}$ at 252 nm. 8 μL of a 12.5 mM stock solution of PROLI NONOate was added to a solution of 390 μL HEPES and 100 μL Emerald II Enhancer and then 2 μL of 10 mM **CHS-1**, **CHS-2**, or **CHS-3** in CH_3CN was added into this mixture.

$NaNO_2$: 1 μL of a 100 mM stock solution of $NaNO_2$ in DI-H_2O was added to a solution of 397 μL HEPES and 100 μL Emerald II Enhancer and then 2 μL of 10 mM **CHS-1**, **CHS-2**, or **CHS-3** in CH_3CN was added into this mixture.

Na_2SO_3: 1 μL of a 100 mM stock solution of Na_2SO_3 in DI-H_2O was added to a solution of 397 μL HEPES and 100 μL Emerald II Enhancer and then 2 μL of 10 mM **CHS-1**, **CHS-2**, or **CHS-3** in CH_3CN was added into this mixture.

H_2O_2: 0.5 μL of a 200 mM stock solution of H_2O_2 in DI-H_2O was added to a solution of 397.5 μL HEPES and 100 μL Emerald II Enhancer and then 2 μL of 10 mM **CHS-1**, **CHS-2**, or **CHS-3** in CH_3CN was added into this mixture.

OCl⁻: 1 μL of a 100 mM stock solution of OCl^- in DI-H_2O was added to a solution of 397 μL HEPES and 100 μL Emerald II Enhancer and then 2 μL of 10 mM **CHS-1**, **CHS-2**, or **CHS-3** in CH_3CN was added into this mixture.

^{t}BuOOH: 1 μL of a 100 mM stock solution of ^{t}BuOOH in DI-H_2O was added to a solution of 397 μL HEPES and 100 μL Emerald II Enhancer and then 2 μL of 10 mM **CHS-1**, **CHS-2**, or **CHS-3** in CH_3CN was added into this mixture.

Blank: 2 μL of 10 mM **CHS-1**, **CHS-2**, or **CHS-3** in CH_3CN was added to a solution of 398 μL HEPES and 100 μL Emerald II Enhancer.

4. Computational results

All the geometries were optimized using density functional theory (DFT) with B3LYP[2,3,4,5] functional and Pople basis set 6-311+G(d,p)[6] and integral equation formalism of polarizable continuum model (IEF-PCM)[7] with water as solvent. Atomic charges are calculated using ESP model.[8] The ESP charges were also calculated at using M06[9] and ωB97XD[10] functionals and 6-311+G(d,p) basis set using IEF-PCM with water as solvent at geometries optimized at B3LYP/6-311+G(d,p) level of theory. All the calculations were carried out using Gaussian09.[11]

Table S1. Cartesian coordinates and ESP charges of the phenolate released from CHS-1.

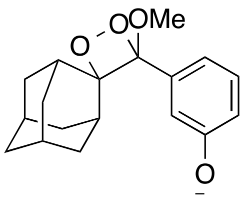

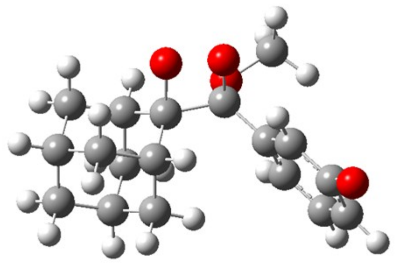

Atoms	Coordinates			ESP Charges		
	X	Y	Z	B3LYP	M06	ωB97XD
C	-0.525984	1.150508	-0.203135	0.545303	0.471105	0.495804
C	-1.727570	0.285391	0.124617	0.099203	0.109287	0.117666
C	-2.083655	0.092001	1.470495	-0.271377	-0.284134	-0.297094
C	-3.175031	-0.730180	1.760514	-0.119742	-0.103717	-0.119251
C	-3.910823	-1.346960	0.754537	-0.489714	-0.496664	-0.505737
C	-3.590809	-1.171395	-0.630272	0.732688	0.718372	0.726573
C	-2.458164	-0.327224	-0.890878	-0.482501	-0.479113	-0.497363
H	-2.177752	-0.170785	-1.926113	0.134117	0.133390	0.146024
H	-1.524221	0.571638	2.263229	0.127048	0.128149	0.141148
H	-3.457109	-0.889776	2.798020	0.132324	0.132286	0.144371
H	-4.757334	-1.980048	1.006384	0.158441	0.159698	0.170194
O	0.886032	1.289159	-1.758716	-0.234040	-0.220551	-0.218705
O	-0.552137	1.592492	-1.592039	-0.274019	-0.253500	-0.257995
O	-0.388964	2.245425	0.662459	-0.359828	-0.340657	-0.344376

C	-1.458996	3.199452	0.641049	-0.051678	-0.065903	-0.097505
H	-1.581264	3.620117	-0.360353	0.065677	0.073081	0.082881
H	-2.400056	2.752689	0.970625	0.046074	0.053406	0.062674
H	-1.168394	3.989972	1.331850	0.096263	0.103440	0.112077
C	0.894931	0.542654	-0.476542	-0.301032	-0.315180	-0.331668
C	2.072956	0.986778	0.395329	0.459439	0.424814	0.466294
C	0.982428	-0.967402	-0.749027	0.477663	0.443191	0.485612
C	3.387488	0.686966	-0.361529	-0.516053	-0.509129	-0.576764
H	1.987177	2.056259	0.588102	-0.008413	0.006917	0.008296
C	2.058656	0.202573	1.726486	-0.545194	-0.536331	-0.607069
C	0.980257	-1.748010	0.585087	-0.622453	-0.594505	-0.674162
C	2.300998	-1.258371	-1.500814	-0.561967	-0.543775	-0.618448
H	0.129315	-1.269331	-1.360870	0.021265	0.029948	0.033563
H	4.232097	1.032165	0.244944	0.105916	0.112922	0.129743
H	3.419685	1.247622	-1.301616	0.105207	0.113368	0.128594
C	3.502403	-0.823911	-0.638910	0.667617	0.611593	0.670457
H	2.895262	0.542800	2.347007	0.123243	0.128594	0.147093
H	1.140768	0.414965	2.283265	0.088636	0.098837	0.113524
C	2.177369	-1.308027	1.448003	0.642633	0.596155	0.651186
H	0.044439	-1.583005	1.124784	0.124333	0.119936	0.144045
H	1.041301	-2.819977	0.366152	0.130318	0.133016	0.152250
H	2.313705	-0.730443	-2.459250	0.111743	0.117263	0.133609
H	2.355114	-2.329825	-1.722358	0.115670	0.119557	0.138788
C	3.491446	-1.595465	0.695969	-0.615873	-0.609456	-0.682179
H	3.588945	-2.671348	0.509269	0.115291	0.122012	0.139607
H	4.349881	-1.296970	1.309330	0.107426	0.115414	0.132729
H	4.433667	-1.028357	-1.177878	-0.075734	-0.056419	-0.058091
O8	-4.262270	-1.729367	-1.578754	-0.945304	**-0.924794**	-0.945863
H	2.166090	-1.856290	2.395996	-0.058611	-0.041926	-0.042532

Table S2. Cartesian coordinates and ESP charges of the phenolate released from CHS-2.

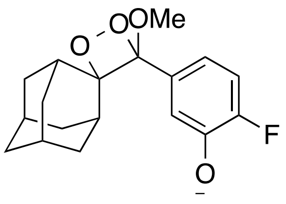

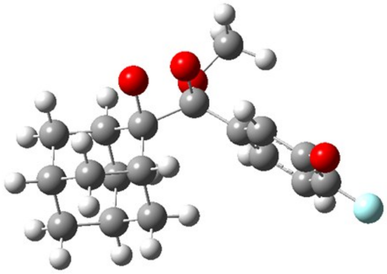

Atoms	Coordinates			ESP Charges		
	X	Y	Z	B3LYP	M06	ωB97XD
C	0.149490	1.295067	0.147303	0.666428	0.613317	0.635134
C	1.447231	0.538942	-0.058484	0.027595	0.039860	0.041685
C	1.888437	0.280587	-1.364665	-0.227740	-0.236361	-0.248862
C	3.069166	-0.445270	-1.543795	-0.313850	-0.299083	-0.314726
C	3.778432	-0.886334	-0.446454	0.164508	0.153261	0.151692
C	3.403682	-0.658579	0.912787	0.533141	0.527766	0.531079

C	2.183736	0.082379	1.035332	-0.460427	-0.466600	-0.478759
H	1.837314	0.289506	2.040882	0.156613	0.157543	0.168896
H	1.330477	0.635373	-2.220796	0.146178	0.145784	0.158982
H	3.442171	-0.668581	-2.538004	0.189167	0.189068	0.201163
O	-1.329697	1.410147	1.641708	-0.221708	-0.205948	-0.205321
O	0.071893	1.857398	1.489683	-0.299493	-0.282719	-0.286349
O	-0.061869	2.285941	-0.821477	-0.377468	-0.362749	-0.365580
C	0.891813	3.356861	-0.842148	-0.081532	-0.095256	-0.128613
H	0.906569	3.878428	0.118145	0.073610	0.080273	0.090500
H	1.895042	2.995397	-1.080186	0.048046	0.053884	0.064272
H	0.555873	4.040912	-1.620331	0.107245	0.114679	0.123568
C	-1.210061	0.563606	0.429631	-0.410019	-0.439958	-0.450678
C	-2.391332	0.806026	-0.514636	0.550796	0.513778	0.556319
C	-1.151011	-0.920189	0.826641	0.543890	0.511907	0.555224
C	-3.697592	0.428257	0.220997	-0.589371	-0.579830	-0.649153
H	-2.410032	1.859872	-0.793122	-0.015794	0.000026	0.001376
C	-2.239512	-0.075751	-1.773927	-0.601316	-0.585706	-0.660233
C	-1.011108	-1.799538	-0.436741	-0.698894	-0.666739	-0.749373
C	-2.461963	-1.290788	1.555754	-0.639683	-0.616966	-0.693655
H	-0.298253	-1.080127	1.490472	0.020949	0.028655	0.031905
H	-4.547150	0.629361	-0.440989	0.121087	0.127371	0.144632
H	-3.828272	1.057647	1.107516	0.122295	0.130426	0.145595
C	-3.665526	-1.058674	0.621959	0.723048	0.664696	0.725968
H	-3.080441	0.121741	-2.448015	0.130847	0.135013	0.154067
H	-1.326501	0.187841	-2.316539	0.093118	0.101384	0.117278
C	-2.211125	-1.562837	-1.372594	0.720043	0.668780	0.726384
H	-0.076344	-1.578680	-0.958022	0.130602	0.125136	0.149630
H	-0.968073	-2.850972	-0.131205	0.148104	0.149685	0.169644
H	-2.569150	-0.693651	2.466582	0.132276	0.137128	0.153565
H	-2.412389	-2.340833	1.863828	0.133851	0.136174	0.155828
C	-3.517799	-1.929125	-0.642510	-0.729769	-0.717799	-0.794224
H	-3.508855	-2.990497	-0.368443	0.144229	0.149606	0.167941
H	-4.376625	-1.775332	-1.306291	0.133322	0.140041	0.158105
H	-4.591809	-1.318302	1.145321	-0.069040	-0.050168	-0.051929
O8	4.088841	-1.078040	1.913968	-0.880513	**-0.862369**	-0.882378
H	-2.102631	-2.181758	-2.269492	-0.057694	-0.041053	-0.041458
F	4.938444	-1.596528	-0.673229	-0.286675	-0.285937	-0.279140

Table S3. Cartesian coordinates and ESP charges of the phenolate released from CHS-3.

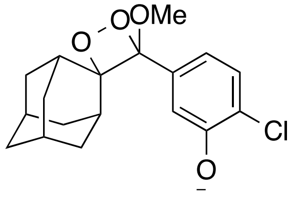

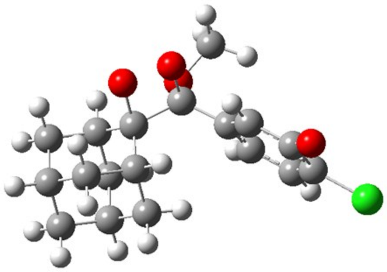

Atoms	Coordinates			ESP Charges		
	X	Y	Z	B3LYP	M06	ωB97XD
C	-0.226616	1.379308	0.084256	0.477044	0.391441	0.434096
C	1.136262	0.725086	-0.038113	0.275155	0.290053	0.286684
C	1.629921	0.398308	-1.312558	-0.304850	-0.316975	-0.331510
C	2.869203	-0.231300	-1.410975	-0.140200	-0.104783	-0.128008
C	3.603953	-0.523739	-0.270801	-0.197996	-0.250474	-0.222810
C	3.169669	-0.211460	1.060595	0.789065	0.792540	0.789128
C	1.883594	0.429928	1.096313	-0.669246	-0.662266	-0.678151
H	1.500428	0.687659	2.076325	0.185804	0.183499	0.196519
H	1.064628	0.630276	-2.205377	0.146778	0.146802	0.160845
H	3.267367	-0.495785	-2.384260	0.151077	0.144161	0.159847
O	-1.739421	1.449315	1.548388	-0.250273	-0.236758	-0.234096
O	-0.386703	2.022435	1.381224	-0.269303	-0.248082	-0.254511
O	-0.491411	2.279085	-0.956871	-0.375806	-0.353336	-0.360094
C	0.373729	3.420331	-1.035139	-0.037390	-0.053041	-0.084873
H	0.315745	4.011511	-0.117817	0.068619	0.076932	0.086152
H	1.409742	3.125859	-1.219015	0.031909	0.038941	0.048745
H	0.009101	4.014271	-1.872134	0.098118	0.105477	0.114263
C	-1.524543	0.550098	0.387797	-0.220732	-0.229779	-0.256661
C	-2.704067	0.643036	-0.584689	0.460276	0.424371	0.468665
C	-1.352091	-0.899530	0.867956	0.502259	0.465498	0.514064
C	-3.987842	0.203875	0.156948	-0.558241	-0.548514	-0.619949
H	-2.801982	1.674432	-0.923733	0.001334	0.015586	0.017705
C	-2.462727	-0.293736	-1.789133	-0.562438	-0.547368	-0.624448
C	-1.122839	-1.834929	-0.341825	-0.666226	-0.632362	-0.717206
C	-2.641055	-1.333302	1.602224	-0.639705	-0.614850	-0.696101
H	-0.500127	-0.952239	1.549769	0.017717	0.025071	0.028669
H	-4.840580	0.299402	-0.524073	0.119066	0.125338	0.142934
H	-4.180994	0.868846	1.005179	0.116431	0.123826	0.139779
C	-3.844179	-1.250708	0.642918	0.680415	0.619023	0.684559
H	-3.305652	-0.202710	-2.483282	0.131749	0.135932	0.155508
H	-1.565258	0.011735	-2.335771	0.087984	0.096743	0.112776
C	-2.322938	-1.748524	-1.302431	0.638117	0.583720	0.647335
H	-0.200433	-1.569401	-0.864446	0.125111	0.119818	0.144461
H	-1.000393	-2.860142	0.024952	0.146882	0.148543	0.168391

H	-2.810960	-0.696788	2.475937	0.132890	0.136716	0.154634
H	-2.513222	-2.357182	1.969865	0.138955	0.141353	0.161574
C	-3.607974	-2.176718	-0.567081	-0.680569	-0.667777	-0.747001
H	-3.519229	-3.217008	-0.232864	0.139088	0.144225	0.163023
H	-4.465128	-2.130460	-1.249182	0.127329	0.134405	0.152458
H	-4.755108	-1.553702	1.169924	-0.060987	-0.041332	-0.043684
O	3.839950	-0.470633	2.117156	-0.907189	**-0.894123**	-0.909589
H	-2.151668	-2.406450	-2.160957	-0.041069	-0.023678	-0.025356
Cl	5.179273	-1.329405	-0.464458	-0.206955	-0.184515	-0.198766

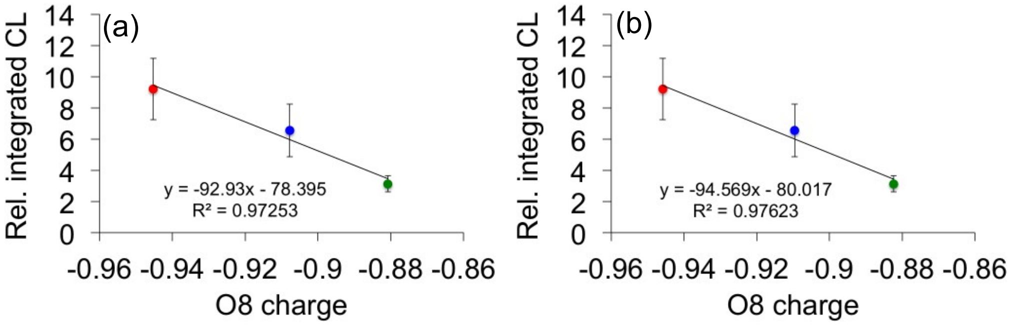

Figure S3. Plot of the integrated chemiluminescent emission over 10 min at pH 10 of 200 μM Na_2S and 40 μM **CHS-1** (red), **CHS-2** (green) and **CHS-3** (blue) versus the calculated atomic charges on the phenolate oxygen (O8). All luminescent measurements were acquired in 100 mM glycine (pH 10) containing 20% Emerald II Enhancer. The reported values are averages of the integrated emission intensities over 10 min (n = 4–7). Error bars represent ± S.D. Geometries were optimized with B3LYP/6-311G+(d,p) and ESP atomic charges were calculated with (a) B3LYP/6-311+G(d,p) or (b) ωB97XD/6-311+G(d,p). Calculations were carried out with the IEF-PCM water solvation model using Gaussian 09.

5. Cellular experiments

Chemiluminescent response using a multi-well plate reader. Chemiluminescent responses were measured using a BioTek plate reader (Winooski, VT) by using the luminescence detection method, endpoint read type, and setting sensitivity to 135. 120 μL, 119 μL, 119 μL, 118 μL, and 116 μL of 20 mM HEPES buffer (pH 7.4) were added into the wells of a black opaque Corning® 96-well plate from A1 to A5 in sequence, 30 μL Emerald II Enhancer was pipetted into each well, then different volumes of a 10 mM Na_2S solution (0 μL, 0.38 μL, 0.75 μL, 1.5 μL, 3.0 μL) were added into each well. 0.60 μL **CHS-3** was injected into each well and the luminescence intensity of the plate was measured every 2 min after addition of probes. The detection limit was estimated as the amount of Na_2S required to give a chemiluminescent signal above three times the standard deviation of at least 3 independent experiments with 0 μM Na_2S. The concentration of Na_2S needed was estimated by fitting a line to the linear region of the curve between the data points corresponding to 0 μM Na_2S and 25 μM Na_2S.

Cell culture and detecting cellular H_2S using a multi-well plate reader. Human lung adenocarcinoma epithelial cell (A549) were purchased from ATCC and cultured in F-12K media supplemented with 10% Fetal Calf Serum (FCS) at 37 °C with 5% CO_2. Two days before the

experiment, cells were passed and plated on Costar® 12-well plates by adding 150K–175K of A549 cells per well, filling each well up to 600 µL of media with FCS, and aspirating the media upon 90–95% confluence. Cells were serum-starved for 18 h prior to the experiment. Stock solutions of 20 mM homocysteine and 20 mM D,L-propargyl glycine (PAG) were prepared in 20 mM HEPES buffer (pH 7.4) and 10 mM **CHS-3** was prepared in CH_3CN. 6 µL PAG (final concentration: 200 µM) was added to C1–C3 of the 12-well plate. After 20 min incubation, 6 µL homocysteine (final concentration: 200 µM) was added into B1–B3 and C1–C3 of the 12-well plate, and 6 µL 20 mM HEPES buffer (pH 7.4) as a vehicle control was added to A1–A3. After another 20 min incubation, cells were washed with 1 x PBS. Then 500 µL PBS media was added into each well after aspirating the media. 125 µL Emerald II Enhancer and 2.7 µL **CHS-3** (final concentration: 40 µM) were added to each well and the luminescent intensity was measured every two minutes for 20 minutes. The experiment was repeated with four independent well plates and the peak value for the luminescence emission for each well at 10–12 minutes was normalized to the average luminescence emission at 10–12 minutes for the control replicates of each plate. A single outlier was rejected according to the extreme studentized deviate method (Grubbs' test, $p < 0.01$).

6. Imaging experiments

Chemiluminescent imaging at pH 7.4. Imaging was carried out with a Caliper Xenogen IVIS® Spectrum (Perkin-Elmer, Santa Clara, CA) in black 96-well Costar® plates and all the images were analyzed using Living Image 3.1 software. 10 mM **CHS-3** in CH_3CN and 10 mM Na_2S in DI-H_2O were prepared prior to imaging. 199 µL, 198 µL, 198 µL, 197 µL and 195 µL of 20 mM HEPES buffer (pH 7.4) were added into wells from A1 to A5 in sequence, 50 µL Emerald II Enhancer was pipetted into each well, then different volume of Na_2S solution (0 µL, 0.61 µL, 1.25 µL, 2.5 µL, 5.0 µL) were added into each well. 1 µL **CHS-3** was injected into the mixture and imaging was performed after 30 seconds using an open filter. All images were acquired with f-stop 1, medium binning, auto exposure and the chamber set to 37 °C.

Imaging H_2S in mouse carcass. A stock solution of 25 mM **CHS-3** in DMSO and 50 mM Na_2S in DI-H_2O were prepared in advance. The 50 mM stock solution of Na_2S was diluted to provide a final concentration of 4 mM Na_2S in 100 µL (0.4 µmol) to be injected. Images were acquired 30 sec after administering i.p. injections to the carcasses of SCID/BALB-C mice with 0.08 µmol **CHS-3** and either 0.4 µmol Na_2S or a vehicle control (H_2O) in HEPES buffered at pH 7.4 containing 20% Emerald II Enhancer (Figure S4a–d).

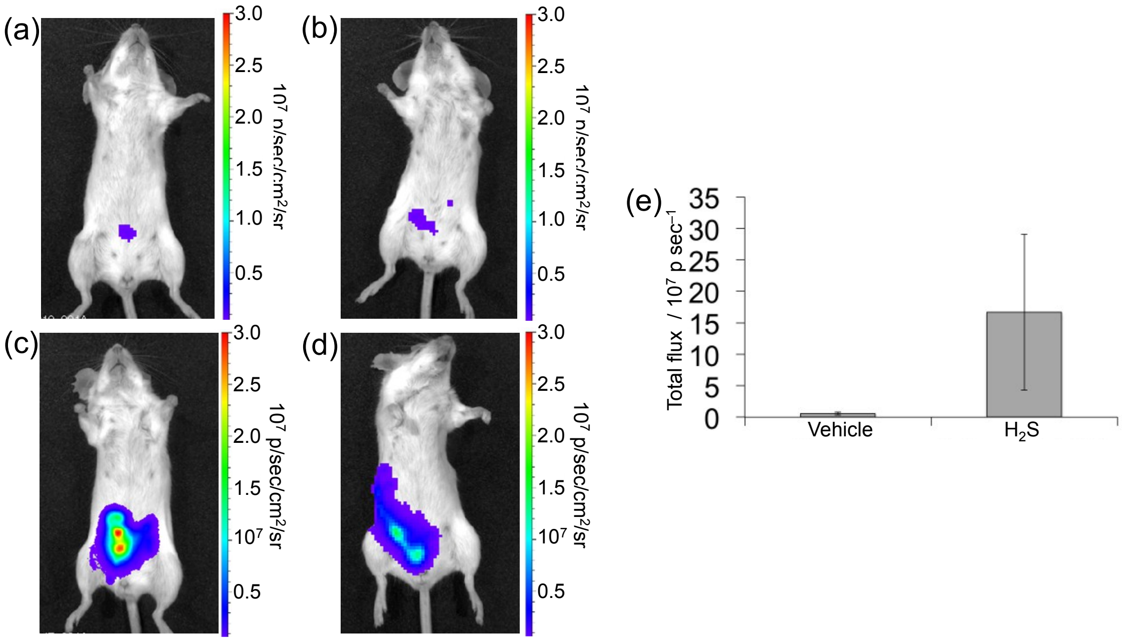

Figure S4. Imaging H_2S in SCID/BALB-C mouse carcasses using **CHS-3**. Images were obtained 30 sec after administering an i.p. injection of 0.08 μmol **CHS-3** and (a–b) vehicle control or (c–d) 0.4 μmol Na_2S in 100 μL 20 mM HEPES at pH 7.4 containing 20% Emerald II Enhancer. (e) Quantification of the total photon flux from experiments described in (a)–(d). Error bars are ± S.D.

Imaging H_2S in living mice. The UT Southwestern Institutional Animal Care and Use Committee approved these investigations under APN #2009-0150. A stock solution of 25 mM **CHS-3** in DMSO and 50 mM Na_2S in DI-H_2O were prepared in advance. The 50 mM stock solution of Na_2S was diluted to provide a final concentration of 4 mM Na_2S in 100 μL (0.4 μmol) to be injected. Images were acquired 30 sec after administering i.p. injections to C6 brown mice with 0.08 μmol **CHS-3** and either 0.4 μmol Na_2S or a vehicle control (H_2O) in HEPES buffered at pH 7.4 containing 20% Emerald II Enhancer (Figure S5a–f). The skin was raised during injections to avoid puncturing internal organs. The final concentration of Na_2S in the injection was 4 mM.

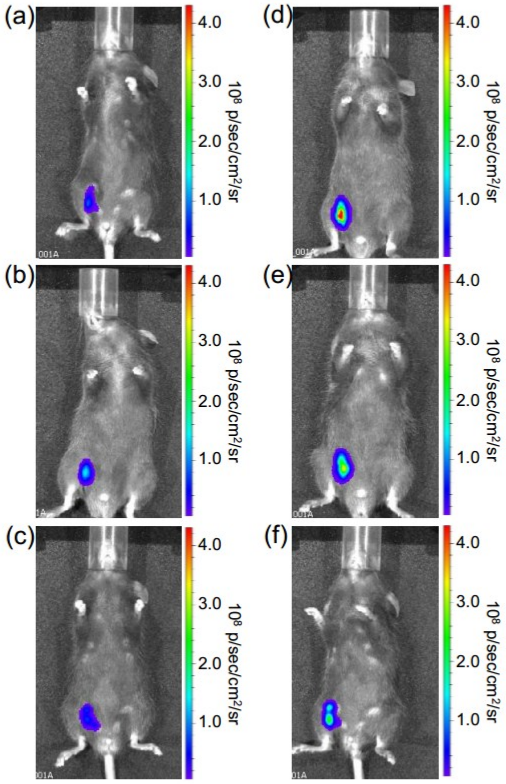

Figure S5. Imaging H_2S in living C6 brown mice using **CHS-3**. Images were taken 30 sec after administering an i.p. injection of 0.08 µmol **CHS-3** and (a–c) vehicle control or (d–f) 0.4 µmol Na_2S in 100 µL 20 mM HEPES at pH 7.4 containing 20% Emerald II Enhancer.

7. ^{1}H and ^{13}C NMR spectra

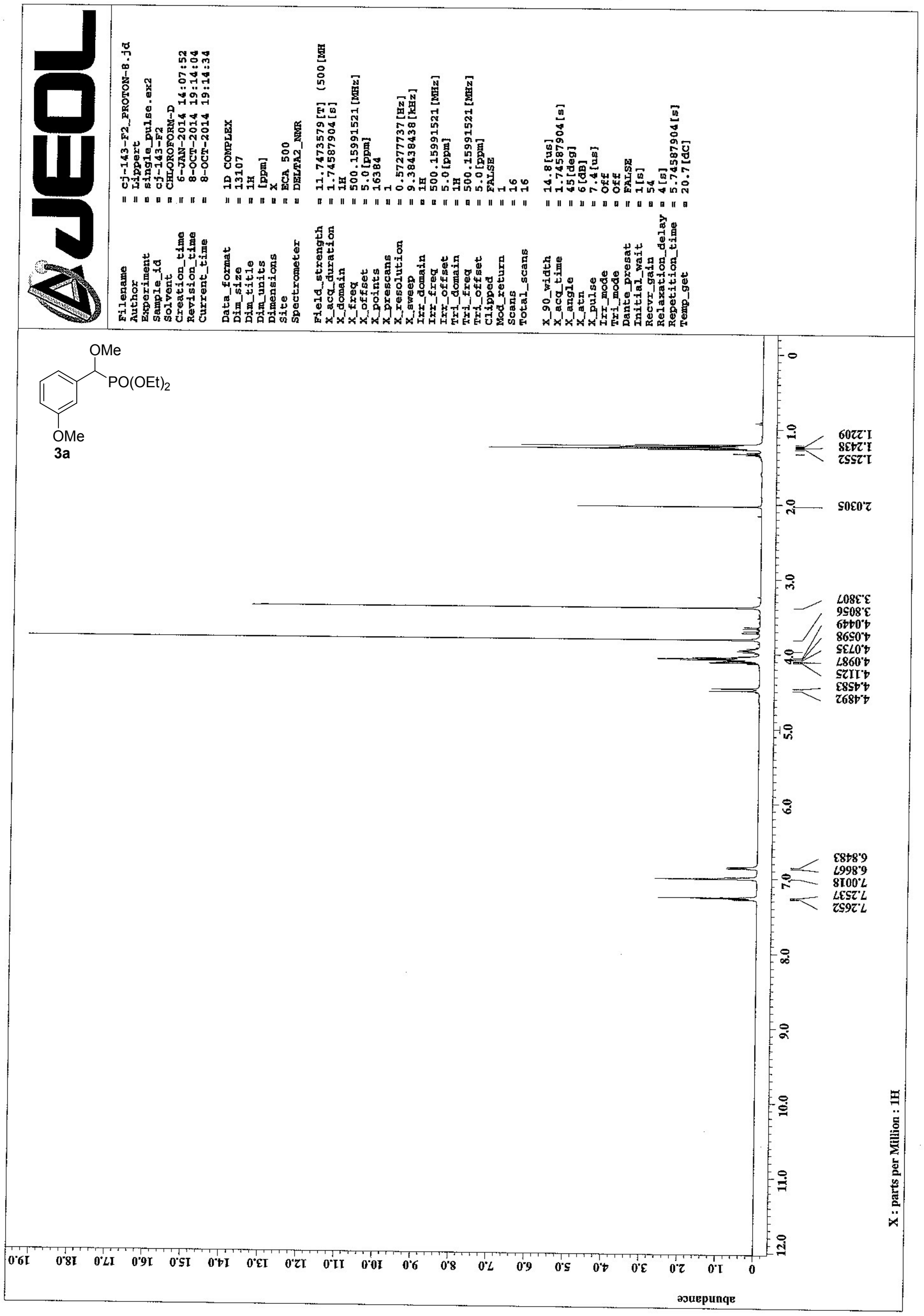

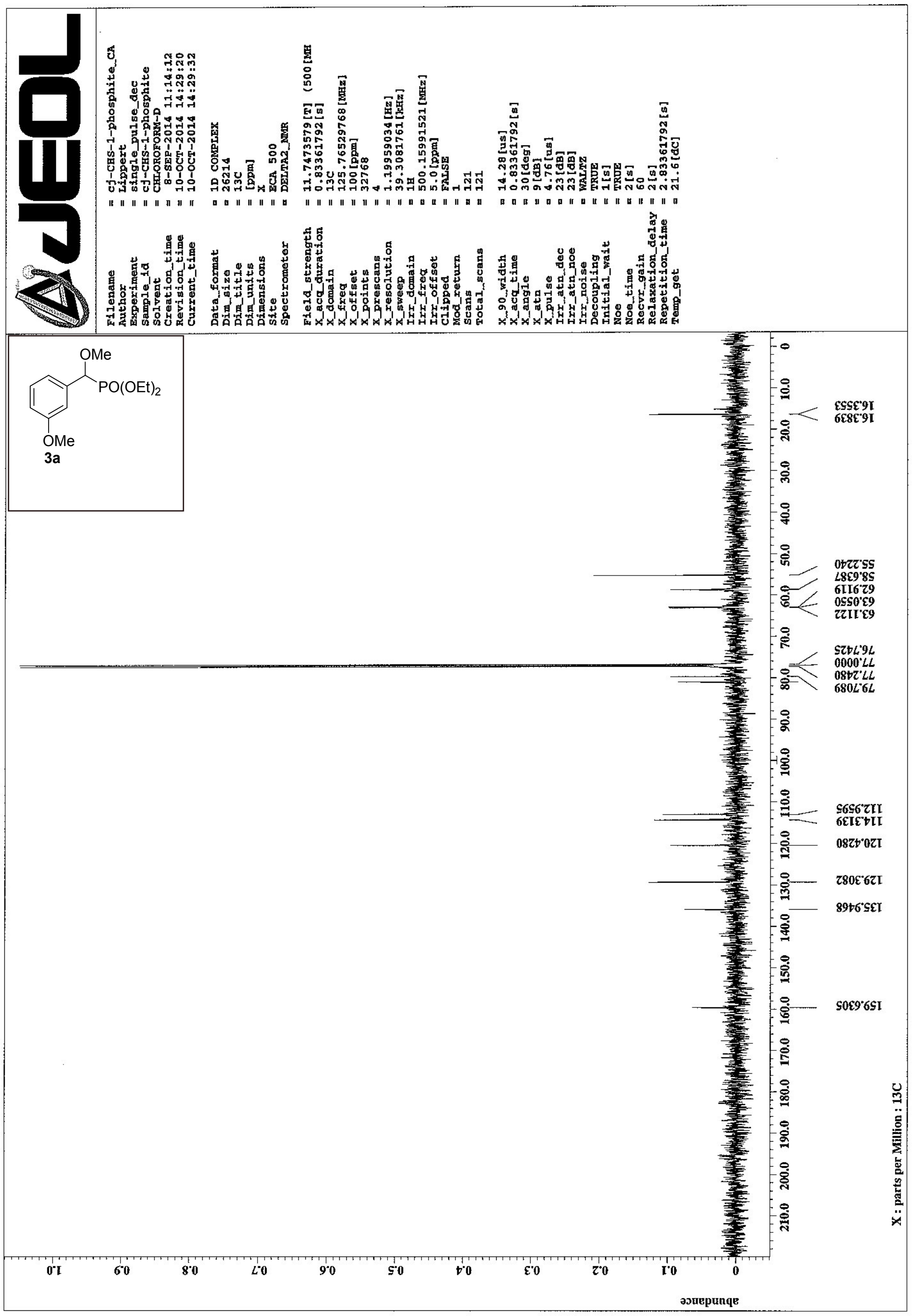
OMe
PO(OEt)2
OMe
3a
JEOL
Filename = cj-CHS-1-phosphite_CA
Author = Lippert
Experiment = single_pulse_dec
Sample_id = cj-CHS-1-phosphite
Solvent = CHLOROFORM-D
Creation_time = 8-SEP-2014 11:14:12
Revision_time = 10-OCT-2014 14:29:20
Current_time = 10-OCT-2014 14:29:32
Data_format = 1D COMPLEX
Dim_size = 26214
Dim_title = 13C
Dim_units = [ppm]
Dimensions = X
Site = ECA 500
Spectrometer = DELTA2_NMR
Field_strength = 11.7473579[T] (500[MH
X_acq_duration = 0.83361792[s]
X_domain = 13C
X_freq = 125.76529768[MHz]
X_offset = 100[ppm]
X_points = 32768
X_prescans = 4
X_resolution = 1.19959034[Hz]
X_sweep = 39.3081761[kHz]
Irr_domain = 1H
Irr_freq = 500.15991521[MHz]
Irr_offset = 5.0[ppm]
Clipped = FALSE
Mod_return = 1
Scans = 121
Total_scans = 121
X_90_width = 14.28[us]
X_acq_time = 0.83361792[s]
X_angle = 30[deg]
X_atn = 9[dB]
X_pulse = 4.76[us]
Irr_atn_dec = 23[dB]
Irr_atn_noe = 23[dB]
Irr_noise = WALTZ
Decoupling = TRUE
Initial_wait = 1[s]
Noe = TRUE
Noe_time = 2[s]
Recvr_gain = 60
Relaxation_delay = 2[s]
Repetition_time = 2.83361792[s]
Temp_get = 21.6[dC]
16.3553
16.3839
55.2240
58.6387
62.9119
63.0550
63.1122
76.7425
77.0000
77.2480
79.7089
112.9595
114.3139
120.4280
129.3082
135.9468
159.6305
X : parts per Million : 13C
abundance

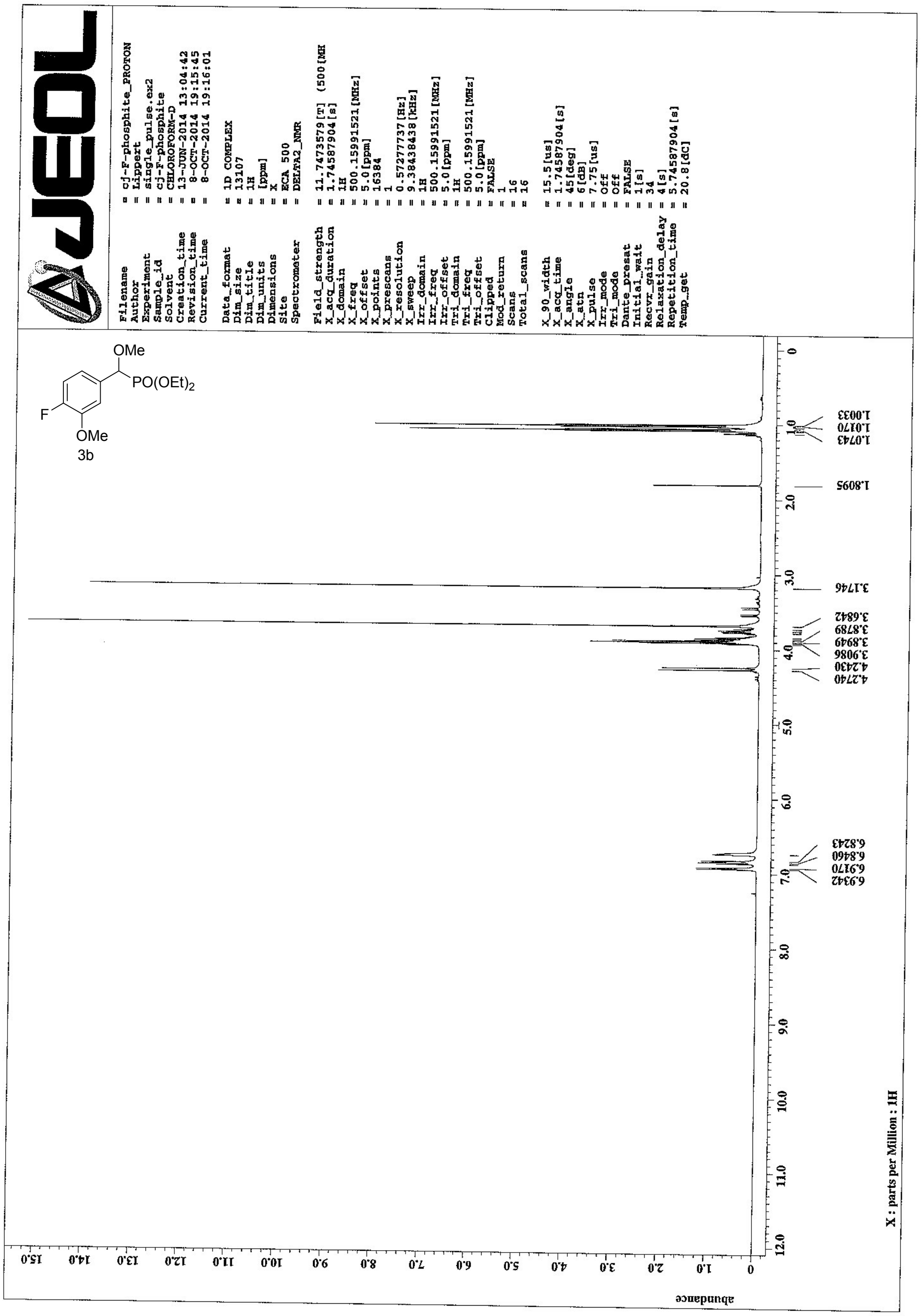

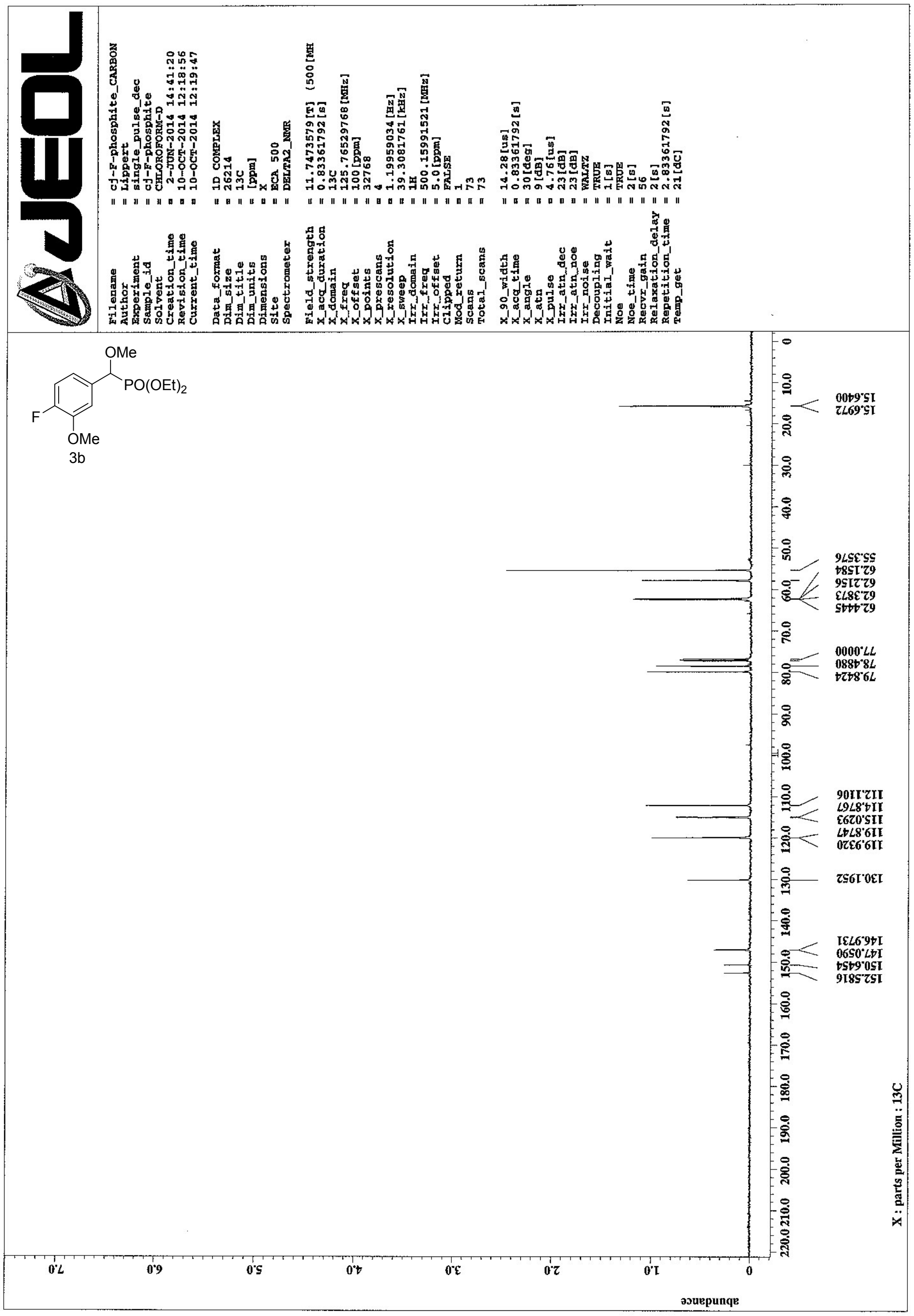
OMe
PO(OEt)2
F
OMe
3b
Filename = cj-F-phosphite_CARBON
Author = Lippert
Experiment = single_pulse_dec
Sample_id = cj-F-phosphite
Solvent = CHLOROFORM-D
Creation_time = 2-JUN-2014 14:41:20
Revision_time = 10-OCT-2014 12:18:56
Current_time = 10-OCT-2014 12:19:47
Data_format = 1D COMPLEX
Dim_size = 26214
Dim_title = 13C
Dim_units = [ppm]
Dimensions = X
Site = ECA 500
Spectrometer = DELTA2_NMR
Field_strength = 11.7473579[T] (500[MH
X_acq_duration = 0.83361792[s]
X_domain = 13C
X_freq = 125.76529768[MHz]
X_offset = 100[ppm]
X_points = 32768
X_prescans = 4
X_resolution = 1.19959034[Hz]
X_sweep = 39.3081761[kHz]
Irr_domain = 1H
Irr_freq = 500.15991521[MHz]
Irr_offset = 5.0[ppm]
Clipped = FALSE
Mod_return = 1
Scans = 73
Total_scans = 73
X_90_width = 14.28[us]
X_acq_time = 0.83361792[s]
X_angle = 30[deg]
X_atn = 9[dB]
X_pulse = 4.76[us]
Irr_atn_dec = 23[dB]
Irr_atn_noe = 23[dB]
Irr_noise = WALTZ
Decoupling = TRUE
Initial_wait = 1[s]
Noe = TRUE
Noe_time = 2[s]
Recvr_gain = 56
Relaxation_delay = 2[s]
Repetition_time = 2.83361792[s]
Temp_get = 21[dC]
152.5816
150.6454
147.0590
146.9731
130.1952
119.9320
119.8747
115.0293
114.8767
112.1106
79.8424
78.4880
77.0000
62.4445
62.3873
62.2156
62.1584
55.3576
15.6972
15.6400
X : parts per Million : 13C
abundance

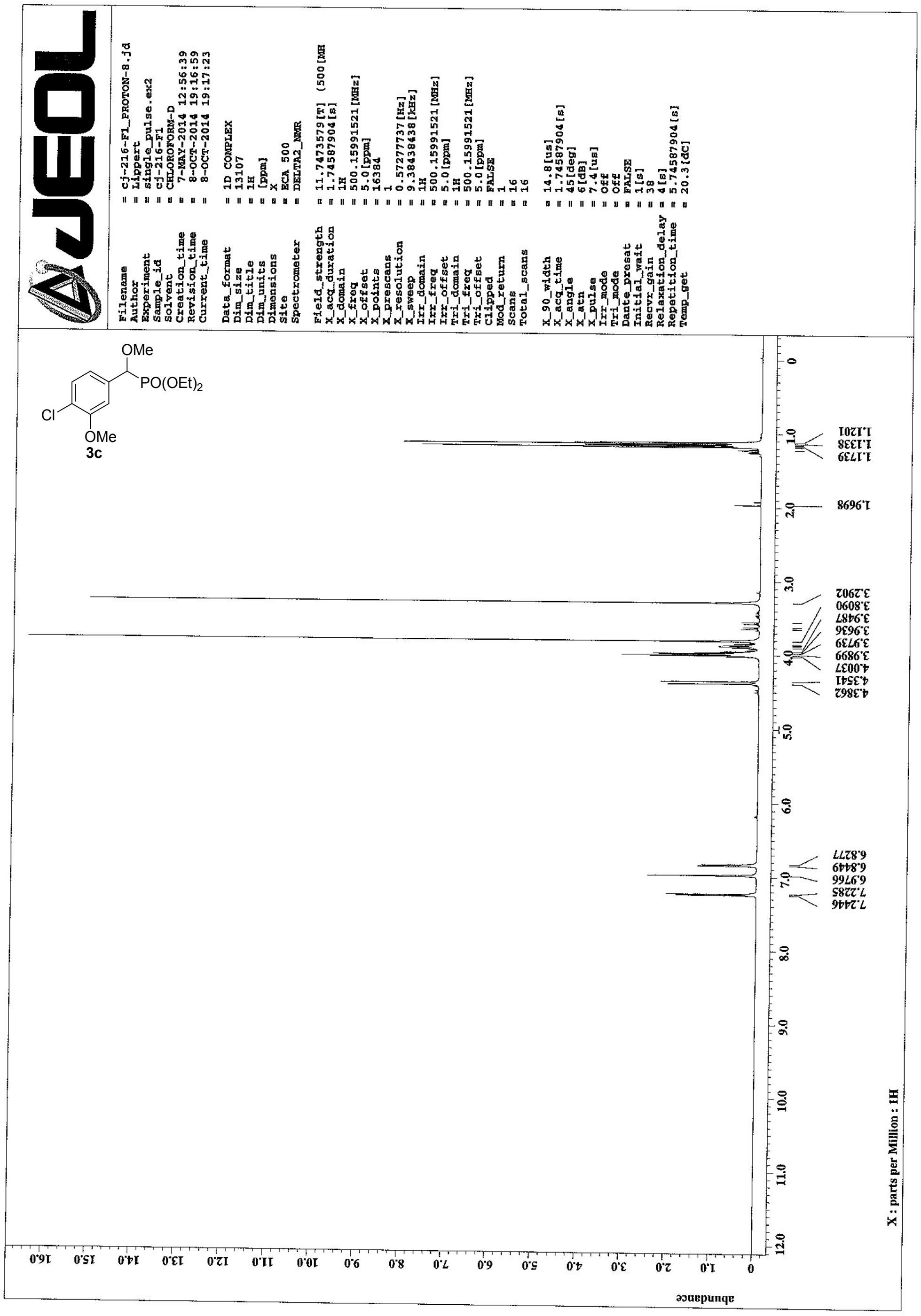

JEOL
Filename = cj-216-F1_PROTON-8.jd
Author = Lippert
Experiment = single_pulse.ex2
Sample_id = cj-216-F1
Solvent = CHLOROFORM-D
Creation_time = 7-MAY-2014 12:56:39
Revision_time = 8-OCT-2014 19:16:59
Current_time = 8-OCT-2014 19:17:23
Data_format = 1D COMPLEX
Dim_size = 13107
Dim_title = 1H
Dim_units = [ppm]
Dimensions = X
Site = ECA 500
Spectrometer = DELTA2_NMR
Field_strength = 11.7473579[T] (500[MH
X_acq_duration = 1.74587904[s]
X_domain = 1H
X_freq = 500.15991521[MHz]
X_offset = 5.0[ppm]
X_points = 16384
X_prescans = 1
X_resolution = 0.57277737[Hz]
X_sweep = 9.38438438[kHz]
Irr_domain = 1H
Irr_freq = 500.15991521[MHz]
Irr_offset = 5.0[ppm]
Tri_domain = 1H
Tri_freq = 500.15991521[MHz]
Tri_offset = 5.0[ppm]
Clipped = FALSE
Mod_return = 1
Scans = 16
Total_scans = 16
X_90_width = 14.8[us]
X_acq_time = 1.74587904[s]
X_angle = 45[deg]
X_atn = 6[dB]
X_pulse = 7.4[us]
Irr_mode = Off
Tri_mode = Off
Dante_presat = FALSE
Initial_wait = 1[s]
Recvr_gain = 38
Relaxation_delay = 4[s]
Repetition_time = 5.74587904[s]
Temp_get = 20.3[dC]
OMe
PO(OEt)2
Cl
OMe
3c
1.1201
1.1338
1.1739
1.9698
3.2902
3.8090
3.9487
3.9636
3.9739
3.9899
4.0037
4.3541
4.3862
6.8277
6.8449
6.9766
7.2285
7.2446
X : parts per Million : 1H
abundance

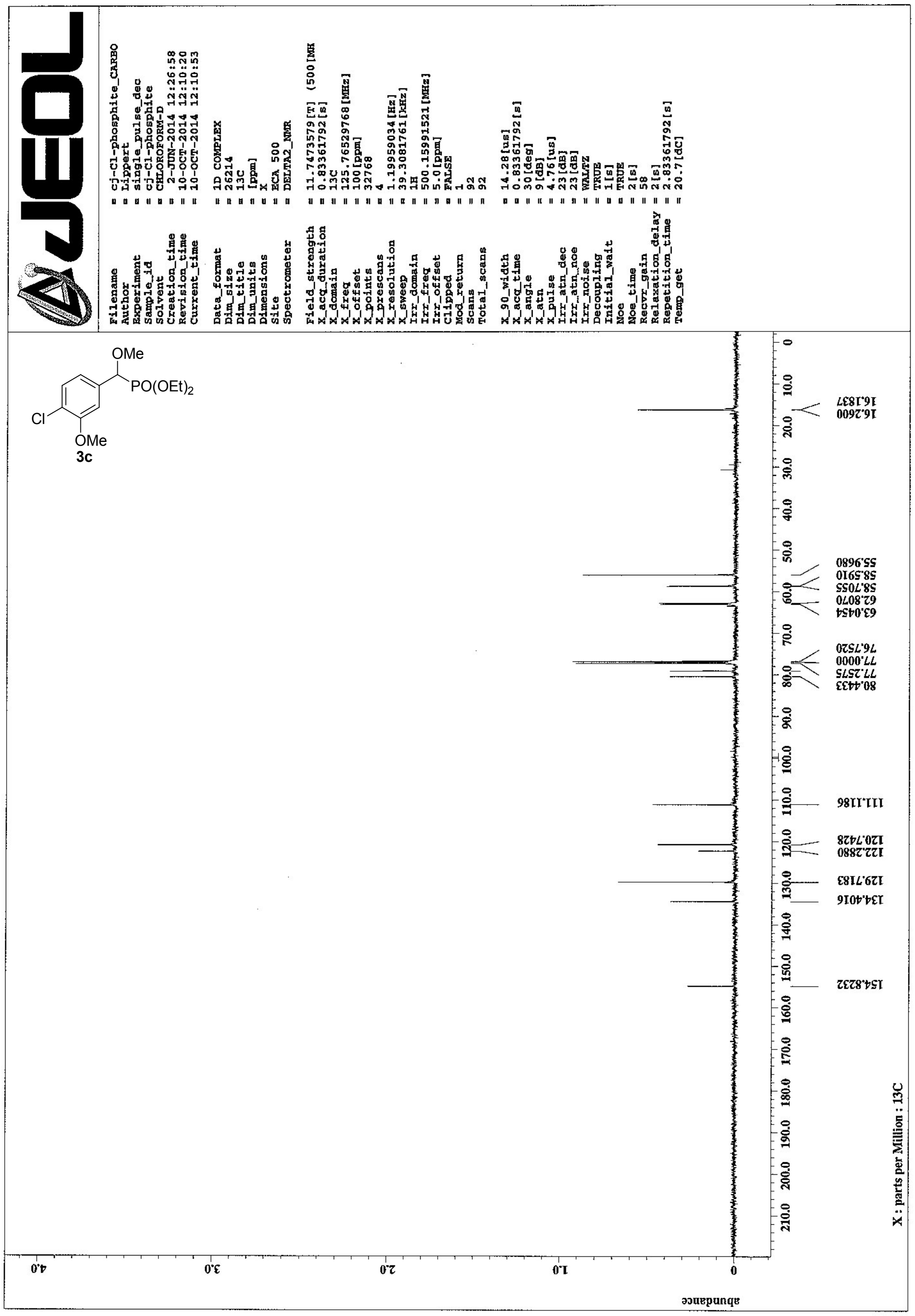
OMe
PO(OEt)2
Cl
OMe
3c
Filename = cj-Cl-phosphite_CARBO
Author = Lippert
Experiment = single_pulse_dec
Sample_id = cj-Cl-phosphite
Solvent = CHLOROFORM-D
Creation_time = 2-JUN-2014 12:26:58
Revision_time = 10-OCT-2014 12:10:20
Current_time = 10-OCT-2014 12:10:53
Data_format = 1D COMPLEX
Dim_size = 26214
Dim_title = 13C
Dim_units = [ppm]
Dimensions = X
Site = ECA 500
Spectrometer = DELTA2_NMR
Field_strength = 11.7473579[T] (500[MH
X_acq_duration = 0.83361792[s]
X_domain = 13C
X_freq = 125.76529768[MHz]
X_offset = 100[ppm]
X_points = 32768
X_prescans = 4
X_resolution = 1.19959034[Hz]
X_sweep = 39.3081761[kHz]
Irr_domain = 1H
Irr_freq = 500.15991521[MHz]
Irr_offset = 5.0[ppm]
Clipped = FALSE
Mod_return = 1
Scans = 92
Total_scans = 92
X_90_width = 14.28[us]
X_acq_time = 0.83361792[s]
X_angle = 30[deg]
X_atn = 9[dB]
X_pulse = 4.76[us]
Irr_atn_dec = 23[dB]
Irr_atn_noe = 23[dB]
Irr_noise = WALTZ
Decoupling = TRUE
Initial_wait = 1[s]
Noe = TRUE
Noe_time = 2[s]
Recvr_gain = 58
Relaxation_delay = 2[s]
Repetition_time = 2.83361792[s]
Temp_get = 20.7[dC]
16.1837
16.2600
55.9680
58.5910
58.7055
62.8070
63.0454
76.7520
77.0000
77.2575
80.4433
111.1186
120.7428
122.2880
129.7183
134.4016
154.8232
X : parts per Million : 13C
abundance

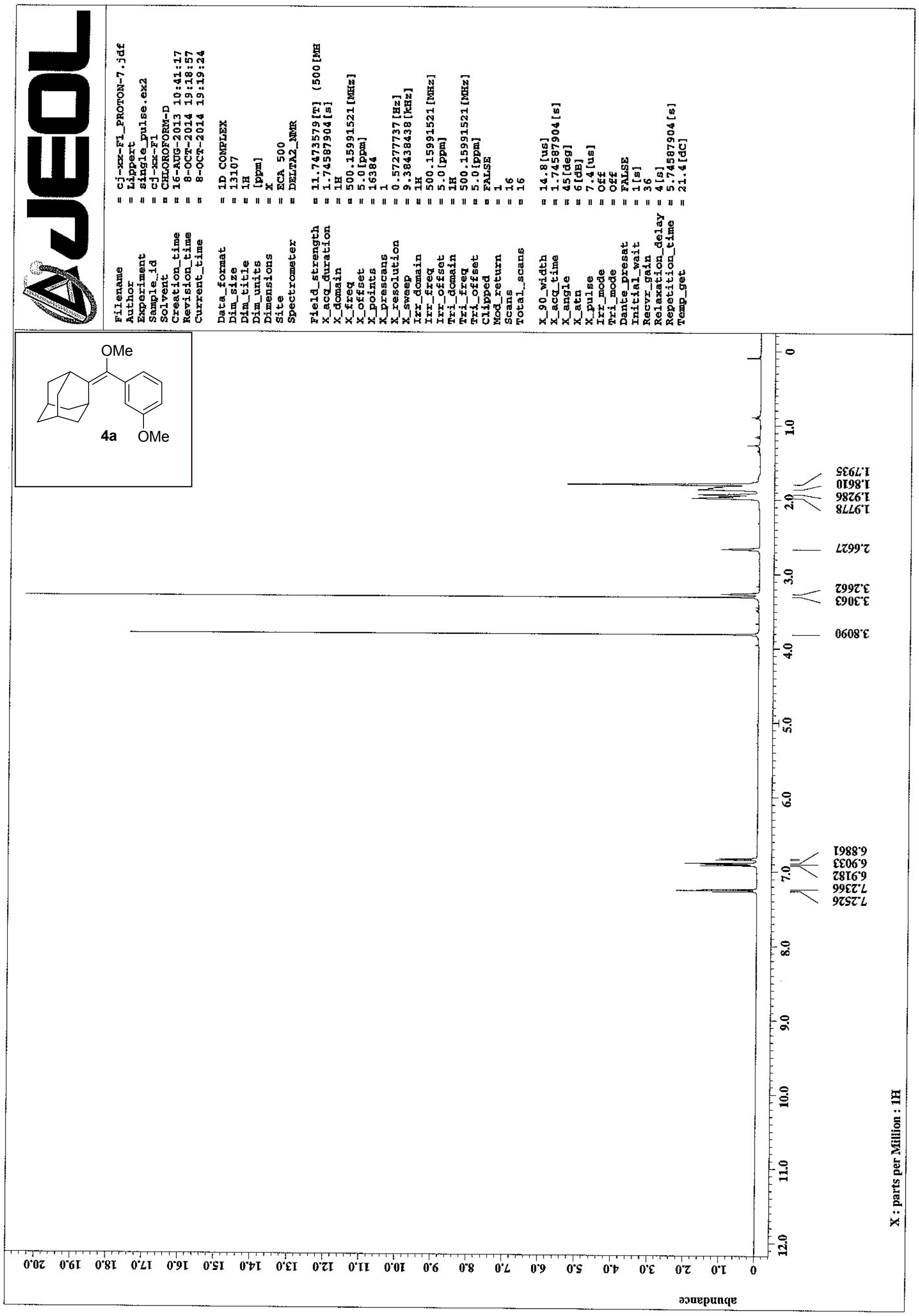
OMe
4a
OMe
JEOL
Filename = cj-xx-F1_PROTON-7.jdf
Author = Lippert
Experiment = single_pulse.ex2
Sample_id = cj-xx-F1
Solvent = CHLOROFORM-D
Creation_time = 16-AUG-2013 10:41:17
Revision_time = 8-OCT-2014 19:18:57
Current_time = 8-OCT-2014 19:19:24
Data_format = 1D COMPLEX
Dim_size = 13107
Dim_title = 1H
Dim_units = [ppm]
Dimensions = X
Site = ECA 500
Spectrometer = DELTA2_NMR
Field_strength = 11.7473579[T] (500[MH
X_acq_duration = 1.74587904[s]
X_domain = 1H
X_freq = 500.15991521[MHz]
X_offset = 5.0[ppm]
X_points = 16384
X_prescans = 1
X_resolution = 0.57277737[Hz]
X_sweep = 9.38438438[kHz]
Irr_domain = 1H
Irr_freq = 500.15991521[MHz]
Irr_offset = 5.0[ppm]
Tri_domain = 1H
Tri_freq = 500.15991521[MHz]
Tri_offset = 5.0[ppm]
Clipped = FALSE
Mod_return = 1
Scans = 16
Total_scans = 16
X_90_width = 14.8[us]
X_acq_time = 1.74587904[s]
X_angle = 45[deg]
X_atn = 6[dB]
X_pulse = 7.4[us]
Irr_mode = Off
Tri_mode = Off
Dante_presat = FALSE
Initial_wait = 1[s]
Recvr_gain = 36
Relaxation_delay = 4[s]
Repetition_time = 5.74587904[s]
Temp_get = 21.4[dC]
1.7935
1.8610
1.9286
1.9778
2.6627
3.2662
3.3063
3.8090
6.8861
6.9033
6.9182
7.2366
7.2526
X : parts per Million : 1H
abundance

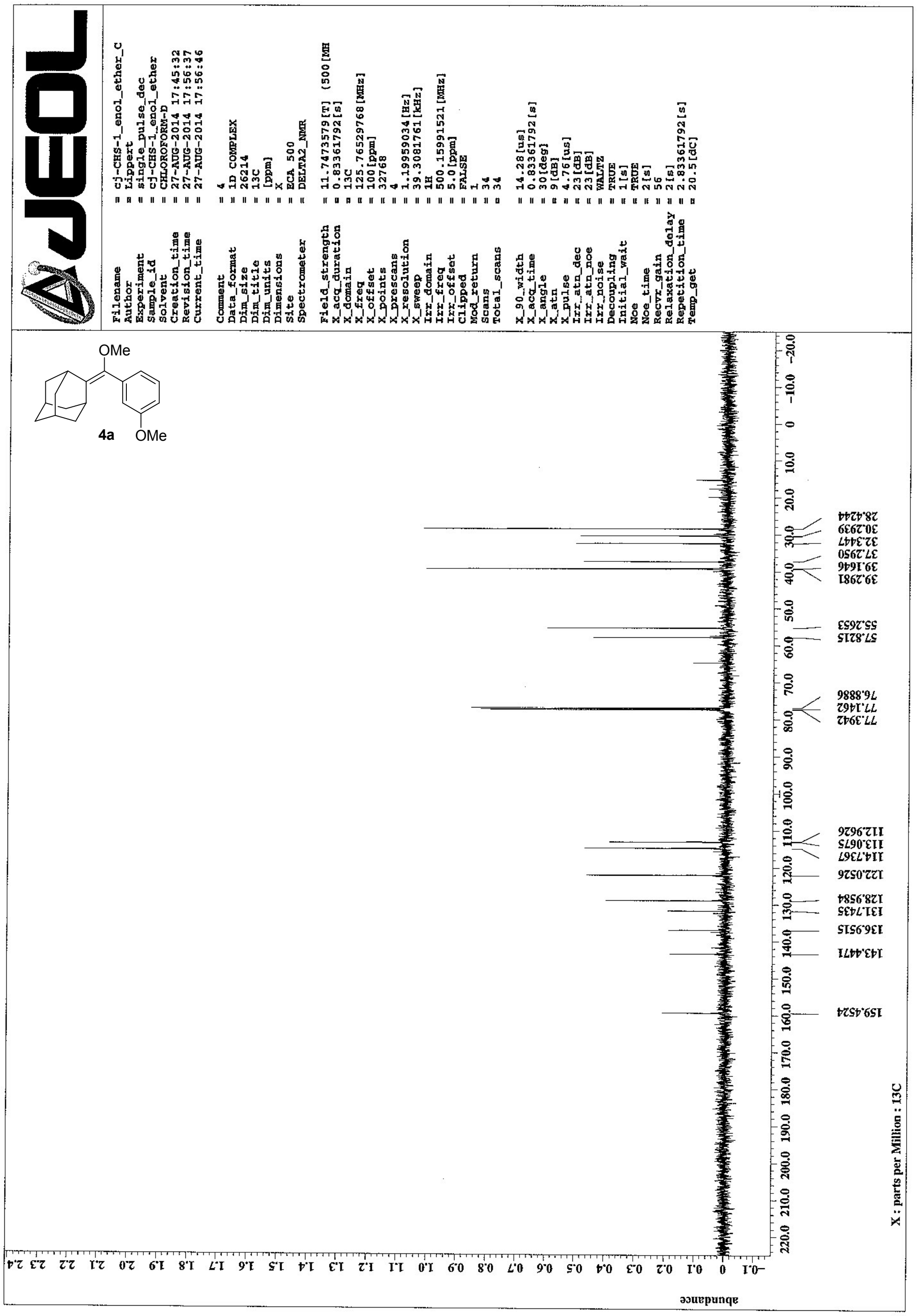
4a
OMe
OMe
Filename = cj-CHS-1_enol_ether_C
Author = Lippert
Experiment = single_pulse_dec
Sample_id = cj-CHS-1_enol_ether
Solvent = CHLOROFORM-D
Creation_time = 27-AUG-2014 17:45:32
Revision_time = 27-AUG-2014 17:56:37
Current_time = 27-AUG-2014 17:56:46
Comment = 4
Data_format = 1D COMPLEX
Dim_size = 26214
Dim_title = 13C
Dim_units = [ppm]
Dimensions = X
Site = ECA 500
Spectrometer = DELTA2_NMR
Field_strength = 11.7473579[T] (500[MH
X_acq_duration = 0.83361792[s]
X_domain = 13C
X_freq = 125.76529768[MHz]
X_offset = 100[ppm]
X_points = 32768
X_prescans = 4
X_resolution = 1.19959034[Hz]
X_sweep = 39.3081761[kHz]
Irr_domain = 1H
Irr_freq = 500.15991521[MHz]
Irr_offset = 5.0[ppm]
Clipped = FALSE
Mod_return = 1
Scans = 34
Total_scans = 34
X_90_width = 14.28[us]
X_acq_time = 0.83361792[s]
X_angle = 30[deg]
X_atn = 9[dB]
X_pulse = 4.76[us]
Irr_atn_dec = 23[dB]
Irr_atn_noe = 23[dB]
Irr_noise = WALTZ
Decoupling = TRUE
Initial_wait = 1[s]
Noe = TRUE
Noe_time = 2[s]
Recvr_gain = 56
Relaxation_delay = 2[s]
Repetition_time = 2.83361792[s]
Temp_get = 20.5[dC]
159.4524
143.4471
136.9515
131.7435
128.9584
122.0526
114.7367
113.0675
112.9626
77.3942
77.1462
76.8886
57.8215
55.2653
39.2981
39.1646
37.2950
32.3447
30.2939
28.4244
abundance
X : parts per Million : 13C

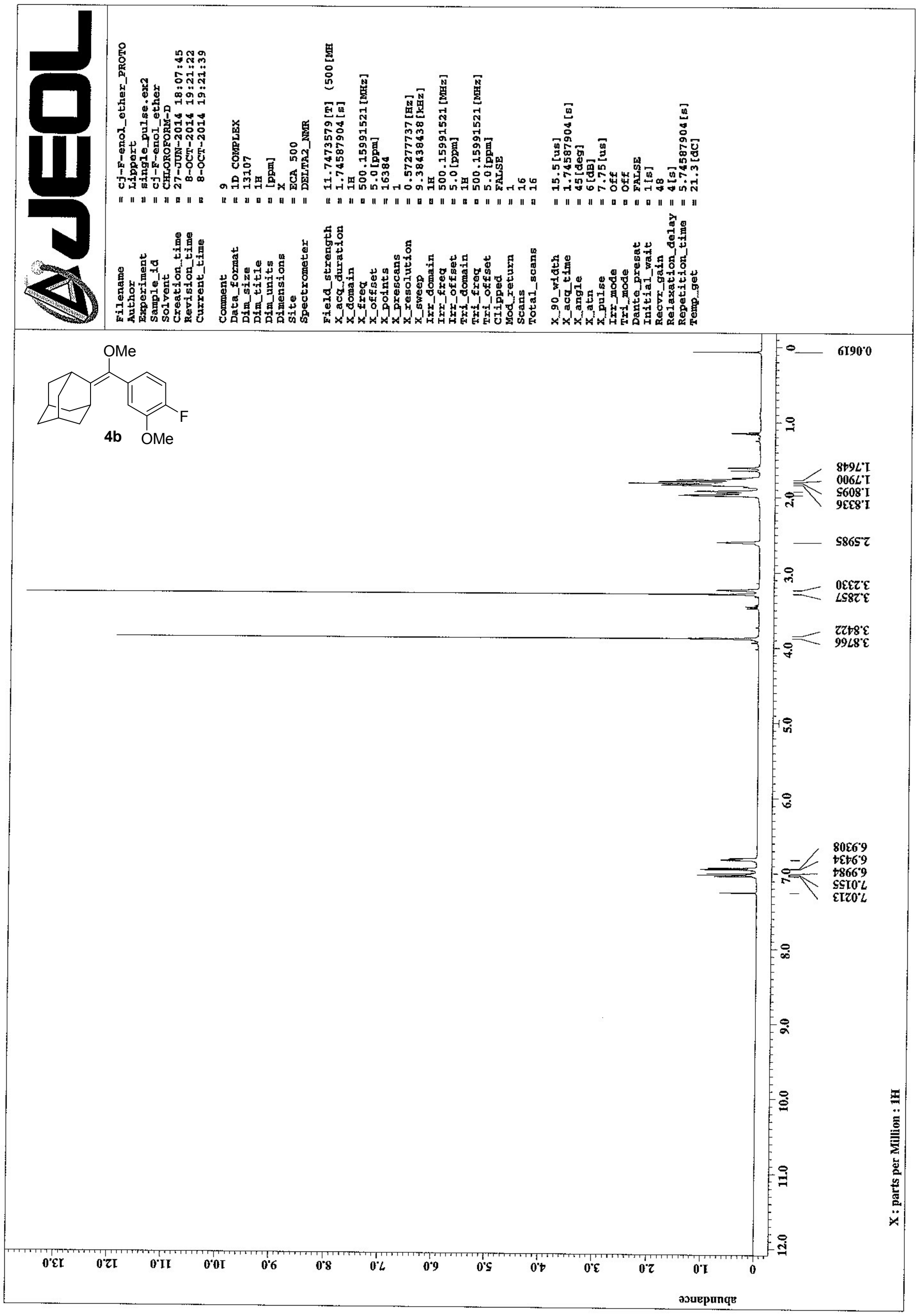
OMe
F
OMe
4b
JEOL
Filename = cj-F-enol_ether_PROTO
Author = Lippert
Experiment = single_pulse.ex2
Sample_id = cj-F-enol_ether
Solvent = CHLOROFORM-D
Creation_time = 27-JUN-2014 18:07:45
Revision_time = 8-OCT-2014 19:21:22
Current_time = 8-OCT-2014 19:21:39
Comment = 9
Data_format = 1D COMPLEX
Dim_size = 13107
Dim_title = 1H
Dim_units = [ppm]
Dimensions = X
Site = ECA 500
Spectrometer = DELTA2_NMR
Field_strength = 11.7473579[T] (500[MH
X_acq_duration = 1.74587904[s]
X_domain = 1H
X_freq = 500.15991521[MHz]
X_offset = 5.0[ppm]
X_points = 16384
X_prescans = 1
X_resolution = 0.57277737[Hz]
X_sweep = 9.38438438[kHz]
Irr_domain = 1H
Irr_freq = 500.15991521[MHz]
Irr_offset = 5.0[ppm]
Tri_domain = 1H
Tri_freq = 500.15991521[MHz]
Tri_offset = 5.0[ppm]
Clipped = FALSE
Mod_return = 1
Scans = 16
Total_scans = 16
X_90_width = 15.5[us]
X_acq_time = 1.74587904[s]
X_angle = 45[deg]
X_atn = 6[dB]
X_pulse = 7.75[us]
Irr_mode = Off
Tri_mode = Off
Dante_presat = FALSE
Initial_wait = 1[s]
Recvr_gain = 48
Relaxation_delay = 4[s]
Repetition_time = 5.74587904[s]
Temp_get = 21.3[dC]
abundance
7.0213
7.0155
6.9984
6.9434
6.9308
3.8766
3.8422
3.2857
3.2330
2.5985
1.8336
1.8095
1.7900
1.7648
0.0619
X : parts per Million : 1H

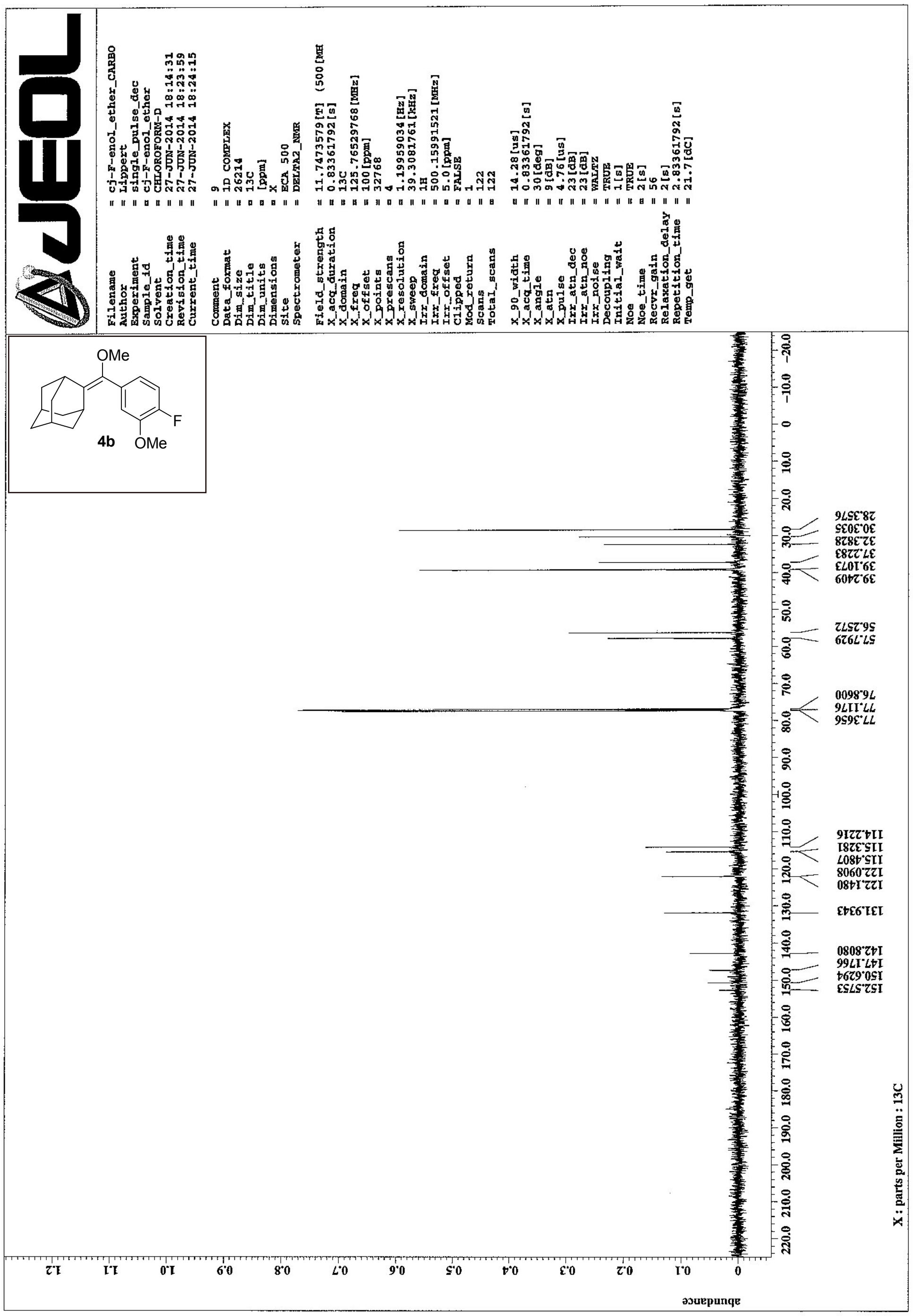
4b
OMe
F
OMe
JEOL
Filename = cj-F-enol_ether_CARBO
Author = Lippert
Experiment = single_pulse_dec
Sample_id = cj-F-enol_ether
Solvent = CHLOROFORM-D
Creation_time = 27-JUN-2014 18:14:31
Revision_time = 27-JUN-2014 18:23:59
Current_time = 27-JUN-2014 18:24:15
Comment = 9
Data_format = 1D COMPLEX
Dim_size = 26214
Dim_title = 13C
Dim_units = [ppm]
Dimensions = X
Site = ECA 500
Spectrometer = DELTA2_NMR
Field_strength = 11.7473579[T] (500[MH
X_acq_duration = 0.83361792[s]
X_domain = 13C
X_freq = 125.76529768[MHz]
X_offset = 100[ppm]
X_points = 32768
X_prescans = 4
X_resolution = 1.19959034[Hz]
X_sweep = 39.3081761[kHz]
Irr_domain = 1H
Irr_freq = 500.15991521[MHz]
Irr_offset = 5.0[ppm]
Clipped = FALSE
Mod_return = 1
Scans = 122
Total_scans = 122
X_90_width = 14.28[us]
X_acq_time = 0.83361792[s]
X_angle = 30[deg]
X_atn = 9[dB]
X_pulse = 4.76[us]
Irr_atn_dec = 23[dB]
Irr_atn_noe = 23[dB]
Irr_noise = WALTZ
Decoupling = TRUE
Initial_wait = 1[s]
Noe = TRUE
Noe_time = 2[s]
Recvr_gain = 56
Relaxation_delay = 2[s]
Repetition_time = 2.83361792[s]
Temp_get = 21.7[dC]
152.5753
150.6294
147.1766
142.8080
131.9343
122.1480
122.0908
115.4807
115.3281
114.2216
77.3656
77.1176
76.8600
57.7929
56.2572
39.2409
39.1073
37.2283
32.3828
30.3035
28.3576
X : parts per Million : 13C
abundance

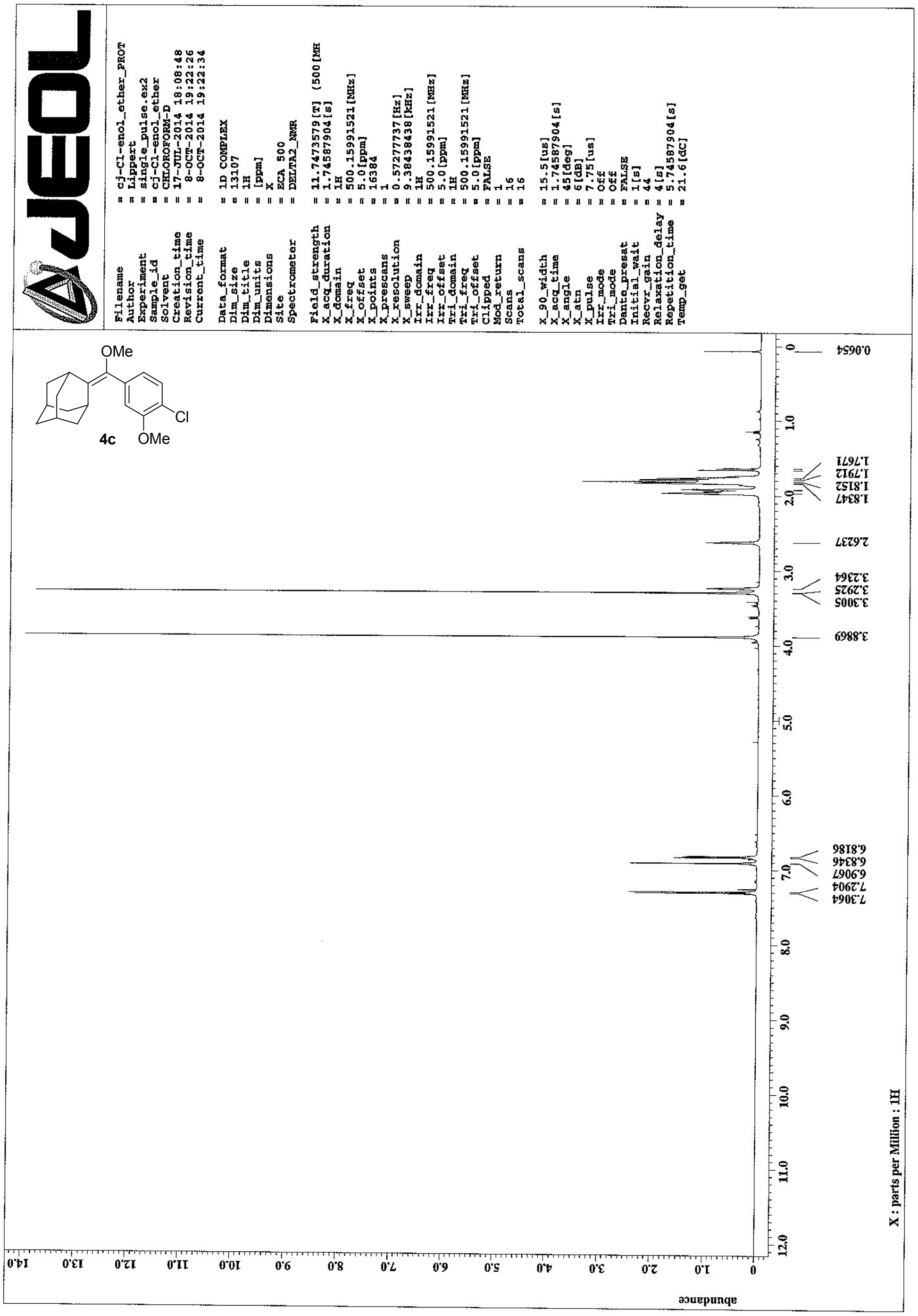
4c
OMe
Cl
OMe
Filename = cj-Cl-enol_ether_PROT
Author = Lippert
Experiment = single_pulse.ex2
Sample_id = cj-Cl-enol_ether
Solvent = CHLOROFORM-D
Creation_time = 17-JUL-2014 18:08:48
Revision_time = 8-OCT-2014 19:22:26
Current_time = 8-OCT-2014 19:22:34
Data_format = 1D COMPLEX
Dim_size = 13107
Dim_title = 1H
Dim_units = [ppm]
Dimensions = X
Site = ECA 500
Spectrometer = DELTA2_NMR
Field_strength = 11.7473579[T] (500[MH
X_acq_duration = 1.74587904[s]
X_domain = 1H
X_freq = 500.15991521[MHz]
X_offset = 5.0[ppm]
X_points = 16384
X_prescans = 1
X_resolution = 0.57277737[Hz]
X_sweep = 9.38438438[kHz]
Irr_domain = 1H
Irr_freq = 500.15991521[MHz]
Irr_offset = 5.0[ppm]
Tri_domain = 1H
Tri_freq = 500.15991521[MHz]
Tri_offset = 5.0[ppm]
Clipped = FALSE
Mod_return = 1
Scans = 16
Total_scans = 16
X_90_width = 15.5[us]
X_acq_time = 1.74587904[s]
X_angle = 45[deg]
X_atn = 6[dB]
X_pulse = 7.75[us]
Irr_mode = Off
Tri_mode = Off
Dante_presat = FALSE
Initial_wait = 1[s]
Recvr_gain = 44
Relaxation_delay = 4[s]
Repetition_time = 5.74587904[s]
Temp_get = 21.6[dC]
0.0654
1.7671
1.7912
1.8152
1.8347
2.6237
3.2364
3.2925
3.3005
3.8869
6.8186
6.8346
6.9067
7.2904
7.3064
X : parts per Million : 1H
abundance

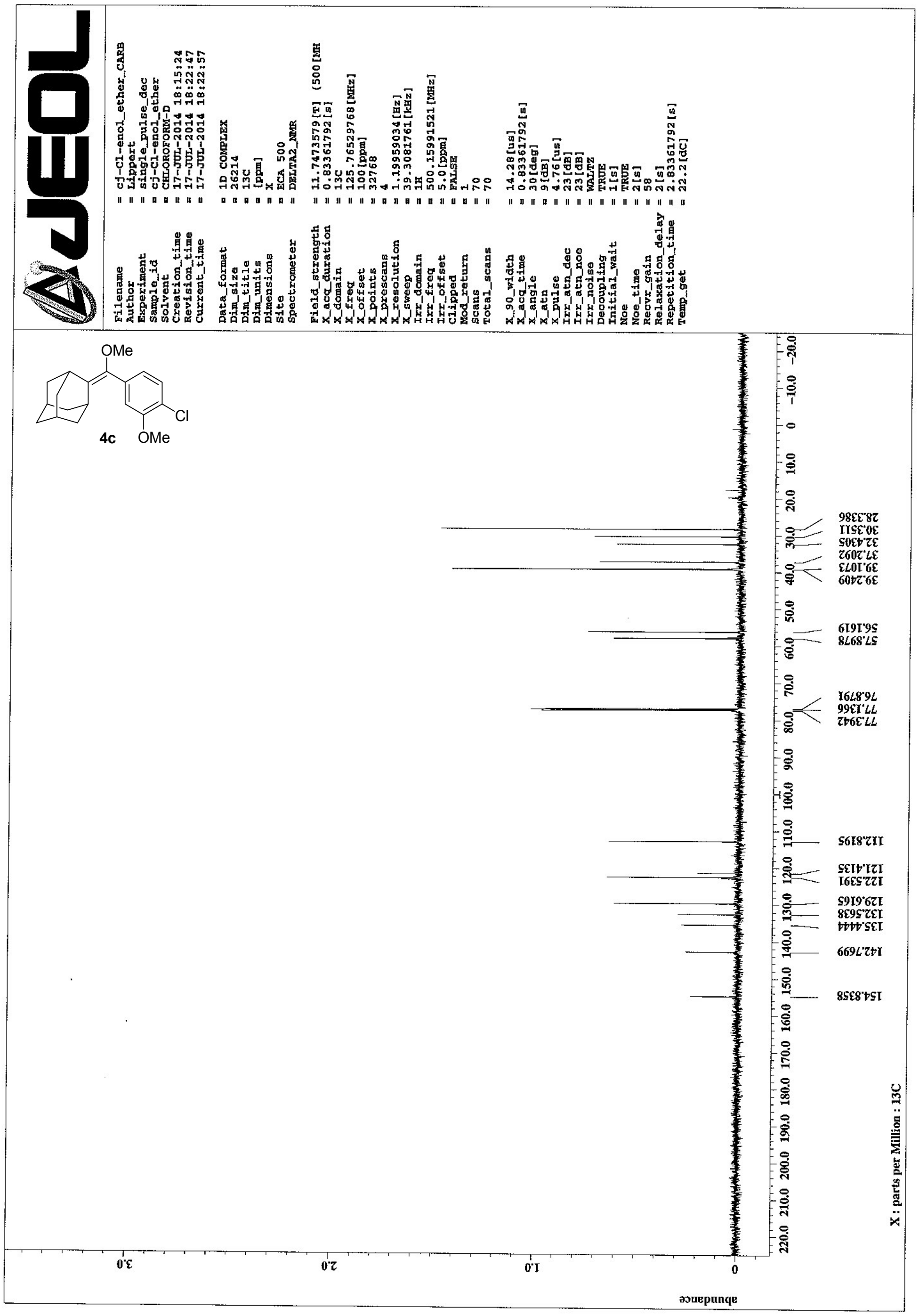
OMe
Cl
OMe
4c
JEOL
Filename = cj-Cl-enol_ether_CARB
Author = Lippert
Experiment = single_pulse_dec
Sample_id = cj-Cl-enol_ether
Solvent = CHLOROFORM-D
Creation_time = 17-JUL-2014 18:15:24
Revision_time = 17-JUL-2014 18:22:47
Current_time = 17-JUL-2014 18:22:57
Data_format = 1D COMPLEX
Dim_size = 26214
Dim_title = 13C
Dim_units = [ppm]
Dimensions = X
Site = ECA 500
Spectrometer = DELTA2_NMR
Field_strength = 11.7473579[T] (500[MH
X_acq_duration = 0.83361792[s]
X_domain = 13C
X_freq = 125.76529768[MHz]
X_offset = 100[ppm]
X_points = 32768
X_prescans = 4
X_resolution = 1.19959034[Hz]
X_sweep = 39.3081761[kHz]
Irr_domain = 1H
Irr_freq = 500.15991521[MHz]
Irr_offset = 5.0[ppm]
Clipped = FALSE
Mod_return = 1
Scans = 70
Total_scans = 70
X_90_width = 14.28[us]
X_acq_time = 0.83361792[s]
X_angle = 30[deg]
X_atn = 9[dB]
X_pulse = 4.76[us]
Irr_atn_dec = 23[dB]
Irr_atn_noe = 23[dB]
Irr_noise = WALTZ
Decoupling = TRUE
Initial_wait = 1[s]
Noe = TRUE
Noe_time = 2[s]
Recvr_gain = 58
Relaxation_delay = 2[s]
Repetition_time = 2.83361792[s]
Temp_get = 22.2[dC]
154.8358
142.7699
135.4444
132.5638
129.6165
122.5391
121.4135
112.8195
77.3942
77.1366
76.8791
57.8978
56.1619
39.2409
39.1073
37.2092
32.4305
30.3511
28.3386
abundance
X : parts per Million : 13C

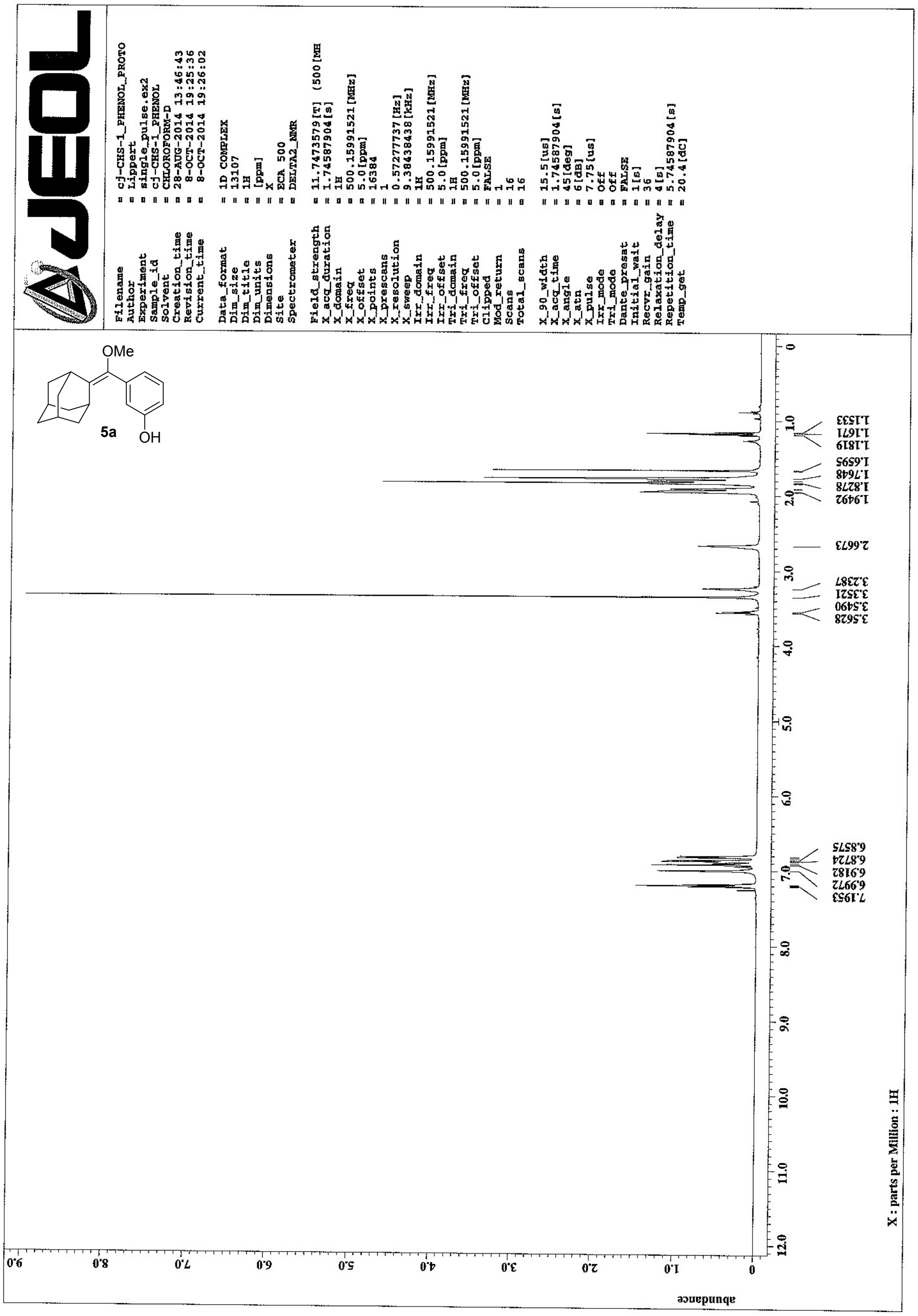

JEOL
OMe
5a
OH
Filename = cj-CHS-1_PHENOL_PROTO
Author = Lippert
Experiment = single_pulse.ex2
Sample_id = cj-CHS-1_PHENOL
Solvent = CHLOROFORM-D
Creation_time = 28-AUG-2014 13:46:43
Revision_time = 8-OCT-2014 19:25:36
Current_time = 8-OCT-2014 19:26:02
Data_format = 1D COMPLEX
Dim_size = 13107
Dim_title = 1H
Dim_units = [ppm]
Dimensions = X
Site = ECA 500
Spectrometer = DELTA2_NMR
Field_strength = 11.7473579[T] (500[MH
X_acq_duration = 1.74587904[s]
X_domain = 1H
X_freq = 500.15991521[MHz]
X_offset = 5.0[ppm]
X_points = 16384
X_prescans = 1
X_resolution = 0.57277737[Hz]
X_sweep = 9.38438438[kHz]
Irr_domain = 1H
Irr_freq = 500.15991521[MHz]
Irr_offset = 5.0[ppm]
Tri_domain = 1H
Tri_freq = 500.15991521[MHz]
Tri_offset = 5.0[ppm]
Clipped = FALSE
Mod_return = 1
Scans = 16
Total_scans = 16
X_90_width = 15.5[us]
X_acq_time = 1.74587904[s]
X_angle = 45[deg]
X_atn = 6[dB]
X_pulse = 7.75[us]
Irr_mode = Off
Tri_mode = Off
Dante_presat = FALSE
Initial_wait = 1[s]
Recvr_gain = 36
Relaxation_delay = 4[s]
Repetition_time = 5.74587904[s]
Temp_get = 20.4[dC]
1.1533
1.1671
1.1819
1.6595
1.7648
1.8278
1.9492
2.6673
3.2387
3.3521
3.5490
3.5628
6.8575
6.8724
6.9182
6.9972
7.1953
X : parts per Million : 1H
abundance

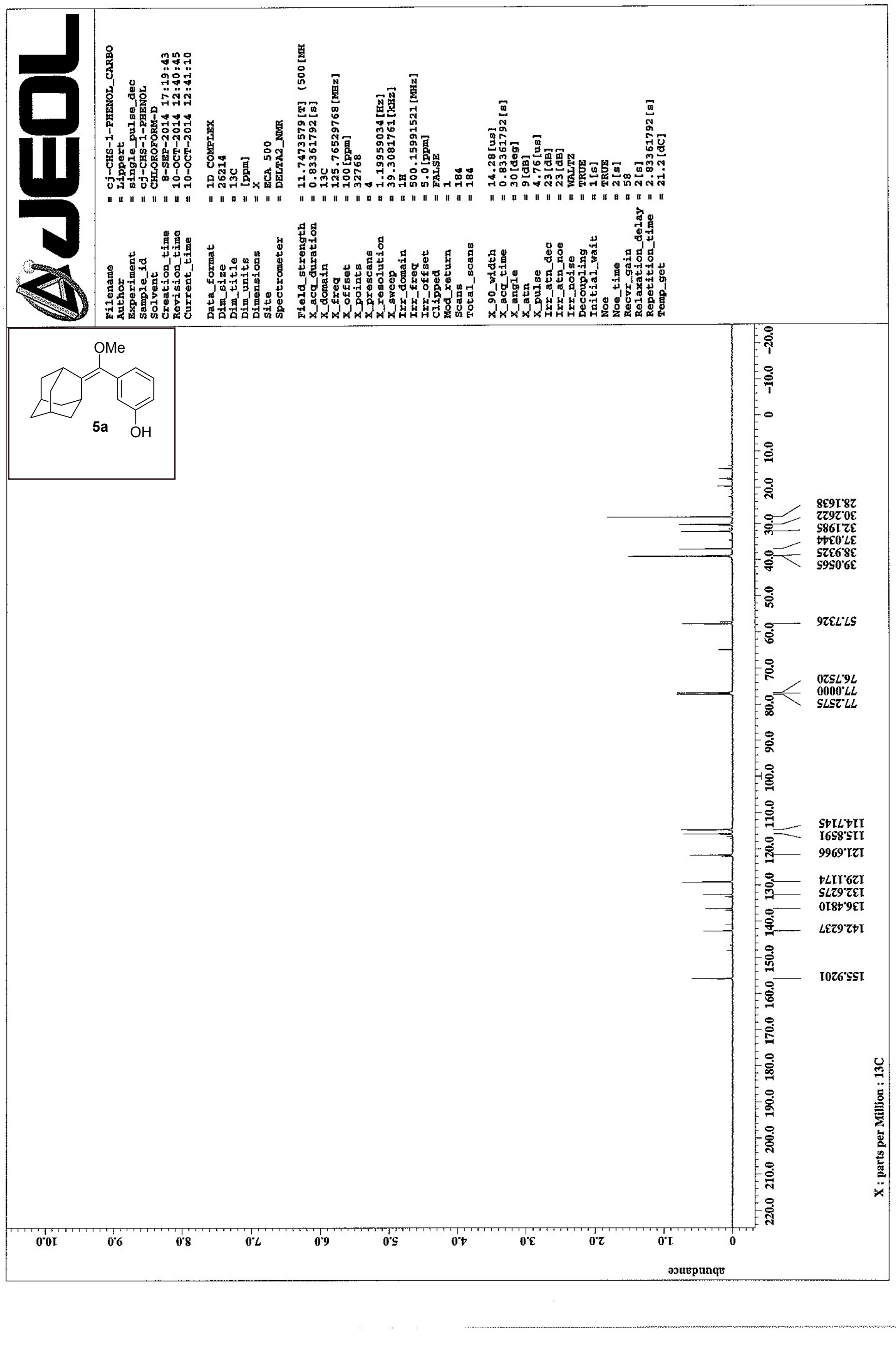
JEOL
5a
OMe
OH
Filename = cj-CHS-1-PHENOL_CARBO
Author = Lippert
Experiment = single_pulse_dec
Sample_id = cj-CHS-1-PHENOL
Solvent = CHLOROFORM-D
Creation_time = 8-SEP-2014 17:19:43
Revision_time = 10-OCT-2014 12:40:45
Current_time = 10-OCT-2014 12:41:10
Data_format = 1D COMPLEX
Dim_size = 26214
Dim_title = 13C
Dim_units = [ppm]
Dimensions = X
Site = ECA 500
Spectrometer = DELTA2_NMR
Field_strength = 11.7473579[T] (500[MH
X_acq_duration = 0.83361792[s]
X_domain = 13C
X_freq = 125.76529768[MHz]
X_offset = 100[ppm]
X_points = 32768
X_prescans = 4
X_resolution = 1.19959034[Hz]
X_sweep = 39.3081761[kHz]
Irr_domain = 1H
Irr_freq = 500.15991521[MHz]
Irr_offset = 5.0[ppm]
Clipped = FALSE
Mod_return = 1
Scans = 184
Total_scans = 184
X_90_width = 14.28[us]
X_acq_time = 0.83361792[s]
X_angle = 30[deg]
X_atn = 9[dB]
X_pulse = 4.76[us]
Irr_atn_dec = 23[dB]
Irr_atn_noe = 23[dB]
Irr_noise = WALTZ
Decoupling = TRUE
Initial_wait = 1[s]
Noe = TRUE
Noe_time = 2[s]
Recvr_gain = 58
Relaxation_delay = 2[s]
Repetition_time = 2.83361792[s]
Temp_get = 21.2[dC]
155.9201
142.6237
136.4810
132.6275
129.1174
121.6966
115.8591
114.7145
77.2575
77.0000
76.7520
57.7326
39.0565
38.9325
37.0344
32.1985
30.2622
28.1638
abundance
X : parts per Million : 13C

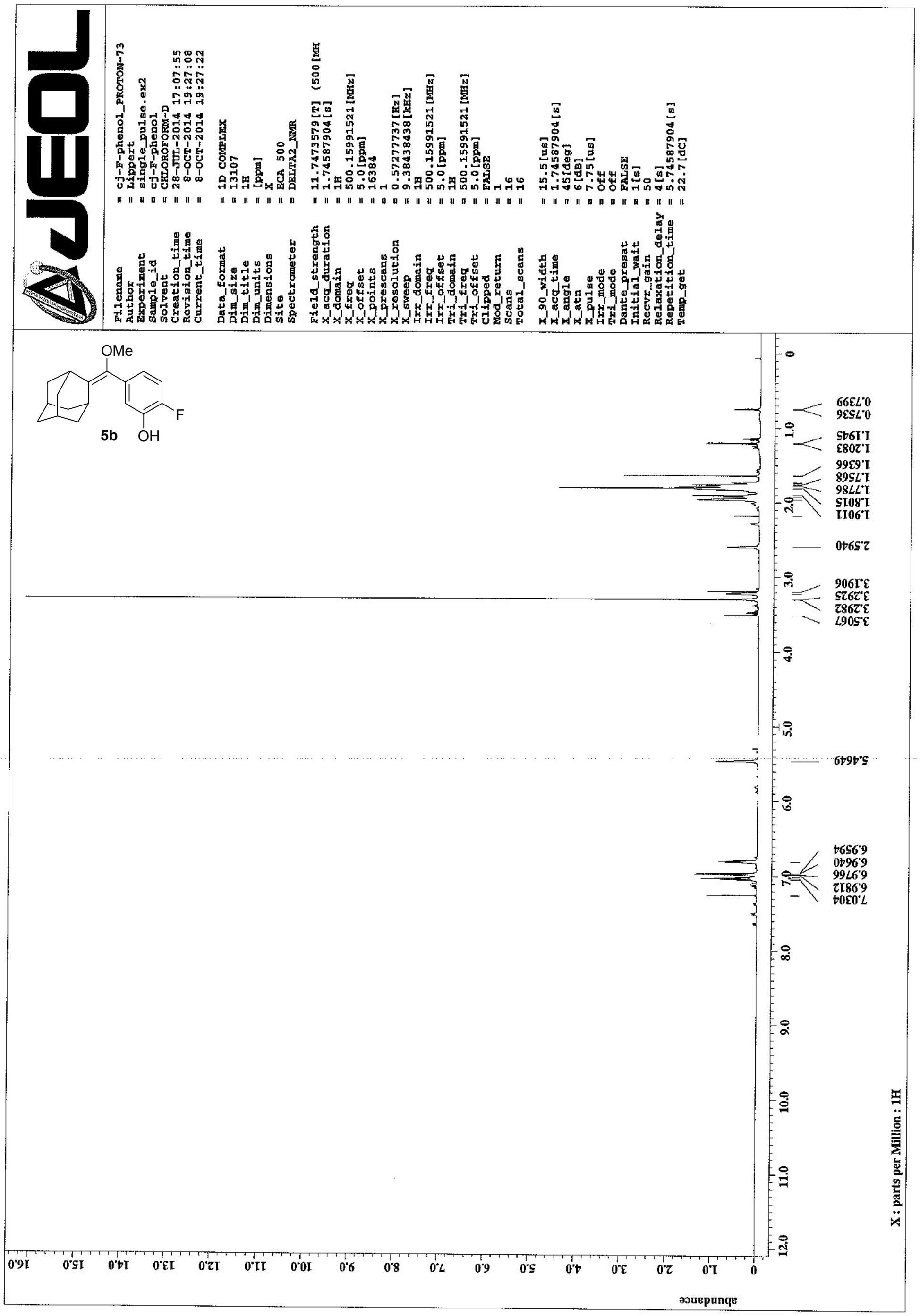
JEOL
Filename = cj-F-phenol_PROTON-73
Author = Lippert
Experiment = single_pulse.ex2
Sample_id = cj-F-phenol
Solvent = CHLOROFORM-D
Creation_time = 28-JUL-2014 17:07:55
Revision_time = 8-OCT-2014 19:27:08
Current_time = 8-OCT-2014 19:27:22
Data_format = 1D COMPLEX
Dim_size = 13107
Dim_title = 1H
Dim_units = [ppm]
Dimensions = X
Site = ECA 500
Spectrometer = DELTA2_NMR
Field_strength = 11.7473579[T] (500[MH
X_acq_duration = 1.74587904[s]
X_domain = 1H
X_freq = 500.15991521[MHz]
X_offset = 5.0[ppm]
X_points = 16384
X_prescans = 1
X_resolution = 0.57277737[Hz]
X_sweep = 9.38438438[kHz]
Irr_domain = 1H
Irr_freq = 500.15991521[MHz]
Irr_offset = 5.0[ppm]
Tri_domain = 1H
Tri_freq = 500.15991521[MHz]
Tri_offset = 5.0[ppm]
Clipped = FALSE
Mod_return = 1
Scans = 16
Total_scans = 16
X_90_width = 15.5[us]
X_acq_time = 1.74587904[s]
X_angle = 45[deg]
X_atn = 6[dB]
X_pulse = 7.75[us]
Irr_mode = Off
Tri_mode = Off
Dante_presat = FALSE
Initial_wait = 1[s]
Recvr_gain = 50
Relaxation_delay = 4[s]
Repetition_time = 5.74587904[s]
Temp_get = 22.7[dC]
OMe
F
OH
5b
7.0304
6.9812
6.9766
6.9640
6.9594
5.4649
3.5067
3.2982
3.2925
3.1906
2.5940
1.9011
1.8015
1.7786
1.7568
1.6366
1.2083
1.1945
0.7536
0.7399
X : parts per Million : 1H
abundance

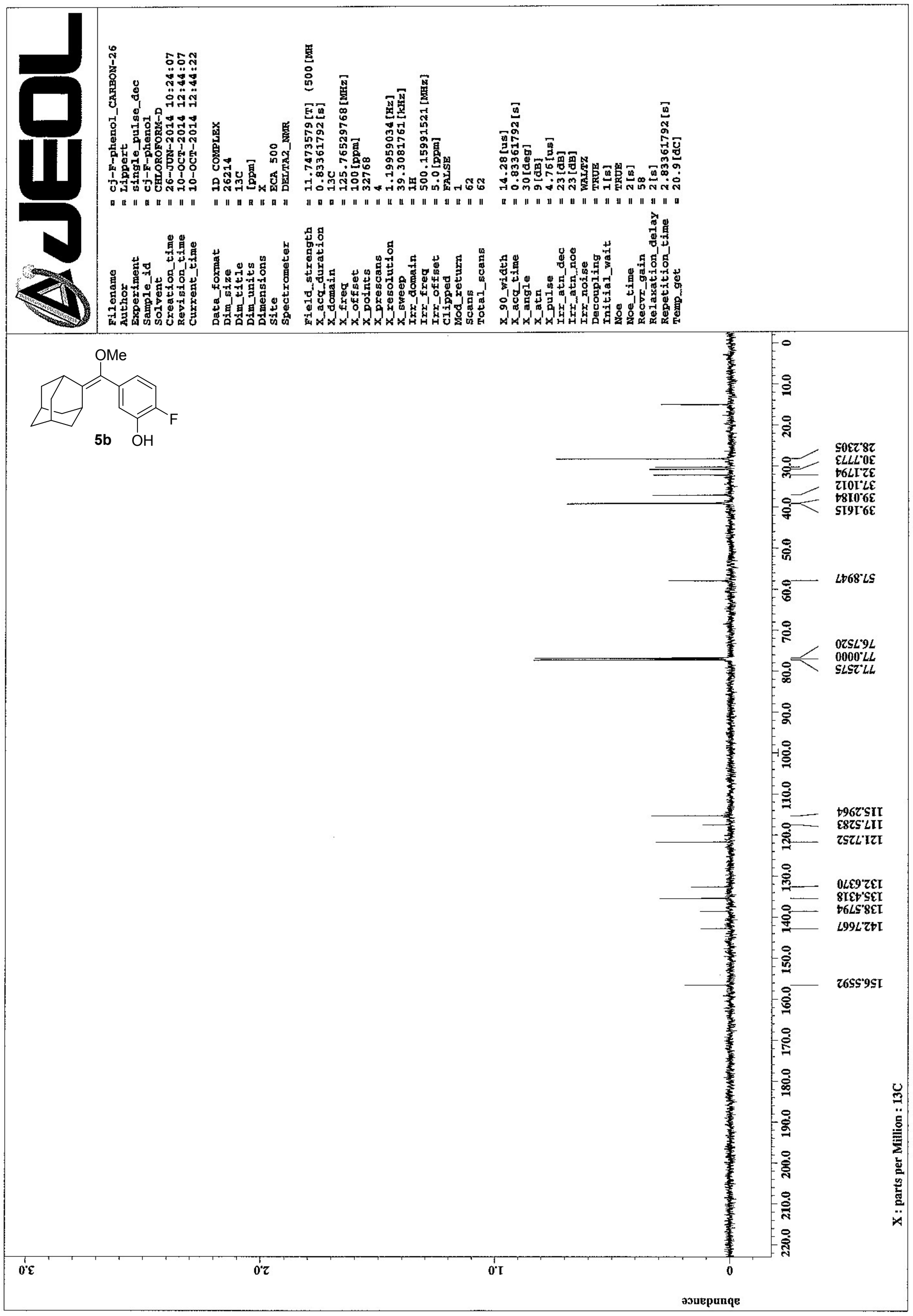
OMe
F
5b
OH
Filename = cj-F-phenol_CARBON-26
Author = Lippert
Experiment = single_pulse_dec
Sample_id = cj-F-phenol
Solvent = CHLOROFORM-D
Creation_time = 26-JUN-2014 10:24:07
Revision_time = 10-OCT-2014 12:44:07
Current_time = 10-OCT-2014 12:44:22
Data_format = 1D COMPLEX
Dim_size = 26214
Dim_title = 13C
Dim_units = [ppm]
Dimensions = X
Site = ECA 500
Spectrometer = DELTA2_NMR
Field_strength = 11.7473579[T] (500[MH
X_acq_duration = 0.83361792[s]
X_domain = 13C
X_freq = 125.76529768[MHz]
X_offset = 100[ppm]
X_points = 32768
X_prescans = 4
X_resolution = 1.19959034[Hz]
X_sweep = 39.3081761[kHz]
Irr_domain = 1H
Irr_freq = 500.15991521[MHz]
Irr_offset = 5.0[ppm]
Clipped = FALSE
Mod_return = 1
Scans = 62
Total_scans = 62
X_90_width = 14.28[us]
X_acq_time = 0.83361792[s]
X_angle = 30[deg]
X_atn = 9[dB]
X_pulse = 4.76[us]
Irr_atn_dec = 23[dB]
Irr_atn_noe = 23[dB]
Irr_noise = WALTZ
Decoupling = TRUE
Initial_wait = 1[s]
Noe = TRUE
Noe_time = 2[s]
Recvr_gain = 58
Relaxation_delay = 2[s]
Repetition_time = 2.83361792[s]
Temp_get = 20.9[dC]
156.5592
142.7667
138.5794
135.4318
132.6370
121.7252
117.5283
115.2964
77.2575
77.0000
76.7520
57.8947
39.1615
39.0184
37.1012
32.1794
30.7773
28.2305
abundance
X : parts per Million : 13C

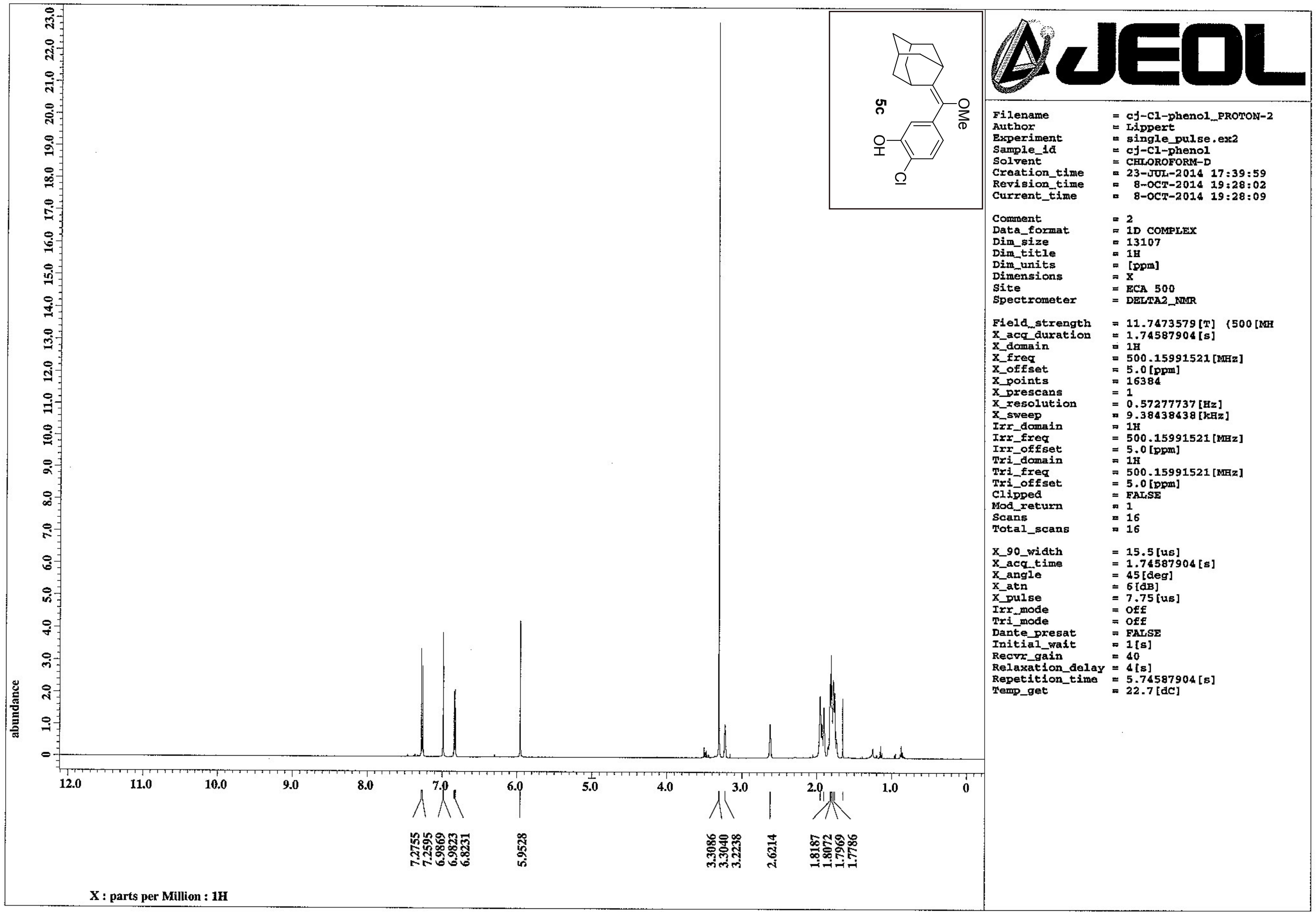
OMe
Cl
OH
5c
JEOL
Filename = cj-Cl-phenol_PROTON-2
Author = Lippert
Experiment = single_pulse.ex2
Sample_id = cj-Cl-phenol
Solvent = CHLOROFORM-D
Creation_time = 23-JUL-2014 17:39:59
Revision_time = 8-OCT-2014 19:28:02
Current_time = 8-OCT-2014 19:28:09
Comment = 2
Data_format = 1D COMPLEX
Dim_size = 13107
Dim_title = 1H
Dim_units = [ppm]
Dimensions = X
Site = ECA 500
Spectrometer = DELTA2_NMR
Field_strength = 11.7473579[T] (500[MH
X_acq_duration = 1.74587904[s]
X_domain = 1H
X_freq = 500.15991521[MHz]
X_offset = 5.0[ppm]
X_points = 16384
X_prescans = 1
X_resolution = 0.57277737[Hz]
X_sweep = 9.38438438[kHz]
Irr_domain = 1H
Irr_freq = 500.15991521[MHz]
Irr_offset = 5.0[ppm]
Tri_domain = 1H
Tri_freq = 500.15991521[MHz]
Tri_offset = 5.0[ppm]
Clipped = FALSE
Mod_return = 1
Scans = 16
Total_scans = 16
X_90_width = 15.5[us]
X_acq_time = 1.74587904[s]
X_angle = 45[deg]
X_atn = 6[dB]
X_pulse = 7.75[us]
Irr_mode = Off
Tri_mode = Off
Dante_presat = FALSE
Initial_wait = 1[s]
Recvr_gain = 40
Relaxation_delay = 4[s]
Repetition_time = 5.74587904[s]
Temp_get = 22.7[dC]
abundance
7.2755
7.2595
6.9869
6.9823
6.8231
5.9528
3.3086
3.3040
3.2238
2.6214
1.8187
1.8072
1.7969
1.7786
X : parts per Million : 1H

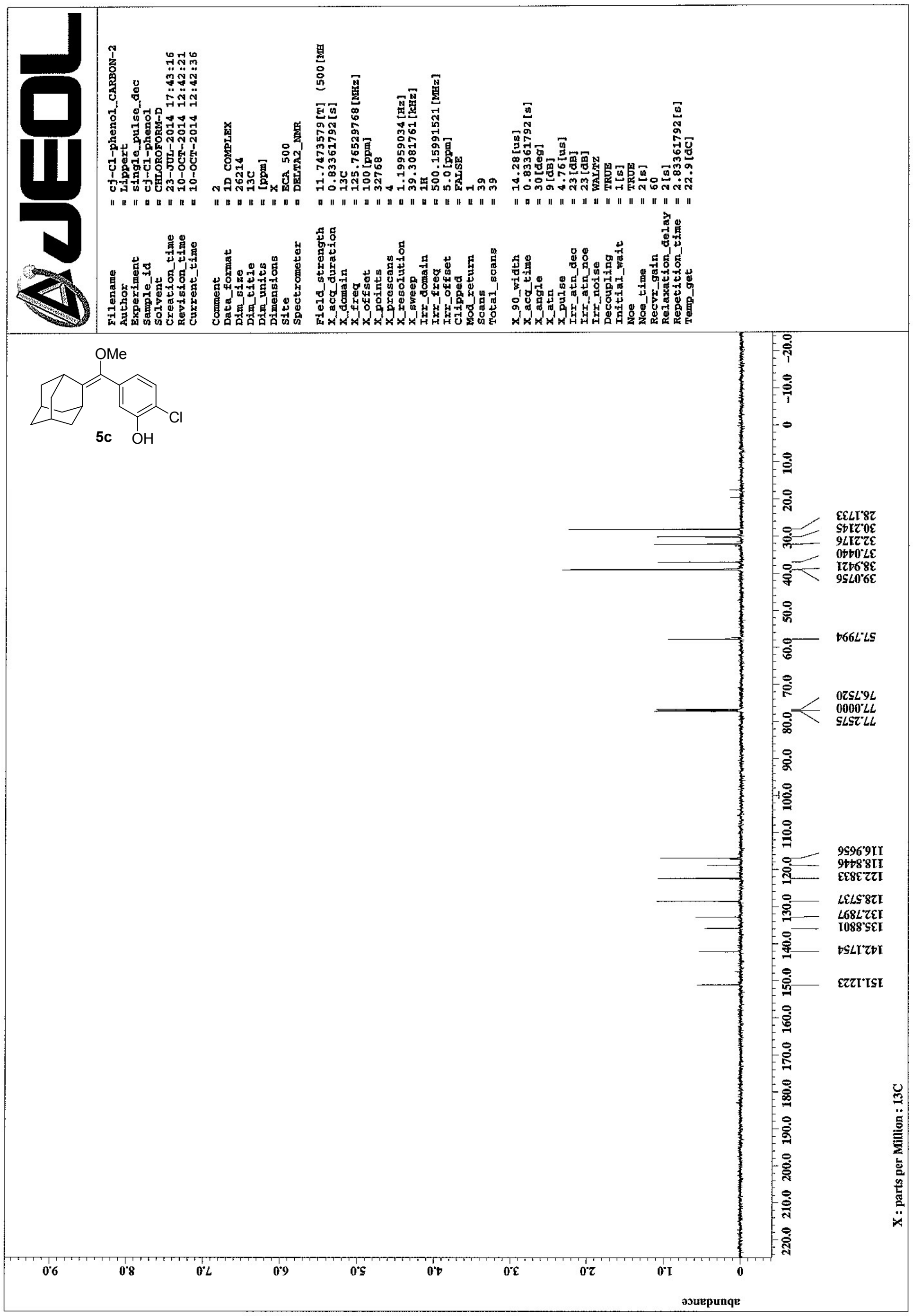

OMe
5c
OH
Cl
Filename = cj-Cl-phenol_CARBON-2
Author = Lippert
Experiment = single_pulse_dec
Sample_id = cj-Cl-phenol
Solvent = CHLOROFORM-D
Creation_time = 23-JUL-2014 17:43:16
Revision_time = 10-OCT-2014 12:42:21
Current_time = 10-OCT-2014 12:42:36
Comment = 2
Data_format = 1D COMPLEX
Dim_size = 26214
Dim_title = 13C
Dim_units = [ppm]
Dimensions = X
Site = ECA 500
Spectrometer = DELTA2_NMR
Field_strength = 11.7473579[T] (500[MH
X_acq_duration = 0.83361792[s]
X_domain = 13C
X_freq = 125.76529768[MHz]
X_offset = 100[ppm]
X_points = 32768
X_prescans = 4
X_resolution = 1.19959034[Hz]
X_sweep = 39.3081761[kHz]
Irr_domain = 1H
Irr_freq = 500.15991521[MHz]
Irr_offset = 5.0[ppm]
Clipped = FALSE
Mod_return = 1
Scans = 39
Total_scans = 39
X_90_width = 14.28[us]
X_acq_time = 0.83361792[s]
X_angle = 30[deg]
X_atn = 9[dB]
X_pulse = 4.76[us]
Irr_atn_dec = 23[dB]
Irr_atn_noe = 23[dB]
Irr_noise = WALTZ
Decoupling = TRUE
Initial_wait = 1[s]
Noe = TRUE
Noe_time = 2[s]
Recvr_gain = 60
Relaxation_delay = 2[s]
Repetition_time = 2.83361792[s]
Temp_get = 22.9[dC]
151.1223
142.1754
135.8801
132.7897
128.5737
122.3833
118.8446
116.9656
77.2575
77.0000
76.7520
57.7994
39.0756
38.9421
37.0440
32.2176
30.2145
28.1733
abundance
X : parts per Million : 13C

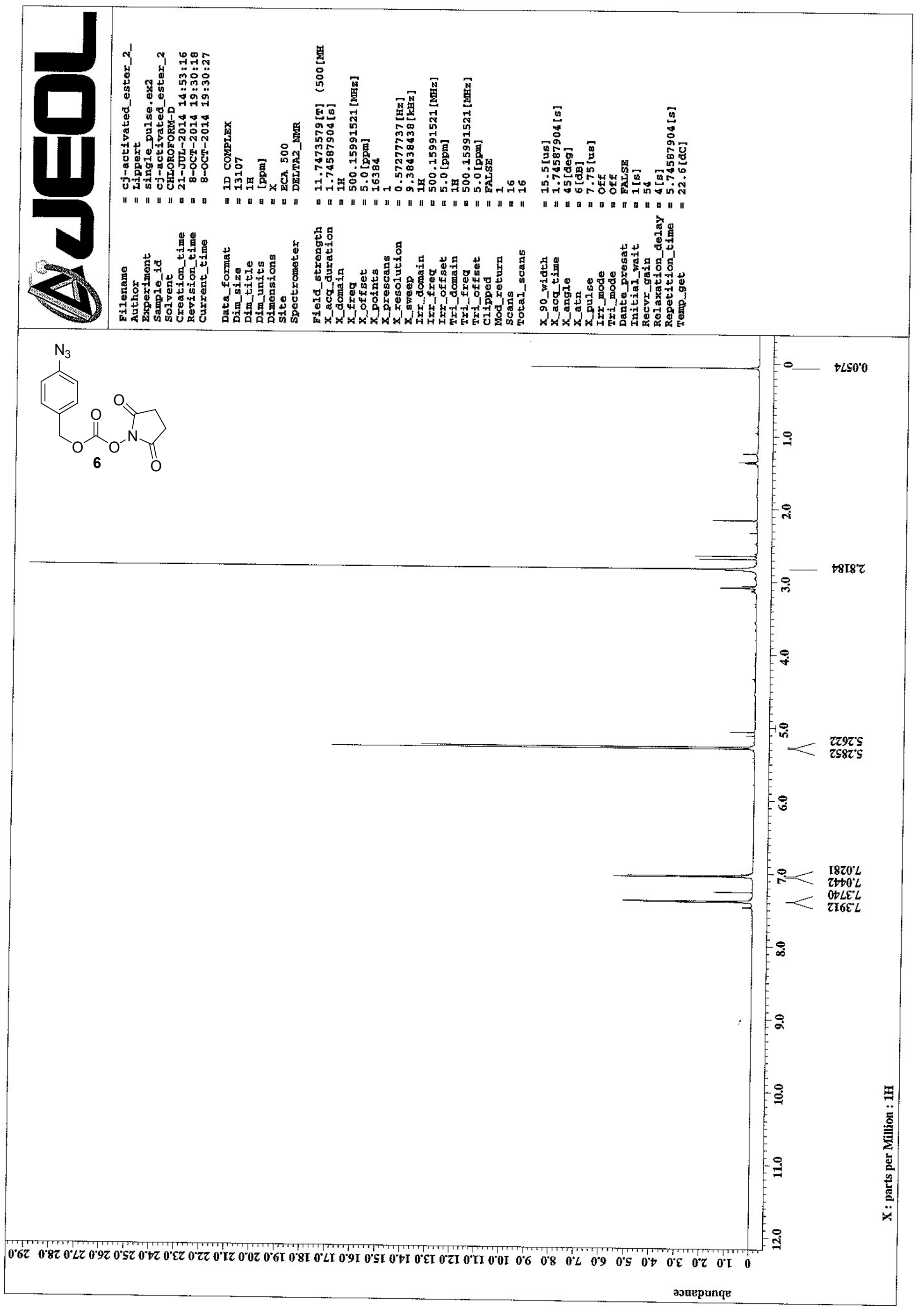
6
0.0574
2.8184
5.2622
5.2852
7.0281
7.0442
7.3740
7.3912
X : parts per Million : 1H
abundance

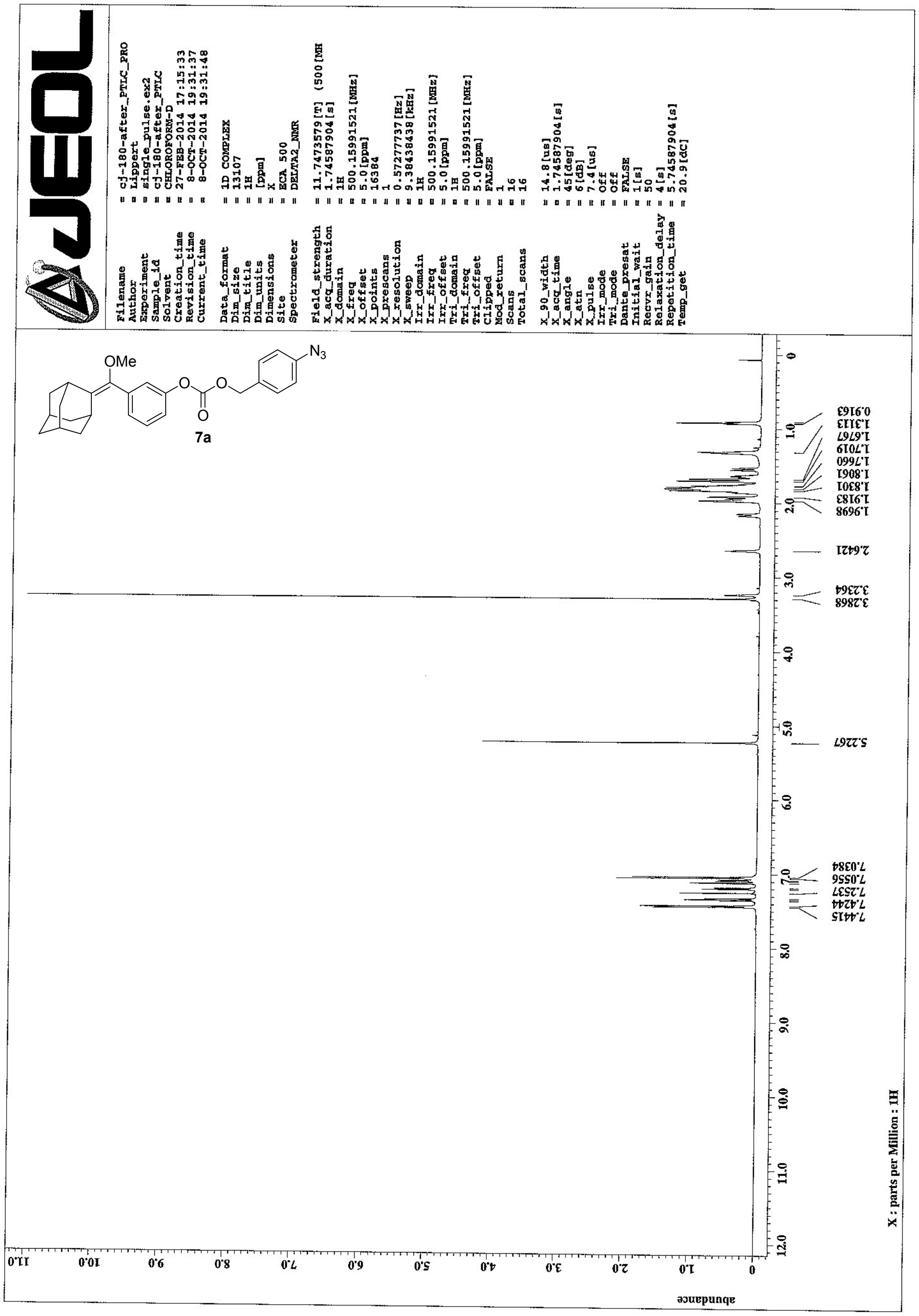
OMe
N3
O
7a
JEOL
Filename = cj-180-after_PTLC_PRO
Author = Lippert
Experiment = single_pulse.ex2
Sample_id = cj-180-after_PTLC
Solvent = CHLOROFORM-D
Creation_time = 27-FEB-2014 17:15:33
Revision_time = 8-OCT-2014 19:31:37
Current_time = 8-OCT-2014 19:31:48
Data_format = 1D COMPLEX
Dim_size = 13107
Dim_title = 1H
Dim_units = [ppm]
Dimensions = X
Site = ECA 500
Spectrometer = DELTA2_NMR
Field_strength = 11.7473579[T] (500[MH
X_acq_duration = 1.74587904[s]
X_domain = 1H
X_freq = 500.15991521[MHz]
X_offset = 5.0[ppm]
X_points = 16384
X_prescans = 1
X_resolution = 0.57277737[Hz]
X_sweep = 9.38438438[kHz]
Irr_domain = 1H
Irr_freq = 500.15991521[MHz]
Irr_offset = 5.0[ppm]
Tri_domain = 1H
Tri_freq = 500.15991521[MHz]
Tri_offset = 5.0[ppm]
Clipped = FALSE
Mod_return = 1
Scans = 16
Total_scans = 16
X_90_width = 14.8[us]
X_acq_time = 1.74587904[s]
X_angle = 45[deg]
X_atn = 6[dB]
X_pulse = 7.4[us]
Irr_mode = Off
Tri_mode = Off
Dante_presat = FALSE
Initial_wait = 1[s]
Recvr_gain = 50
Relaxation_delay = 4[s]
Repetition_time = 5.74587904[s]
Temp_get = 20.9[dC]
abundance
0 1.0 2.0 3.0 4.0 5.0 6.0 7.0 8.0 9.0 10.0 11.0
12.0 11.0 10.0 9.0 8.0 7.0 6.0 5.0 4.0 3.0 2.0 1.0 0
7.4415
7.4244
7.2537
7.0556
7.0384
5.2267
3.2868
3.2364
2.6421
1.9698
1.9183
1.8301
1.8061
1.7660
1.7019
1.6767
1.3113
0.9163
X : parts per Million : 1H

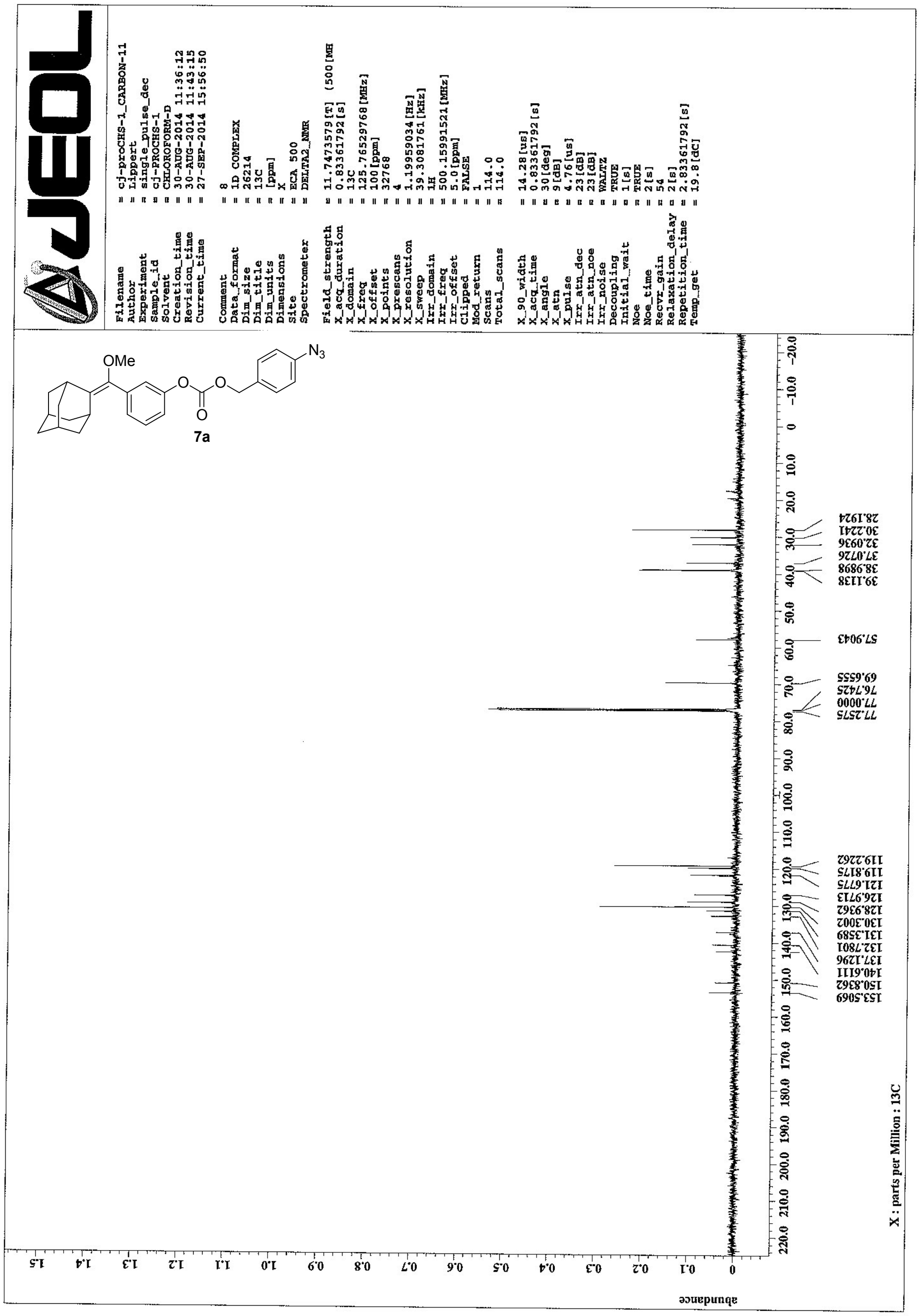
OMe
N3
7a
JEOL
Filename = cj-proCHS-1_CARBON-11
Author = Lippert
Experiment = single_pulse_dec
Sample_id = cj-PROCHS-1
Solvent = CHLOROFORM-D
Creation_time = 30-AUG-2014 11:36:12
Revision_time = 30-AUG-2014 11:43:15
Current_time = 27-SEP-2014 15:56:50
Comment = 8
Data_format = 1D COMPLEX
Dim_size = 26214
Dim_title = 13C
Dim_units = [ppm]
Dimensions = X
Site = ECA 500
Spectrometer = DELTA2_NMR
Field_strength = 11.7473579[T] (500[MH
X_acq_duration = 0.83361792[s]
X_domain = 13C
X_freq = 125.76529768[MHz]
X_offset = 100[ppm]
X_points = 32768
X_prescans = 4
X_resolution = 1.19959034[Hz]
X_sweep = 39.3081761[kHz]
Irr_domain = 1H
Irr_freq = 500.15991521[MHz]
Irr_offset = 5.0[ppm]
Clipped = FALSE
Mod_return = 1
Scans = 114.0
Total_scans = 114.0
X_90_width = 14.28[us]
X_acq_time = 0.83361792[s]
X_angle = 30[deg]
X_atn = 9[dB]
X_pulse = 4.76[us]
Irr_atn_dec = 23[dB]
Irr_atn_noe = 23[dB]
Irr_noise = WALTZ
Decoupling = TRUE
Initial_wait = 1[s]
Noe = TRUE
Noe_time = 2[s]
Recvr_gain = 54
Relaxation_delay = 2[s]
Repetition_time = 2.83361792[s]
Temp_get = 19.8[dC]
153.5069
150.8362
140.6111
137.1296
132.7801
131.3589
130.3002
128.9362
126.9713
121.6775
119.8175
119.2262
77.2575
77.0000
76.7425
69.6555
57.9043
39.1138
38.9898
37.0726
32.0936
30.2241
28.1924
abundance
X : parts per Million : 13C

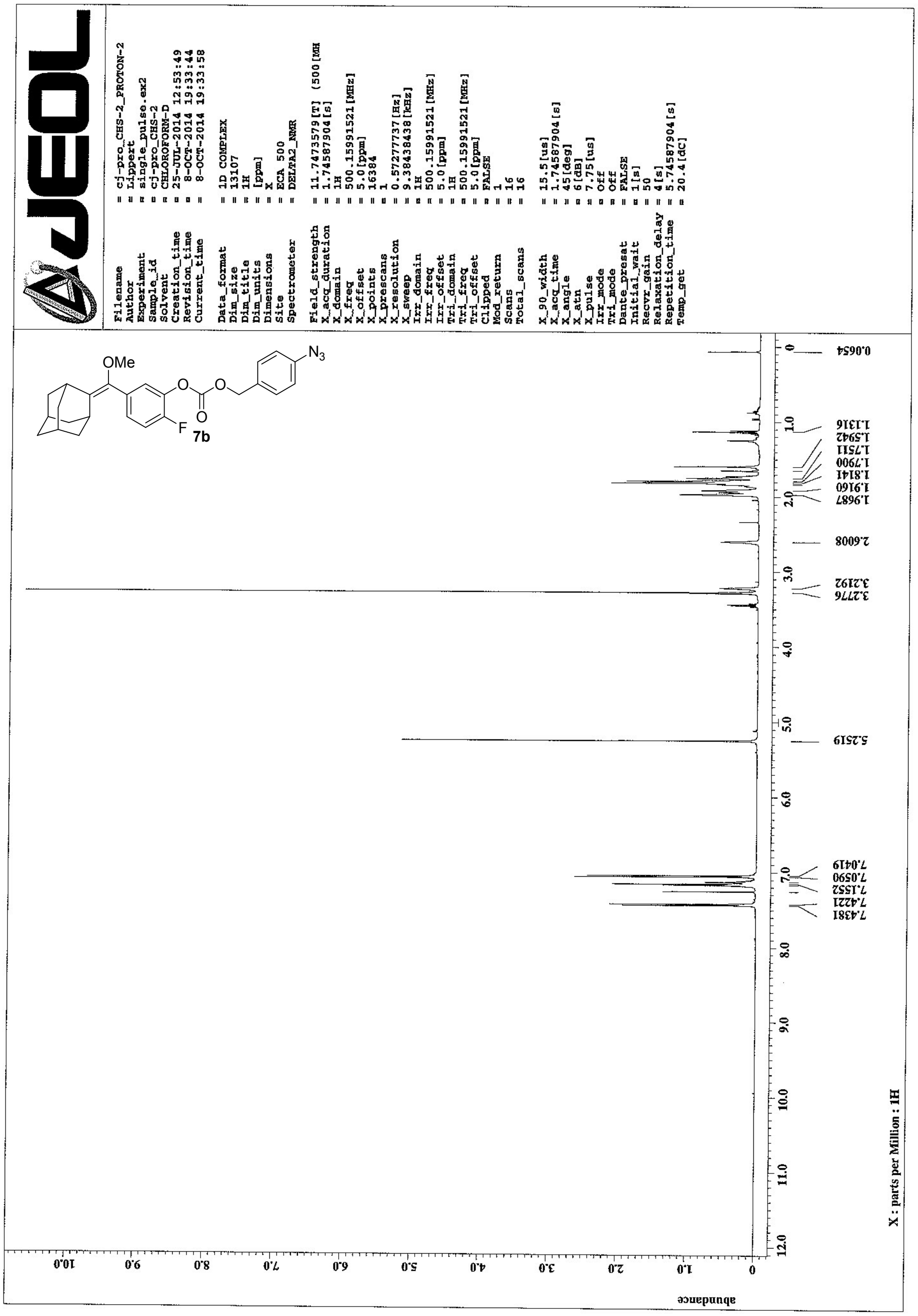
JEOL
Filename = cj-pro_CHS-2_PROTON-2
Author = Lippert
Experiment = single_pulse.ex2
Sample_id = cj-pro_CHS-2
Solvent = CHLOROFORM-D
Creation_time = 25-JUL-2014 12:53:49
Revision_time = 8-OCT-2014 19:33:44
Current_time = 8-OCT-2014 19:33:58
Data_format = 1D COMPLEX
Dim_size = 13107
Dim_title = 1H
Dim_units = [ppm]
Dimensions = X
Site = ECA 500
Spectrometer = DELTA2_NMR
Field_strength = 11.7473579[T] (500[MH
X_acq_duration = 1.74587904[s]
X_domain = 1H
X_freq = 500.15991521[MHz]
X_offset = 5.0[ppm]
X_points = 16384
X_prescans = 1
X_resolution = 0.57277737[Hz]
X_sweep = 9.38438438[kHz]
Irr_domain = 1H
Irr_freq = 500.15991521[MHz]
Irr_offset = 5.0[ppm]
Tri_domain = 1H
Tri_freq = 500.15991521[MHz]
Tri_offset = 5.0[ppm]
Clipped = FALSE
Mod_return = 1
Scans = 16
Total_scans = 16
X_90_width = 15.5[us]
X_acq_time = 1.74587904[s]
X_angle = 45[deg]
X_atn = 6[dB]
X_pulse = 7.75[us]
Irr_mode = Off
Tri_mode = Off
Dante_presat = FALSE
Initial_wait = 1[s]
Recvr_gain = 50
Relaxation_delay = 4[s]
Repetition_time = 5.74587904[s]
Temp_get = 20.4[dC]
OMe
N3
F
7b
0.0654
1.1316
1.5942
1.7511
1.7900
1.8141
1.9160
1.9687
2.6008
3.2192
3.2776
5.2519
7.0419
7.0590
7.1552
7.4221
7.4381
X : parts per Million : 1H
abundance

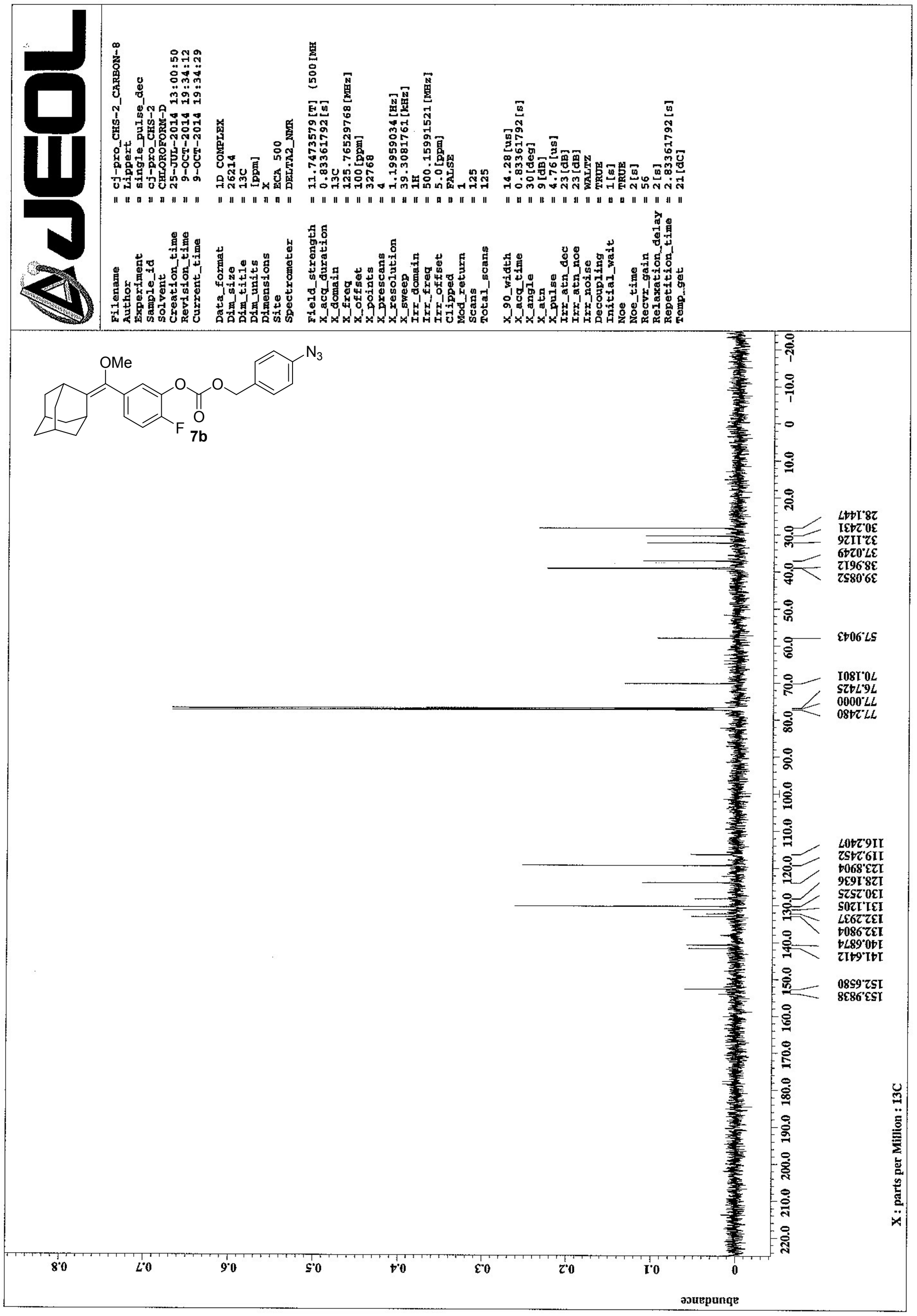

OMe
N3
O
F
7b
JEOL
Filename = cj-pro_CHS-2_CARBON-8
Author = Lippert
Experiment = single_pulse_dec
Sample_id = cj-pro_CHS-2
Solvent = CHLOROFORM-D
Creation_time = 25-JUL-2014 13:00:50
Revision_time = 9-OCT-2014 19:34:12
Current_time = 9-OCT-2014 19:34:29
Data_format = 1D COMPLEX
Dim_size = 26214
Dim_title = 13C
Dim_units = [ppm]
Dimensions = X
Site = ECA 500
Spectrometer = DELTA2_NMR
Field_strength = 11.7473579[T] (500[MH
X_acq_duration = 0.83361792[s]
X_domain = 13C
X_freq = 125.76529768[MHz]
X_offset = 100[ppm]
X_points = 32768
X_prescans = 4
X_resolution = 1.19959034[Hz]
X_sweep = 39.3081761[kHz]
Irr_domain = 1H
Irr_freq = 500.15991521[MHz]
Irr_offset = 5.0[ppm]
Clipped = FALSE
Mod_return = 1
Scans = 125
Total_scans = 125
X_90_width = 14.28[us]
X_acq_time = 0.83361792[s]
X_angle = 30[deg]
X_atn = 9[dB]
X_pulse = 4.76[us]
Irr_atn_dec = 23[dB]
Irr_atn_noe = 23[dB]
Irr_noise = WALTZ
Decoupling = TRUE
Initial_wait = 1[s]
Noe = TRUE
Noe_time = 2[s]
Recvr_gain = 56
Relaxation_delay = 2[s]
Repetition_time = 2.83361792[s]
Temp_get = 21[dC]
28.1447
30.2431
32.1126
37.0249
38.9612
39.0852
57.9043
70.1801
76.7425
77.0000
77.2480
116.2407
119.2452
123.8904
128.1636
130.2525
131.1205
132.2937
132.9804
140.6874
141.6412
152.6580
153.9838
X : parts per Million : 13C
abundance

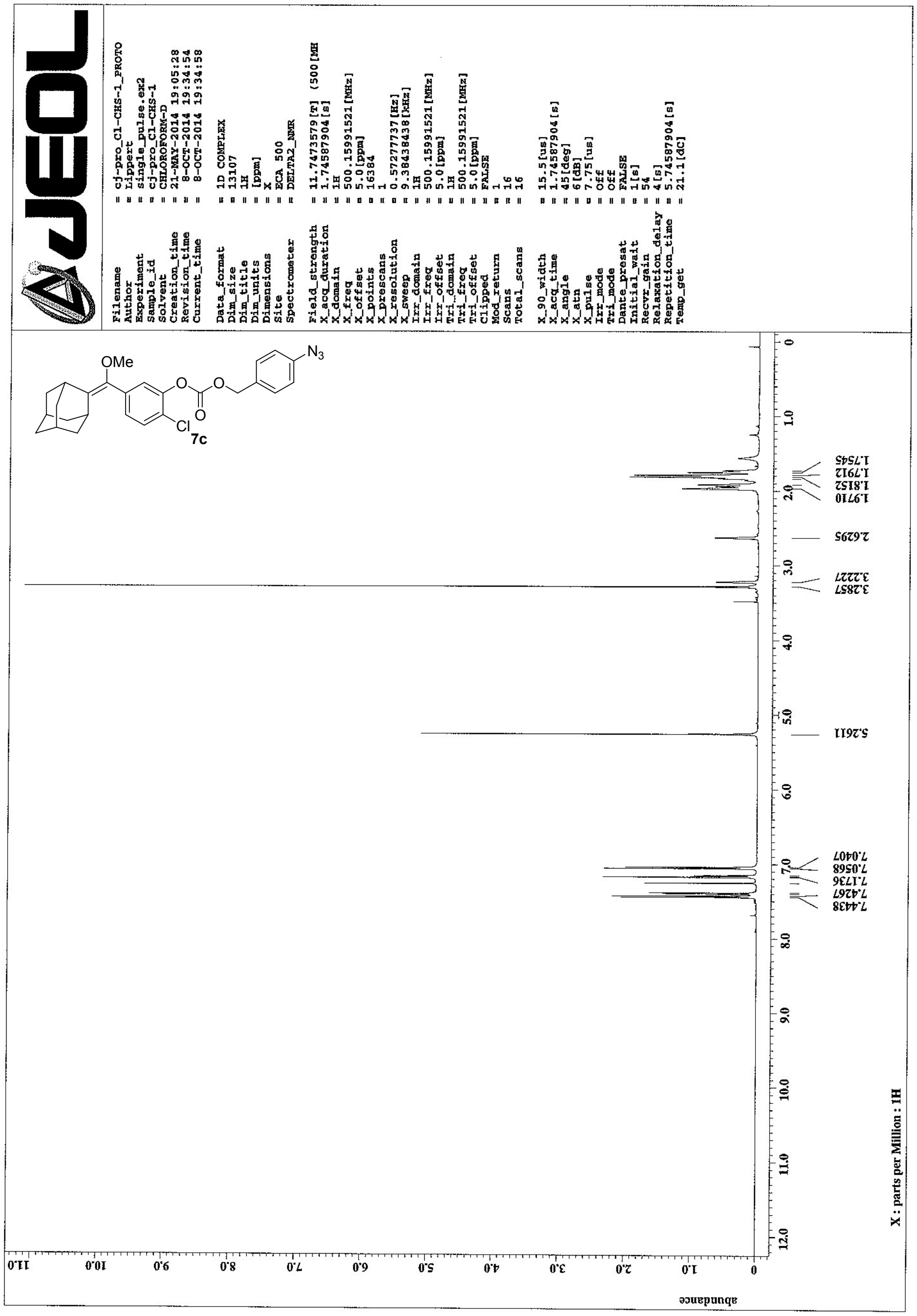

OMe
Cl
7c
N3
Filename = cj-pro_Cl-CHS-1_PROTO
Author = Lippert
Experiment = single_pulse.ex2
Sample_id = cj-pro_Cl-CHS-1
Solvent = CHLOROFORM-D
Creation_time = 21-MAY-2014 19:05:28
Revision_time = 8-OCT-2014 19:34:54
Current_time = 8-OCT-2014 19:34:58
Data_format = 1D COMPLEX
Dim_size = 13107
Dim_title = 1H
Dim_units = [ppm]
Dimensions = X
Site = ECA 500
Spectrometer = DELTA2_NMR
Field_strength = 11.7473579[T] (500[MH
X_acq_duration = 1.74587904[s]
X_domain = 1H
X_freq = 500.15991521[MHz]
X_offset = 5.0[ppm]
X_points = 16384
X_prescans = 1
X_resolution = 0.57277737[Hz]
X_sweep = 9.38438438[kHz]
Irr_domain = 1H
Irr_freq = 500.15991521[MHz]
Irr_offset = 5.0[ppm]
Tri_domain = 1H
Tri_freq = 500.15991521[MHz]
Tri_offset = 5.0[ppm]
Clipped = FALSE
Mod_return = 1
Scans = 16
Total_scans = 16
X_90_width = 15.5[us]
X_acq_time = 1.74587904[s]
X_angle = 45[deg]
X_atn = 6[dB]
X_pulse = 7.75[us]
Irr_mode = Off
Tri_mode = Off
Dante_presat = FALSE
Initial_wait = 1[s]
Recvr_gain = 54
Relaxation_delay = 4[s]
Repetition_time = 5.74587904[s]
Temp_get = 21.1[dC]
7.4438
7.4267
7.1736
7.0568
7.0407
5.2611
3.2857
3.2227
2.6295
1.9710
1.8152
1.7912
1.7545
abundance
X : parts per Million : 1H

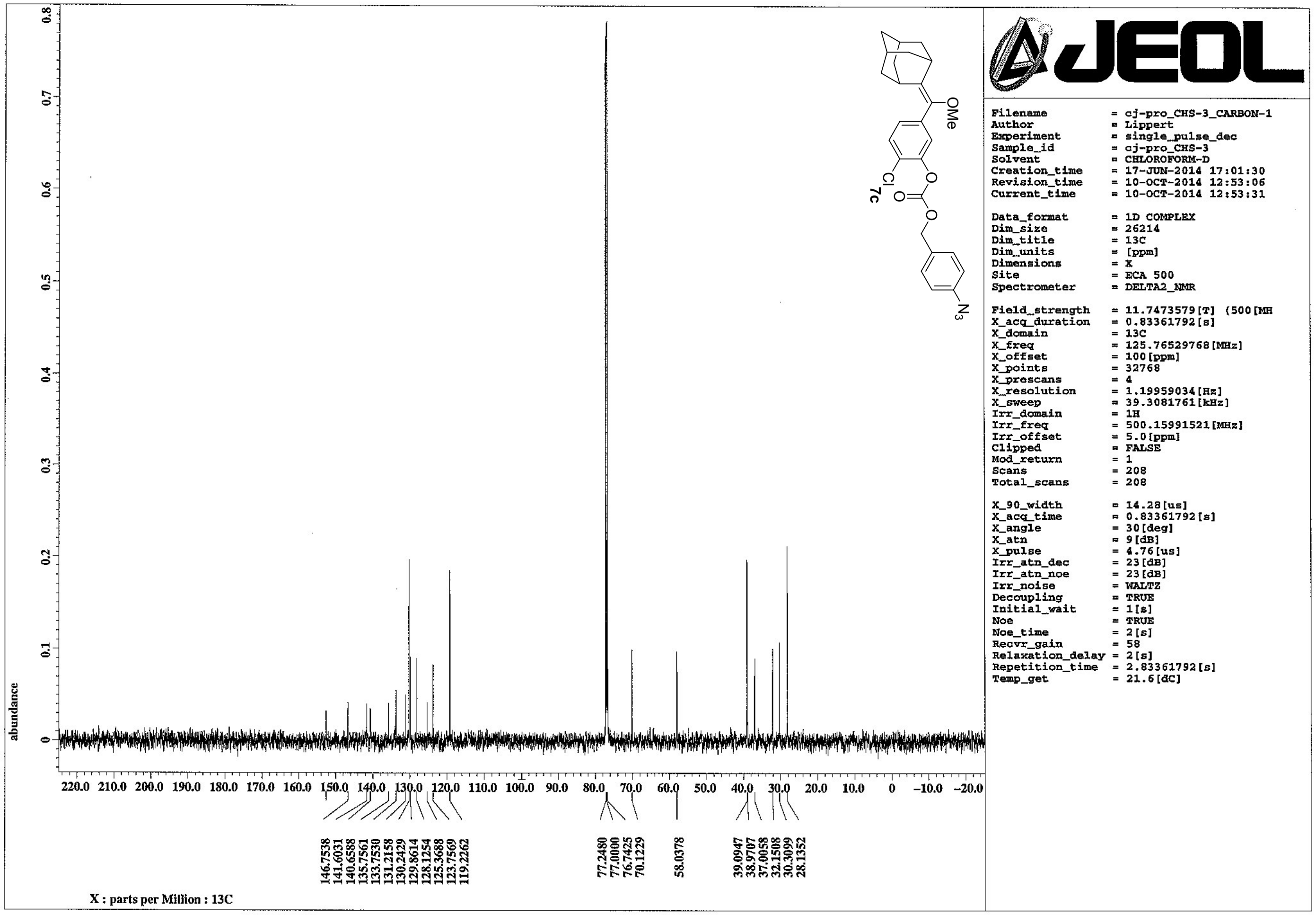
OMe
Cl
7c
N3
JEOL
Filename = cj-pro_CHS-3_CARBON-1
Author = Lippert
Experiment = single_pulse_dec
Sample_id = cj-pro_CHS-3
Solvent = CHLOROFORM-D
Creation_time = 17-JUN-2014 17:01:30
Revision_time = 10-OCT-2014 12:53:06
Current_time = 10-OCT-2014 12:53:31
Data_format = 1D COMPLEX
Dim_size = 26214
Dim_title = 13C
Dim_units = [ppm]
Dimensions = X
Site = ECA 500
Spectrometer = DELTA2_NMR
Field_strength = 11.7473579[T] (500[MH
X_acq_duration = 0.83361792[s]
X_domain = 13C
X_freq = 125.76529768[MHz]
X_offset = 100[ppm]
X_points = 32768
X_prescans = 4
X_resolution = 1.19959034[Hz]
X_sweep = 39.3081761[kHz]
Irr_domain = 1H
Irr_freq = 500.15991521[MHz]
Irr_offset = 5.0[ppm]
Clipped = FALSE
Mod_return = 1
Scans = 208
Total_scans = 208
X_90_width = 14.28[us]
X_acq_time = 0.83361792[s]
X_angle = 30[deg]
X_atn = 9[dB]
X_pulse = 4.76[us]
Irr_atn_dec = 23[dB]
Irr_atn_noe = 23[dB]
Irr_noise = WALTZ
Decoupling = TRUE
Initial_wait = 1[s]
Noe = TRUE
Noe_time = 2[s]
Recvr_gain = 58
Relaxation_delay = 2[s]
Repetition_time = 2.83361792[s]
Temp_get = 21.6[dC]
abundance
0 0.1 0.2 0.3 0.4 0.5 0.6 0.7 0.8
220.0 210.0 200.0 190.0 180.0 170.0 160.0 150.0 140.0 130.0 120.0 110.0 100.0 90.0 80.0 70.0 60.0 50.0 40.0 30.0 20.0 10.0 0 -10.0 -20.0
146.7538
141.6031
140.6588
135.7561
133.7530
131.2158
130.2429
129.8614
128.1254
125.3688
123.7569
119.2262
77.2480
77.0000
76.7425
70.1229
58.0378
39.0947
38.9707
37.0058
32.1508
30.3099
28.1352
X : parts per Million : 13C

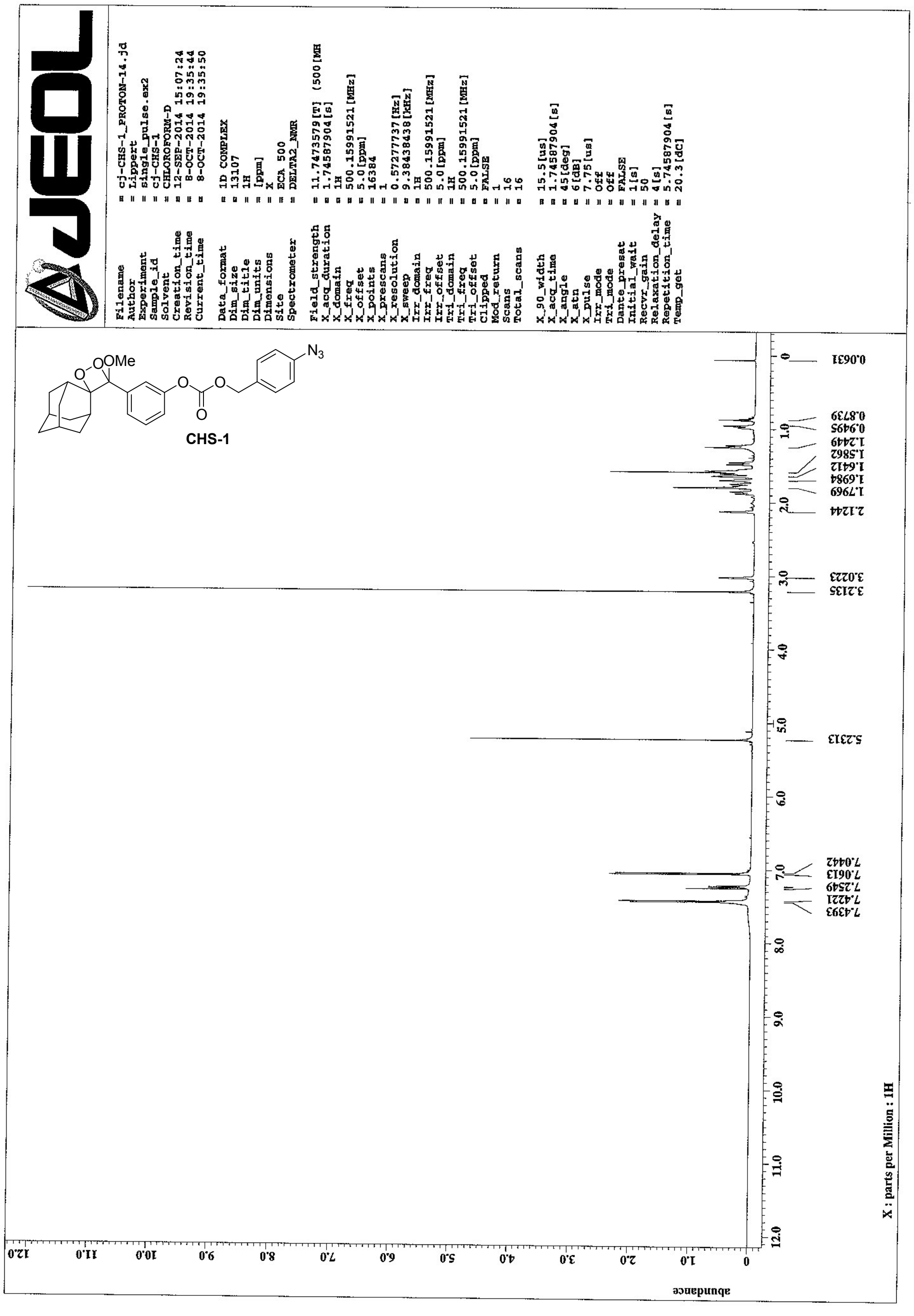
CHS-1
Filename = cj-CHS-1_PROTON-14.jd
Author = Lippert
Experiment = single_pulse.ex2
Sample_id = cj-CHS-1
Solvent = CHLOROFORM-D
Creation_time = 12-SEP-2014 15:07:24
Revision_time = 8-OCT-2014 19:35:44
Current_time = 8-OCT-2014 19:35:50
Data_format = 1D COMPLEX
Dim_size = 13107
Dim_title = 1H
Dim_units = [ppm]
Dimensions = X
Site = ECA 500
Spectrometer = DELTA2_NMR
Field_strength = 11.7473579[T] (500[MH
X_acq_duration = 1.74587904[s]
X_domain = 1H
X_freq = 500.15991521[MHz]
X_offset = 5.0[ppm]
X_points = 16384
X_prescans = 1
X_resolution = 0.57277737[Hz]
X_sweep = 9.38438438[kHz]
Irr_domain = 1H
Irr_freq = 500.15991521[MHz]
Irr_offset = 5.0[ppm]
Tri_domain = 1H
Tri_freq = 500.15991521[MHz]
Tri_offset = 5.0[ppm]
Clipped = FALSE
Mod_return = 1
Scans = 16
Total_scans = 16
X_90_width = 15.5[us]
X_acq_time = 1.74587904[s]
X_angle = 45[deg]
X_atn = 6[dB]
X_pulse = 7.75[us]
Irr_mode = Off
Tri_mode = Off
Dante_presat = FALSE
Initial_wait = 1[s]
Recvr_gain = 50
Relaxation_delay = 4[s]
Repetition_time = 5.74587904[s]
Temp_get = 20.3[dC]
0.0631
0.8739
0.9495
1.2449
1.5862
1.6412
1.6984
1.7969
2.1244
3.0223
3.2135
5.2313
7.0442
7.0613
7.2549
7.4221
7.4393
X : parts per Million : 1H
abundance

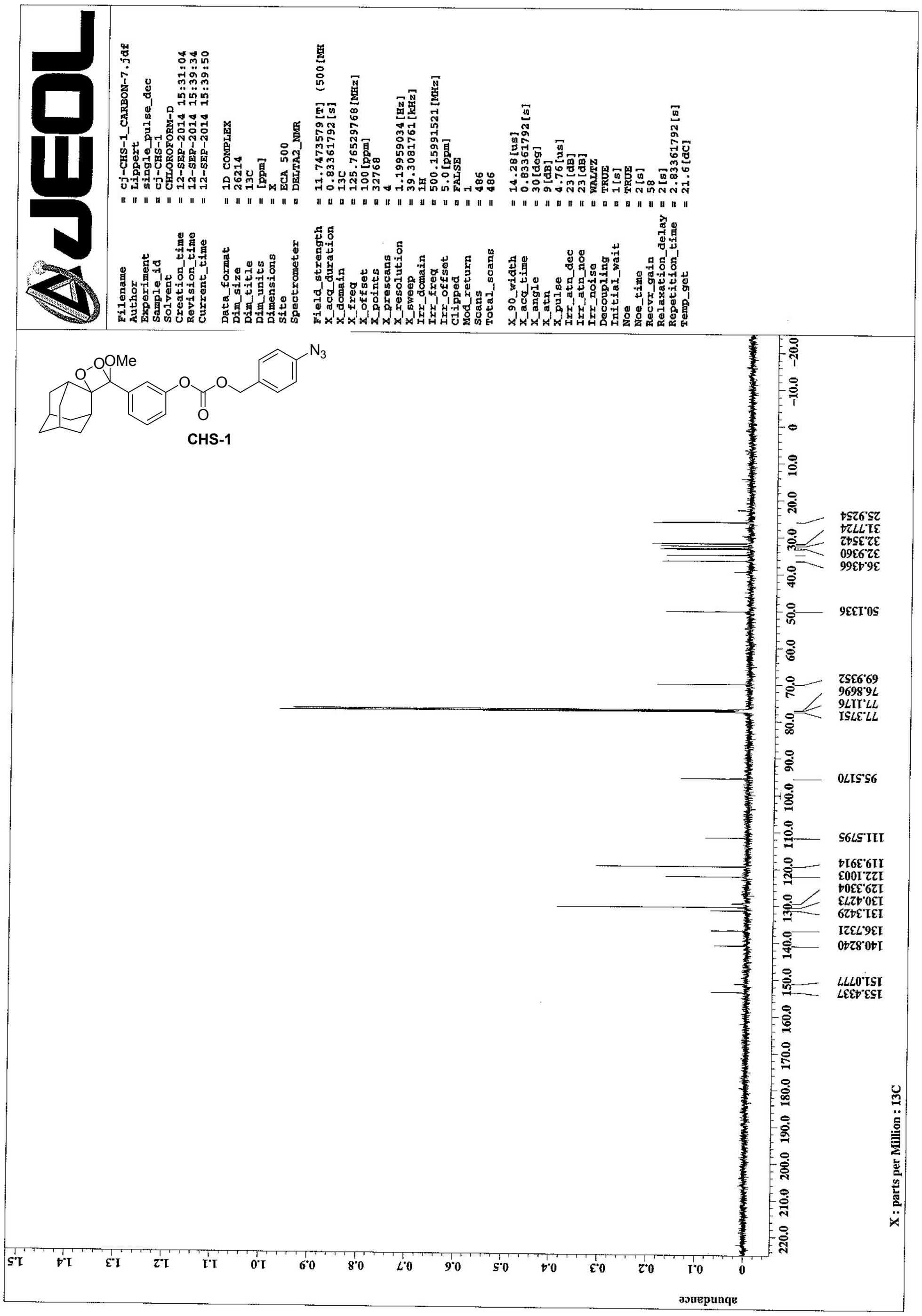
CHS-1
OMe
N_3
JEOL
Filename = cj-CHS-1_CARBON-7.jdf
Author = Lippert
Experiment = single_pulse_dec
Sample_id = cj-CHS-1
Solvent = CHLOROFORM-D
Creation_time = 12-SEP-2014 15:31:04
Revision_time = 12-SEP-2014 15:39:34
Current_time = 12-SEP-2014 15:39:50
Data_format = 1D COMPLEX
Dim_size = 26214
Dim_title = 13C
Dim_units = [ppm]
Dimensions = X
Site = ECA 500
Spectrometer = DELTA2_NMR
Field_strength = 11.7473579[T] (500[MH
X_acq_duration = 0.83361792[s]
X_domain = 13C
X_freq = 125.76529768[MHz]
X_offset = 100[ppm]
X_points = 32768
X_prescans = 4
X_resolution = 1.19959034[Hz]
X_sweep = 39.3081761[kHz]
Irr_domain = 1H
Irr_freq = 500.15991521[MHz]
Irr_offset = 5.0[ppm]
Clipped = FALSE
Mod_return = 1
Scans = 486
Total_scans = 486
X_90_width = 14.28[us]
X_acq_time = 0.83361792[s]
X_angle = 30[deg]
X_atn = 9[dB]
X_pulse = 4.76[us]
Irr_atn_dec = 23[dB]
Irr_atn_noe = 23[dB]
Irr_noise = WALTZ
Decoupling = TRUE
Initial_wait = 1[s]
Noe = TRUE
Noe_time = 2[s]
Recvr_gain = 58
Relaxation_delay = 2[s]
Repetition_time = 2.83361792[s]
Temp_get = 21.6[dC]
153.4337
151.0777
140.8240
136.7321
131.3429
130.4273
129.3304
122.1003
119.3914
111.5795
95.5170
77.3751
77.1176
76.8696
69.9352
50.1336
36.4366
32.9360
32.3542
31.7724
25.9254
X : parts per Million : 13C
abundance

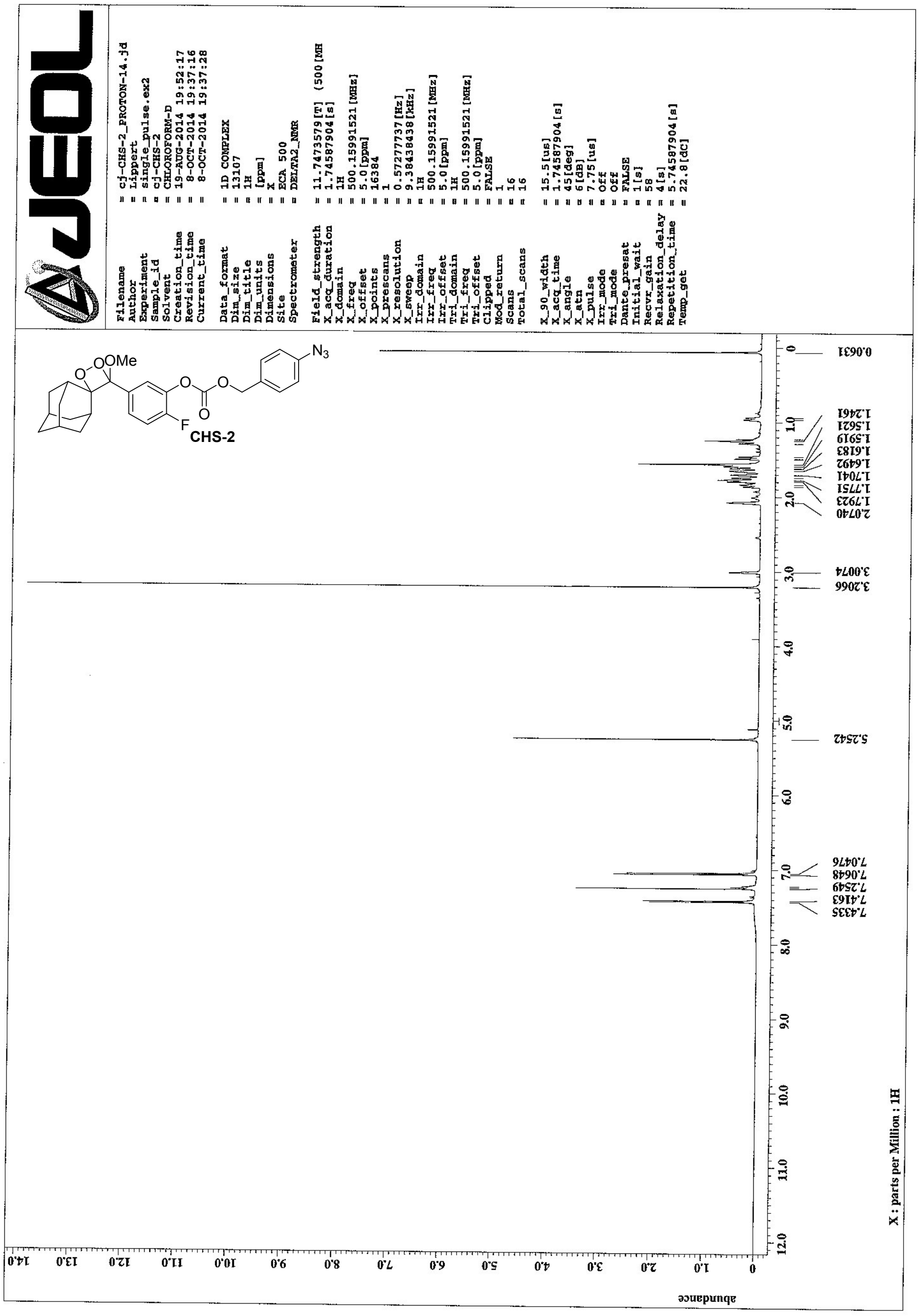
CHS-2
OMe
N3
F
Filename = cj-CHS-2_PROTON-14.jd
Author = Lippert
Experiment = single_pulse.ex2
Sample_id = cj-CHS-2
Solvent = CHLOROFORM-D
Creation_time = 19-AUG-2014 19:52:17
Revision_time = 8-OCT-2014 19:37:16
Current_time = 8-OCT-2014 19:37:28
Data_format = 1D COMPLEX
Dim_size = 13107
Dim_title = 1H
Dim_units = [ppm]
Dimensions = X
Site = ECA 500
Spectrometer = DELTA2_NMR
Field_strength = 11.7473579[T] (500[MH
X_acq_duration = 1.74587904[s]
X_domain = 1H
X_freq = 500.15991521[MHz]
X_offset = 5.0[ppm]
X_points = 16384
X_prescans = 1
X_resolution = 0.57277737[Hz]
X_sweep = 9.38438438[kHz]
Irr_domain = 1H
Irr_freq = 500.15991521[MHz]
Irr_offset = 5.0[ppm]
Tri_domain = 1H
Tri_freq = 500.15991521[MHz]
Tri_offset = 5.0[ppm]
Clipped = FALSE
Mod_return = 1
Scans = 16
Total_scans = 16
X_90_width = 15.5[us]
X_acq_time = 1.74587904[s]
X_angle = 45[deg]
X_atn = 6[dB]
X_pulse = 7.75[us]
Irr_mode = Off
Tri_mode = Off
Dante_presat = FALSE
Initial_wait = 1[s]
Recvr_gain = 58
Relaxation_delay = 4[s]
Repetition_time = 5.74587904[s]
Temp_get = 22.8[dC]
0.0631
1.2461
1.5621
1.5919
1.6183
1.6492
1.7041
1.7751
1.7923
2.0740
3.0074
3.2066
5.2542
7.0476
7.0648
7.2549
7.4163
7.4335
X : parts per Million : 1H
abundance

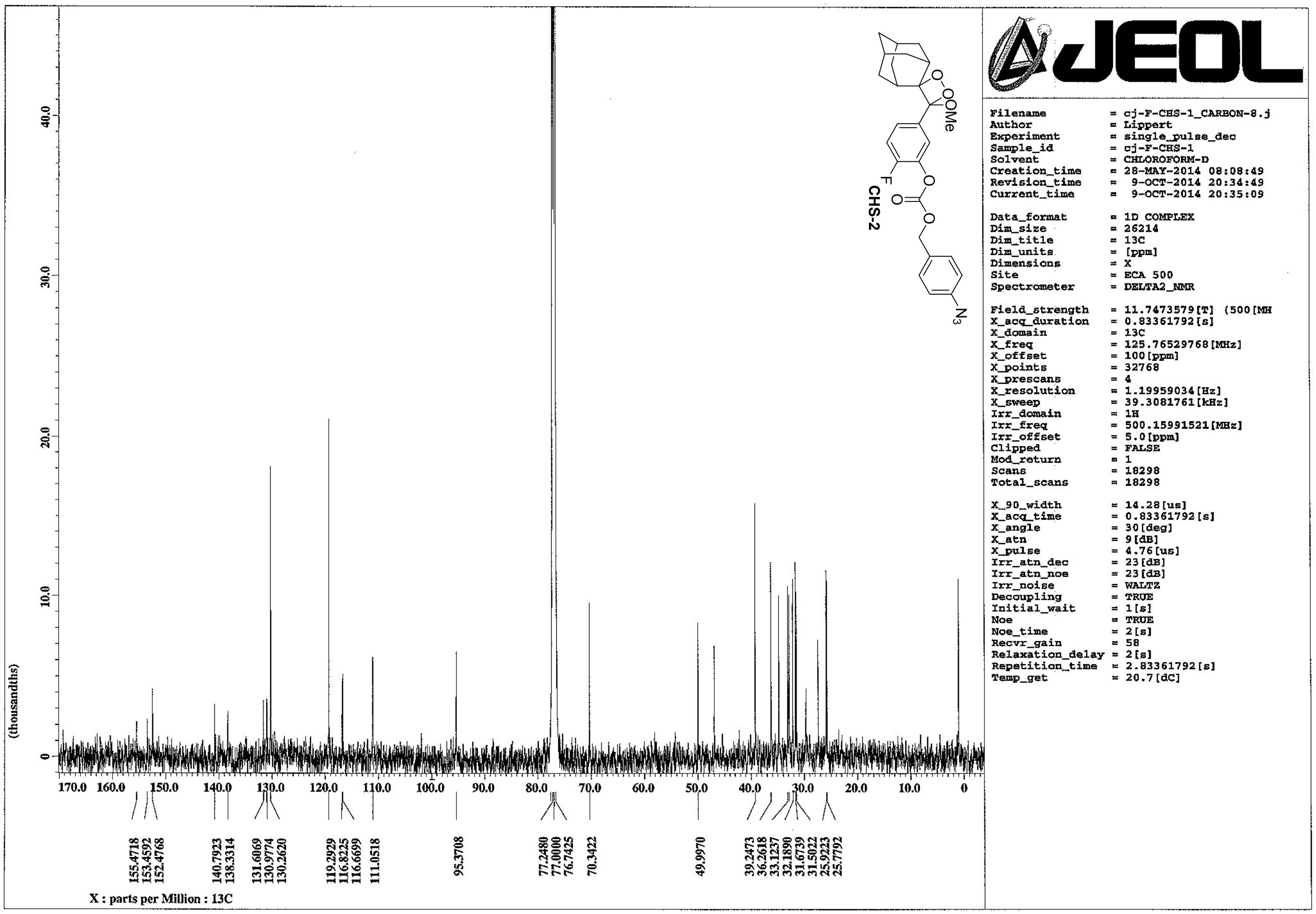
OMe
N3
F
CHS-2
JEOL
Filename = cj-F-CHS-1_CARBON-8.j
Author = Lippert
Experiment = single_pulse_dec
Sample_id = cj-F-CHS-1
Solvent = CHLOROFORM-D
Creation_time = 28-MAY-2014 08:08:49
Revision_time = 9-OCT-2014 20:34:49
Current_time = 9-OCT-2014 20:35:09
Data_format = 1D COMPLEX
Dim_size = 26214
Dim_title = 13C
Dim_units = [ppm]
Dimensions = X
Site = ECA 500
Spectrometer = DELTA2_NMR
Field_strength = 11.7473579[T] (500[MH
X_acq_duration = 0.83361792[s]
X_domain = 13C
X_freq = 125.76529768[MHz]
X_offset = 100[ppm]
X_points = 32768
X_prescans = 4
X_resolution = 1.19959034[Hz]
X_sweep = 39.3081761[kHz]
Irr_domain = 1H
Irr_freq = 500.15991521[MHz]
Irr_offset = 5.0[ppm]
Clipped = FALSE
Mod_return = 1
Scans = 18298
Total_scans = 18298
X_90_width = 14.28[us]
X_acq_time = 0.83361792[s]
X_angle = 30[deg]
X_atn = 9[dB]
X_pulse = 4.76[us]
Irr_atn_dec = 23[dB]
Irr_atn_noe = 23[dB]
Irr_noise = WALTZ
Decoupling = TRUE
Initial_wait = 1[s]
Noe = TRUE
Noe_time = 2[s]
Recvr_gain = 58
Relaxation_delay = 2[s]
Repetition_time = 2.83361792[s]
Temp_get = 20.7[dC]
(thousandths)
0
10.0
20.0
30.0
40.0
170.0 160.0 150.0 140.0 130.0 120.0 110.0 100.0 90.0 80.0 70.0 60.0 50.0 40.0 30.0 20.0 10.0 0
155.4718
153.4592
152.4768
140.7923
138.3314
131.6069
130.9774
130.2620
119.2929
116.8225
116.6699
111.0518
95.3708
77.2480
77.0000
76.7425
70.3422
49.9970
39.2473
36.2618
33.1237
32.1890
31.6739
31.5022
25.9223
25.7792
X : parts per Million : 13C

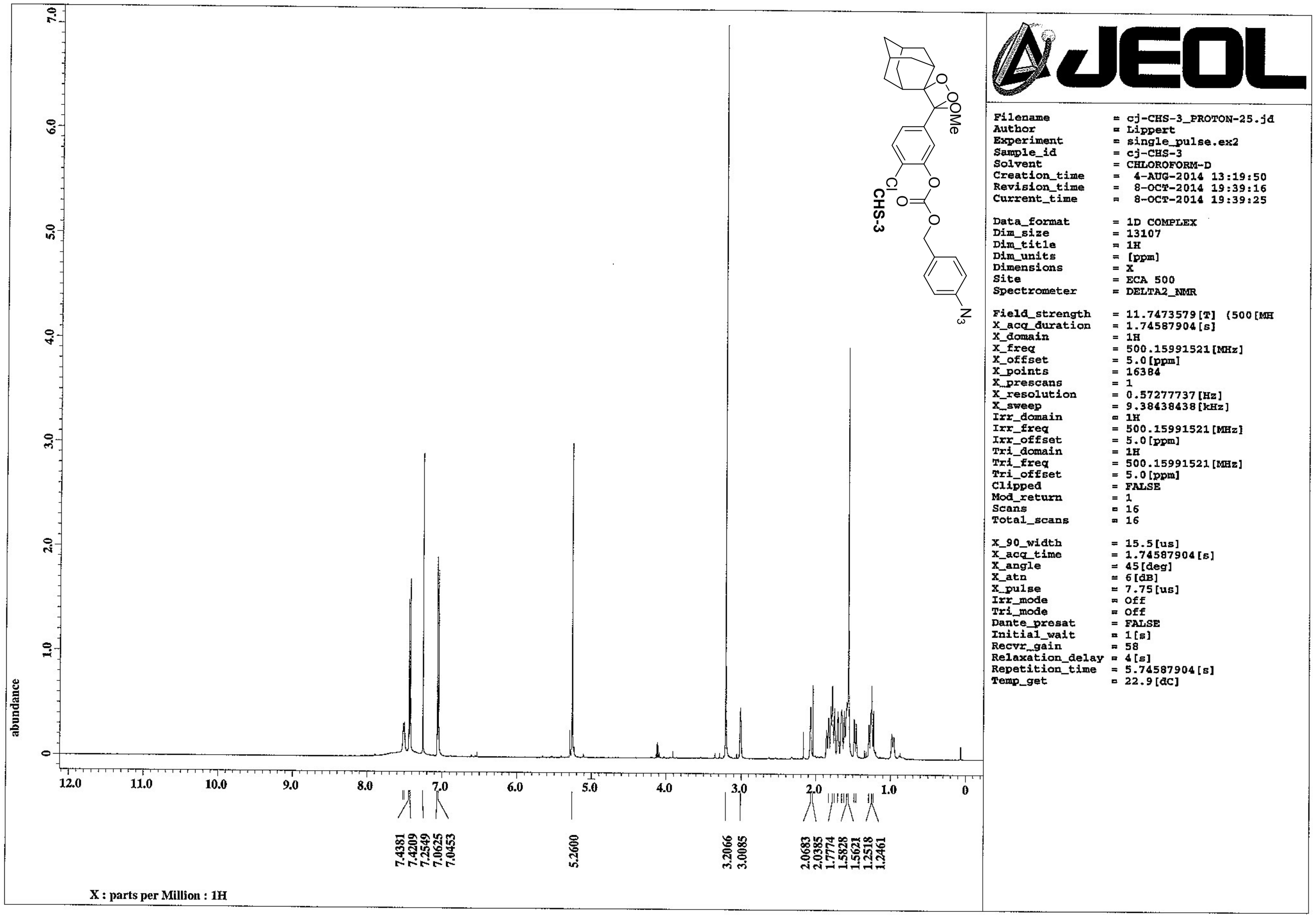
JEOL
OOMe
O
Cl
N3
CHS-3
abundance
X : parts per Million : 1H
7.4381
7.4209
7.2549
7.0625
7.0453
5.2600
3.2066
3.0085
2.0683
2.0385
1.7774
1.5828
1.5621
1.2518
1.2461
Filename = cj-CHS-3_PROTON-25.jd
Author = Lippert
Experiment = single_pulse.ex2
Sample_id = cj-CHS-3
Solvent = CHLOROFORM-D
Creation_time = 4-AUG-2014 13:19:50
Revision_time = 8-OCT-2014 19:39:16
Current_time = 8-OCT-2014 19:39:25
Data_format = 1D COMPLEX
Dim_size = 13107
Dim_title = 1H
Dim_units = [ppm]
Dimensions = X
Site = ECA 500
Spectrometer = DELTA2_NMR
Field_strength = 11.7473579[T] (500[MH
X_acq_duration = 1.74587904[s]
X_domain = 1H
X_freq = 500.15991521[MHz]
X_offset = 5.0[ppm]
X_points = 16384
X_prescans = 1
X_resolution = 0.57277737[Hz]
X_sweep = 9.38438438[kHz]
Irr_domain = 1H
Irr_freq = 500.15991521[MHz]
Irr_offset = 5.0[ppm]
Tri_domain = 1H
Tri_freq = 500.15991521[MHz]
Tri_offset = 5.0[ppm]
Clipped = FALSE
Mod_return = 1
Scans = 16
Total_scans = 16
X_90_width = 15.5[us]
X_acq_time = 1.74587904[s]
X_angle = 45[deg]
X_atn = 6[dB]
X_pulse = 7.75[us]
Irr_mode = Off
Tri_mode = Off
Dante_presat = FALSE
Initial_wait = 1[s]
Recvr_gain = 58
Relaxation_delay = 4[s]
Repetition_time = 5.74587904[s]
Temp_get = 22.9[dC]

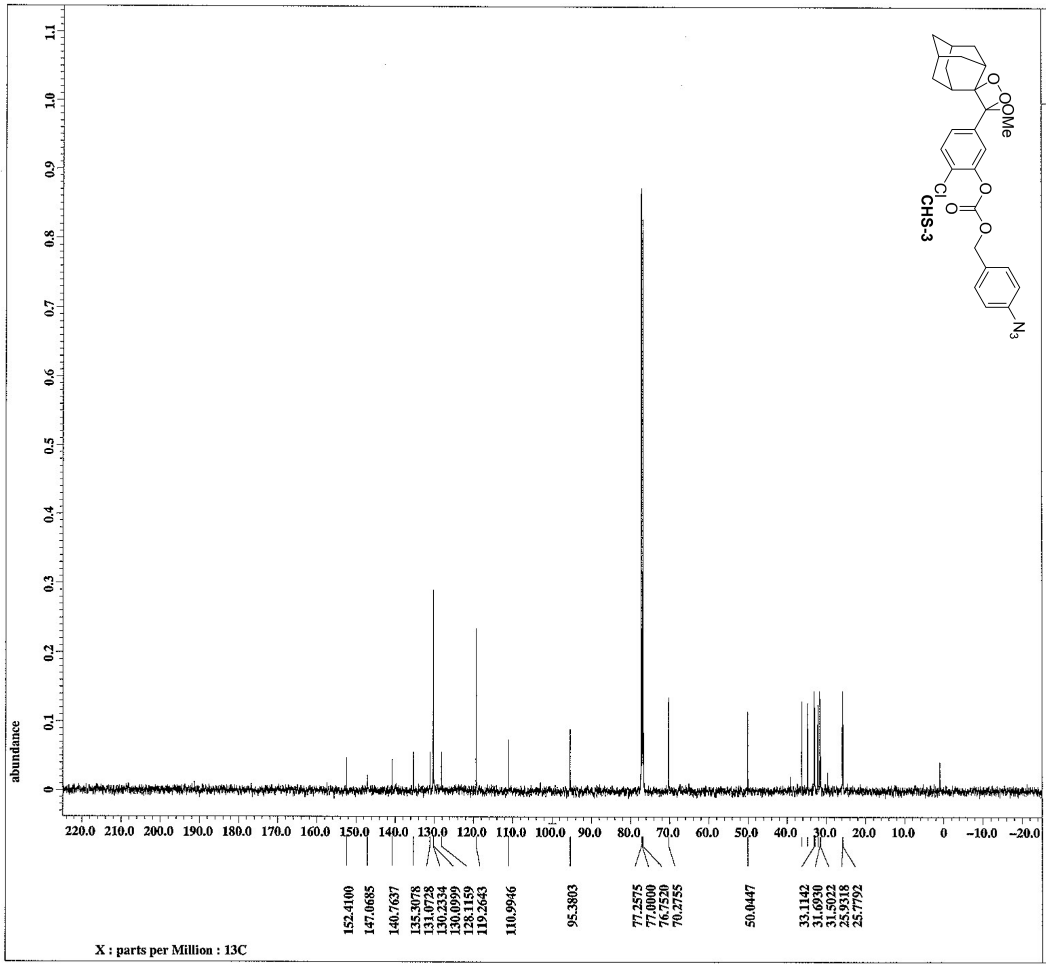

JEOL

```
Filename          = cj-CHS-3_CARBON-25.jd
Author            = Lippert
Experiment        = single_pulse_dec
Sample_id         = cj-CHS-3
Solvent           = CHLOROFORM-D
Creation_time     = 18-AUG-2014 15:26:47
Revision_time     = 10-OCT-2014 17:59:37
Current_time      = 10-OCT-2014 17:59:53

Data_format       = 1D COMPLEX
Dim_size          = 26214
Dim_title         = 13C
Dim_units         = [ppm]
Dimensions        = X
Site              = ECA 500
Spectrometer      = DELTA2_NMR

Field_strength    = 11.7473579[T] (500[MH
X_acq_duration    = 0.83361792[s]
X_domain          = 13C
X_freq            = 125.76529768[MHz]
X_offset          = 100[ppm]
X_points          = 32768
X_prescans        = 4
X_resolution      = 1.19959034[Hz]
X_sweep           = 39.3081761[kHz]
Irr_domain        = 1H
Irr_freq          = 500.15991521[MHz]
Irr_offset        = 5.0[ppm]
Clipped           = FALSE
Mod_return        = 1
Scans             = 553
Total_scans       = 553

X_90_width        = 14.28[us]
X_acq_time        = 0.83361792[s]
X_angle           = 30[deg]
X_atn             = 9[dB]
X_pulse           = 4.76[us]
Irr_atn_dec       = 23[dB]
Irr_atn_noe       = 23[dB]
Irr_noise         = WALTZ
Decoupling        = TRUE
Initial_wait      = 1[s]
Noe               = TRUE
Noe_time          = 2[s]
Recvr_gain        = 60
Relaxation_delay  = 2[s]
Repetition_time   = 2.83361792[s]
Temp_get          = 23.5[dC]
```

8. Refereneces

(1) Only 0 and 200 μM Na_2S were evaluated at pH 10.02.
(2) A. D. Becke, *J. Chem. Phys.* 1993, **98**, 5648.
(3) C. Lee, W. Yang and R. G. Parr, *Phys. Rev. B* 1988, **37**, 785.
(4) S. H. Vosko, L. Wilk and M. Nusair, *Can. J. Phys.* 1980, **58**, 1200.
(5) P. J. Stephens, F. J. Devlin, C. F. Chabalowski and M. J. Frisch, *J. Phys. Chem.* 1994, **98**, 11623.
(6) R. Krishnan, J. S. Binkley, R. Seeger, J. Pople, J. A. *J. Chem. Phys.* 1980, **72**, 650.
(7) E. Cancès, B. Mennucci, and J. Tomasi, *J. Chem. Phys*. 1997, **107**, 3032.
(8) U. C. Singh and P. A. Kollman, *J. Comp. Chem.* 1984, **5**, 129.
(9) Y. Zhao and D. G. Truhlar, *Theor. Chem. Acc.* 2008*,* **120**, 215.
(10) J. D. Chai and M. Head-Gordon, *Phys. Chem. Chem. Phys.* 2008, **10**, 6615.
(11) Gaussian 09, Revision D.01, M. J. Frisch, G. W. Trucks, H. B. Schlegel, G. E. Scuseria, M. A. Robb, J. R. Cheeseman, G. Scalmani, V. Barone, B. Mennucci, G. A. Petersson, H. Nakatsuji, M. Caricato, X. Li, H. P. Hratchian, A. F. Izmaylov, J. Bloino, G. Zheng, J. L. Sonnenberg, M. Hada, M. Ehara, K. Toyota, R. Fukuda, J. Hasegawa, M. Ishida, T. Nakajima, Y. Honda, O. Kitao, H. Nakai, T. Vreven, J. A. Montgomery, Jr., J. E. Peralta, F. Ogliaro, M. Bearpark, J. J. Heyd, E. Brothers, K. N. Kudin, V. N. Staroverov, R. Kobayashi, J. Normand, K. Raghavachari, A. Rendell, J. C. Burant, S. S. Iyengar, J. Tomasi, M. Cossi, N. Rega, J. M. Millam, M. Klene, J. E. Knox, J. B. Cross, V. Bakken, C. Adamo, J. Jaramillo, R. Gomperts, R. E. Stratmann, O. Yazyev, A. J. Austin, R. Cammi, C. Pomelli, J. W. Ochterski, R. L. Martin, K. Morokuma, V. G. Zakrzewski, G. A. Voth, P. Salvador, J. J. Dannenberg, S. Dapprich, A. D. Daniels, Ö. Farkas, J. B. Foresman, J. V. Ortiz, J. Cioslowski, and D. J. Fox, Gaussian, Inc., Wallingford CT, 2009.
